# Integration of Federated Learning and Blockchain in Health Care: Tutorial on Medical Data, Architectures, Privacy, Security, and Regulatory Compliance

**DOI:** 10.2196/80178

**Published:** 2026-06-15

**Authors:** Yahya Shahsavari, Yaser Baseri, Abdelhakim Hafid, Oussama Abderrahmane Dambri, Dimitrios Makrakis

**Affiliations:** 1 Department of Computer Science and Operations Research, Université de Montréal, 2900 Bd Édouard-Montpetit, Montréal, QC, H3T 1J4, Canada, 1 5144514964; 2 School of Electrical Engineering and Computer Science, University of Ottawa, Ottawa, ON, Canada

**Keywords:** blockchain technology, health care security, medical data privacy, IoT health care, machine learning, HIPAA compliance

## Abstract

The convergence of artificial intelligence (AI), blockchain technology, and health care represents one of the most transformative yet technically challenging frontiers in computational medicine. As health care systems adopt data-driven paradigms for precision medicine and clinical decision support, the need for secure, privacy-preserving, and collaborative learning frameworks has become critical. This tutorial introduces a comprehensive, clinically oriented, and compliance-aware framework integrating federated learning (FL) and blockchain for secure and privacy-preserving health care analytics. FL enables collaborative training across distributed institutions without raw data sharing, in alignment with privacy regulations such as the Health Insurance Portability and Accountability Act (HIPAA) and the General Data Protection Regulation (GDPR). However, FL remains vulnerable to model poisoning and gradient leakage. To address these risks, we introduce blockchain-based FL (BCFL), which leverages blockchain’s immutable ledger and decentralized consensus to enhance trust, verifiability, and auditability. The tutorial’s main contributions include (1) a taxonomy of diverse medical data types and their FL requirements; (2) three integration architectures (fully coupled, semicoupled, and loosely coupled) analyzed for security, scalability, and regulatory compliance; (3) a security analysis of health care–specific vulnerabilities and mitigation strategies using advanced cryptography, such as zero-knowledge proofs, homomorphic encryption, and differential privacy; and (4) a regulatory compliance framework addressing HIPAA, GDPR, and United States Food and Drug Administration guidelines for AI-enabled medical devices. We demonstrate BCFL’s relevance across major health care applications, including disease prediction, medical imaging, patient monitoring, and drug discovery, and highlight emerging research directions such as quantum-resilient cryptography, scalable interoperability, and automated compliance. This tutorial serves as a foundational resource for advancing secure, compliant, and collaborative AI in health care; fostering privacy-preserving analytics; and improving patient outcomes.

## Introduction

### Background

The convergence of artificial intelligence (AI), blockchain technology, and health care represents one of the most transformative yet technically challenging frontiers in computational medicine. As health care systems worldwide transition toward data-driven paradigms for precision medicine, clinical decision support, and population health management, the exponential growth in medical data generation—from electronic health records (EHRs) and medical imaging to genomic sequencing and Internet of Things (IoT)-enabled continuous monitoring—presents unprecedented opportunities for advancing health care through machine learning (ML) [1-3].

Federated learning (FL) has emerged as a promising paradigm for collaborative model training across distributed health care institutions while maintaining data locality [4,5]. However, standard FL architectures face challenges, including model poisoning, gradient leakage, and coordination failures [6-8]. Blockchain technology offers complementary capabilities through immutable audit trails, decentralized trust, and automated smart contract execution [9], yet integration with FL in health care introduces unique scalability, efficiency, and regulatory compliance challenges.

This tutorial addresses a critical gap by providing a structured, cross-disciplinary framework that integrates blockchain with FL in the context of health care, with explicit coverage of medical data taxonomies, clinical workflows, and regulatory compliance.

While prior surveys have explored the integration of FL and blockchain applications in health care and beyond [10-13], this tutorial differs in scope and emphasis. In particular, we provide (1) a systematic medical data taxonomy that classifies diverse health care data types (EHRs, imaging, genomics, biometrics, Internet of Medical Things [IoMT], clinical trials, etc) and analyzes their specific FL requirements; (2) a set of three integration architectures (fully coupled, semicoupled, and loosely coupled) with detailed evaluation of security, scalability, and compliance tradeoffs tailored to health care deployments; (3) comprehensive treatment of advanced cryptographic defenses (zero-knowledge proofs [ZKPs], homomorphic encryption [HE], and differential privacy [DP]) with health care–specific vulnerability analysis; and (4) a structured regulatory compliance framework mapping Health Insurance Portability and Accountability Act (HIPAA), General Data Protection Regulation (GDPR), and United States Food and Drug Administration (FDA) guidelines to blockchain-FL system design. Moreover, while prior surveys have typically focused on high-level concepts, our tutorial adopts a more comprehensive and tutorial-oriented scope by combining blockchain fundamentals, FL methodologies, health care–specific applications, security analysis, and forward-looking research directions. This extensive focus allows us to introduce a comprehensive, clinically oriented, and compliance-aware framework that integrates FL and blockchain for health care, providing actionable insights for both researchers and practitioners implementing secure, privacy-preserving, and regulation-compliant collaborative analytics systems. Table 1 contrasts this tutorial with earlier blockchain-based FL (BCFL) surveys and frameworks to clarify the contributions.

**Table 1.** Comparison of our tutorial with prior work on the integration of blockchain and federated learning in health care.

Reference	Medical data taxonomy	Integration architecture	Cryptography and privacy	Regulatory compliance
Abbas et al [10]	General overview	No specific architecture	Privacy mechanisms	Limited overview
Cheng et al [11]	Partial coverage	No specific architecture	Basic techniques	GDPR^a^
Ngoupayou Limbepe et al [12]	Limited scope	No specific architecture	Basic techniques	HIPAA^b^ and GDPR
Myrzashova et al [13]	Not included	No specific architecture	Security analysis	Not addressed
This paper	Comprehensive taxonomy	Three distinct architectures	Security and privacy analysis	HIPAA, GDPR, and FDA^c^

a GDPR: General Data Protection Regulation.

b HIPAA: Health Insurance Portability and Accountability Act.

c FDA: Food and Drug Administration.

### Motivation and Problem Statement

Contemporary health care faces a fundamental paradox: the increasing need for large-scale, collaborative data analytics to advance medical knowledge is juxtaposed with stringent privacy regulations and the inherent sensitivity of medical data. Traditional centralized approaches to health care ML create critical vulnerabilities where patient information aggregated in single repositories becomes an attractive target for sophisticated attacks [14,15]. Recent breaches affecting millions of patient records underscore the inadequacy of current security models, particularly when regulatory frameworks, such as HIPAA and GDPR, and emerging national data protection laws impose severe penalties for data mishandling [16].

While FL addresses data locality concerns by enabling distributed model training, it introduces health care–critical vulnerabilities. Model poisoning attacks could compromise diagnostic accuracy [6,7], potentially leading to misdiagnosis or incorrect treatment recommendations. Gradient leakage exploits can enable patient reidentification from shared model updates [8,17], violating privacy guarantees that health care institutions must maintain. Coordination failures in distributed learning processes could disrupt critical clinical workflows [18], affecting real-time decision support systems.

Blockchain technology addresses these vulnerabilities through immutable audit trails that ensure accountability, decentralized trust establishment that eliminates single points of failure, automated smart contract execution for verifiable protocol compliance, and comprehensive data provenance tracking [9]. However, health care–specific integration challenges remain, including scalability constraints when processing high-frequency medical data updates from IoMT devices, energy efficiency requirements for sustainable long-term deployment in resource-constrained clinical environments, compliance with medical device regulations for AI/ML systems, and real-time performance requirements for clinical decision support applications.

Existing BCFL approaches [19-23] are predominantly designed for generic data types and fail to address health care–specific requirements. Critical gaps include a lack of regulatory compliance frameworks for HIPAA and GDPR, insufficient integration with clinical workflows and existing health IT systems, inadequate support for multimodal medical data with heterogeneous privacy requirements, and absence of explainability mechanisms required for medical decision-making. This tutorial fills these gaps by providing a comprehensive framework tailored specifically for health care applications.

### Contributions and Novelty

This tutorial advances the state-of-the-art at the intersection of blockchain technology, FL, and health care informatics through several methodological and theoretical contributions:

1. Comprehensive medical data taxonomy for FL: We develop a systematic classification of medical data types (EHRs, imaging, genomics, biometrics, IoT sensors, clinical trials, etc) and their specific requirements for FL implementations, including privacy sensitivity levels, regulatory considerations, and technical constraints for distributed processing.
2. Novel integration architecture framework: We introduce and formally define 3 distinct BCFL integration architectures (fully coupled, semicoupled, and loosely coupled) specifically designed for health care environments, with detailed analysis of their security guarantees, scalability characteristics, and regulatory compliance capabilities.
3. Health care–specific security and privacy analysis: We provide a comprehensive security analysis of BCFL systems in health care contexts, identifying unique vulnerabilities related to medical data sensitivity, regulatory requirements, and clinical workflow dependencies, along with corresponding mitigation strategies.
4. Regulatory compliance framework: We systematically examine how BCFL architectures can satisfy complex health care regulations (HIPAA, GDPR, and FDA guidelines for AI/ML-based medical devices), providing practical guidance for implementation in real-world health care settings.
5. Research roadmap and future directions: We identify critical research gaps at the intersection of blockchain, FL, and health care, proposing specific research directions that could accelerate adoption and address current technical limitations.

### Tutorial Scope and Target Audience

This tutorial is designed for researchers and practitioners at the intersection of health care informatics, distributed systems, and ML. Our target audience includes (1) health care informaticians seeking to implement privacy-preserving collaborative analytics, (2) computer scientists developing secure FL systems, (3) blockchain researchers exploring health care applications, (4) clinical researchers interested in multi-institutional collaborations, and (5) regulatory and policy experts working on health care data governance. The tutorial assumes familiarity with basic ML concepts but provides a comprehensive background on FL and blockchain technologies. We emphasize practical implementation considerations while maintaining rigorous technical depth appropriate for publication in top-tier venues.

### Organization

**Figure 1.**
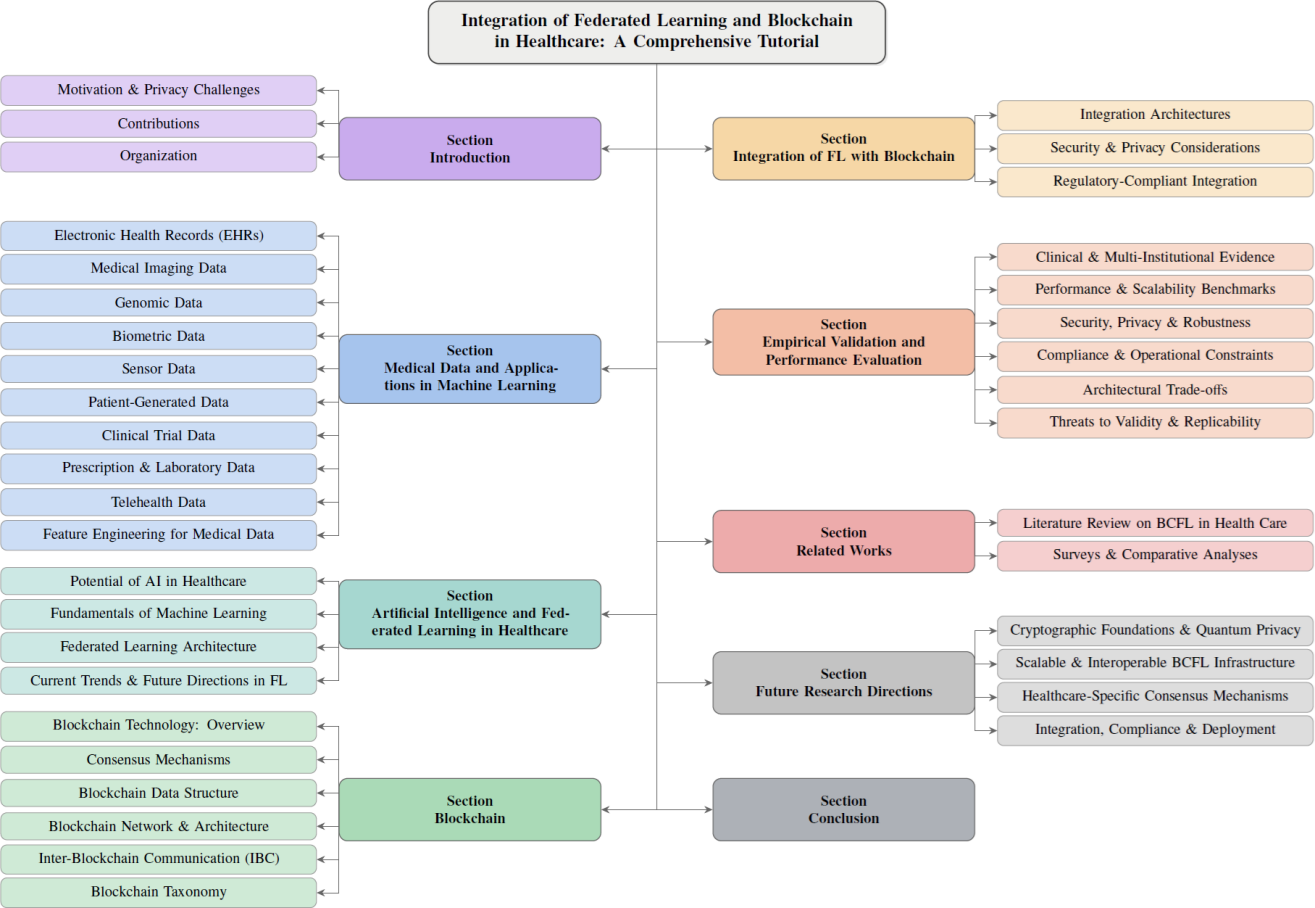
Organizational structure of the tutorial.

The remainder of this tutorial is structured as illustrated in Figure 1. The Medical Data and Applications in ML section introduces a comprehensive taxonomy of medical data types, including EHRs, imaging, genomics, and sensor data, and discusses their relevance to ML. The AI and FL in Health Care section explores the evolution of AI in health care, highlighting FL paradigms, their challenges, and future trends. The Blockchain section reviews fundamental blockchain concepts, such as consensus, data structures, and cross-chain communication, with a focus on health care applicability. The Integration of FL With Blockchain section presents integration architectures that combine FL and blockchain, along with their security, privacy, and regulatory implications in health care. The Empirical Validation and Performance Evaluation section synthesizes quantitative evidence from clinical deployments, benchmarks, and adversarial robustness studies to provide empirical validation, assess performance tradeoffs, and establish the framework’s real-world feasibility. The Related Work section offers a critical review of previous work on the integration of blockchain and FL to contextualize the contributions of this tutorial. The Future Research Directions section synthesizes emerging research challenges and opportunities, grouped under cryptographic resilience, infrastructure scalability, health care–specific consensus and incentives, and regulatory compliance in system integration. Finally, the Conclusion section summarizes the key findings and reflects on the real-world translation of BCFL systems into clinical practice.

## Medical Data and Applications in ML

### Overview

Medical data encompass a diverse array of information crucial for understanding, diagnosing, and treating various health conditions. These data, ranging from patient demographics and medical history to diagnostic images and genomic sequences, hold immense potential for advancing health care through ML applications. By harnessing the power of ML algorithms, medical data can be analyzed to extract valuable insights, predict patient outcomes, personalize treatments, and optimize health care delivery. However, the utilization of medical data for ML requires careful consideration of data storage and management practices to ensure compliance with privacy regulations, maintain data integrity, and facilitate seamless access for research and clinical purposes. This section explores the different types of medical data and their applications in ML.

### EHR Data

EHRs are digital patient charts containing medical history, diagnoses, medications, treatment plans, immunization dates, allergy information, medical images, and lab results, providing a comprehensive, up-to-date view for informed decision-making [24,25]. The key EHR features include the following: (1) *digital format*, electronic records replace paper for easier storage, access, and sharing; (2) *interoperability*, designed for sharing among different providers and organizations, facilitating better care coordination; (3) *real-time access*, authorized professionals get quick access to critical information, which is crucial for emergencies; (4) *patient engagement*, features allow patients to access data, schedule appointments, and communicate with providers; (5) *decision support*, tools offer alerts for drug interactions, screening reminders, and clinical guidelines; and (6) *data security and privacy*, security measures protect confidentiality, with access restricted to authorized personnel [26]. EHRs are a rich source of structured and unstructured data for ML applications. ML algorithms analyze this comprehensive patient information to extract insights, predict outcomes, identify patterns, and improve clinical decision-making [27,28]. In ML, EHR data are used for tasks, including the following: (1) *predictive analytics*, where models forecast medical events (eg, hospital readmission, disease onset, and mortality) for proactive, personalized care [29,30]; (2) *disease identification and diagnosis*, where algorithms assist in early detection by identifying subtle patterns and anomalies, aiding accurate and timely diagnoses [31,32]; and (3) *treatment recommendations*, where models suggest personalized plans based on history, demographics, genetics, and past responses to optimize outcomes [33].

### Medical Imaging Data

Medical imaging data encompass diverse visual representations of internal body structures, acquired via distinct imaging modalities [34,35] for clinical scrutiny, diagnosis, and ongoing assessment. The key components of medical imaging data are (1) patient information, (2) imaging modalities, (3) image files, and (4) reports. First, patient information includes basic demographic and identifying details (eg, name, ID, age, and gender). Second, among imaging modalities, each modality uses specific physical principles [36] to generate images adapted to clinical needs. The imaging modalities include (1) radiography for projection imaging of osseous structures [37]; (2) computed tomography (CT) for high-resolution cross-sectional imaging using x-ray and computational reconstruction [38]; (3) magnetic resonance imaging (MRI) for detailed soft tissue visualization via magnetic fields and radiofrequency pulses [39]; (4) ultrasonography for real-time acoustic wave imaging, which is ideal for soft tissues and dynamic processes [40]; (5) nuclear medicine imaging for visualizing organ function via radiopharmaceuticals [41]; (6) positron emission tomography for quantitative functional imaging, especially in oncology, neurology, and cardiology [42]; and (7) fluoroscopic imaging for dynamic, real-time x-ray visualization during interventional and functional studies [43]. Third, regarding image files, medical data, including metadata (patient information, acquisition parameters, and image details) [44], are stored, shared, and transmitted according to technical standards [45], notably Digital Imaging and Communications in Medicine (DICOM) [46]. Fourth, reports include narrative documents generated by radiologists summarizing image analysis findings for the treatment planning of referring physicians. Medical imaging data have a wide range of ML applications in health care [47], including the following: (1) *disease diagnosis and classification*, ML algorithms assist in the diagnosis and classification of diseases like cancer and neurological, cardiovascular, and musculoskeletal disorders [48]; (2) *computer-aided detection*, computer-aided detection systems use ML to help radiologists detect abnormalities (eg, tumors, lesions, and fractures), improving diagnostic accuracy and efficiency [49]; (3) *image-based biomarker discovery*, ML identifies imaging biomarkers associated with diseases or treatment responses, which is valuable for prognosis, efficacy assessment, and personalized medicine [50,51]; (4) *treatment planning and monitoring*, imaging data are used to develop personalized plans, and ML predicts outcomes, monitors progression, and optimizes treatment strategies [52]; (5) *image reconstruction and enhancement*, ML techniques (eg, deep learning) improve image quality from various modalities (MRI, CT, and ultrasonography) by reducing artifacts and enhancing resolution for better interpretation [53]; (6) *image registration and fusion*, ML algorithms automatically align and combine multiple images (from different modalities or time points) for comprehensive visualization and analysis; and (7) *drug discovery and development*, ML analyzes imaging data to evaluate drug effects on disease progression, identify potential drug targets, and optimize delivery methods [54].

### Genomic Data

Genomic data pertain to information concerning the organization and operation of the genome within an organism [55,56]. In health care ML, this refers to comprehensive information from an individual’s genome (entire DNA/genetic composition), offering profound insights into genetic predispositions, treatment responses, and overall health [57]. The key components for medical use cases include the following: (1) *genome sequences*, the complete set of genetic material (DNA), consisting of nucleotides (A, T, C, and G) that encode genetic information; (2) *genes*, specific DNA sequences that encode instructions for protein synthesis, including their location, structure, and function; (3) *single nucleotide polymorphisms*, single-nucleotide variations that can influence traits, disease susceptibility, and drug responses, along with their associated phenotypic and disease information [58]; (4) *copy number variations*, genomic alterations involving changes in the number of copies of DNA segments, affecting gene dosage and leading to phenotypic variations and disease susceptibility [59]; (5) *gene expression profiles*, information on the activity levels of different genes (often generated via microarrays or RNA sequencing [60]), providing insights into protein synthesis; (6) *epigenetic modifications*, heritable changes in gene expression (eg, DNA methylation and histone modifications) that do not alter the DNA sequence but influence gene activity and phenotype [61]; and (7) *genetic variation databases*, compilations of genomic data, genetic variants, annotations, and associated phenotypic information from various sources (eg, population studies and disease databases) [62]. These components are essential for understanding the genetic basis of diseases, identifying risk factors, and developing personalized health care approaches. The utilization of genomic data in health care ML includes the following: (1) *disease prediction and risk stratification*, ML algorithms scrutinize genomic data to discern patterns and variations linked to specific diseases, assessing associated health risks [63]; (2) *personalized medicine*, genomic data form the basis for personalized treatment plans [64,65], and ML models predict individual medication responses for tailored strategies [66-68]; (3) *drug discovery and computational genomics*, ML analyzes genomic data to expedite discovery, identifying drug targets and comprehending genetic underpinnings for more efficacious therapeutic solutions [69]; (4) *genomic counseling*, ML algorithms decipher complex genomic data, assisting health care professionals in communicating intricate genetic details, risks, and familial implications [70]; (5) *early detection and diagnostics*, integrating genomic data and ML algorithms facilitates early detection by discerning subtle genetic variations indicative of specific conditions, enabling timely intervention [71,72]; (6) *research and population health informatics*, aggregated genomic data subjected to ML can advance the understanding of disease genetic foundations at the population level, informing public health initiatives and epidemiological studies [73]; and (7) *genomic sequencing and computational analysis*, ML plays a crucial role in interpreting extensive genomic datasets from sequencing technologies, identifying genetic mutations, variations, and other health-impacting information [74].

### Biometric Data

Biometric data in the medical context refer to unique physical or behavioral characteristics used for individual identification and monitoring. These data are crucial in health care for patient identification, medical record access control, personalized treatments, and health condition monitoring. The components of biometric data include the following: (1) *physiological biometrics*, data types like fingerprints, facial recognition, iris/retina scans, DNA, smell recognition, and hand geometry data [75]; (2) *behavioral biometrics*, data types such as voice recognition, gait analysis, and typing dynamics data [75]; (3) *health-related biometrics*, data types such as heart rate, blood pressure, blood oxygen levels, electrocardiography (ECG) patterns [76], and brain wave patterns (electroencephalography [EEG] patterns) [77]; and (4) *analytical biometric technologies*, advanced modalities like microbial biometrics, which analyze unique microbiome compositions for identification and health assessment [78], and olfactory biometrics, which utilize distinctive body odor profiles for identification and disease detection [79].

Biometric data integrated into health care ML encompass quantifiable physiological or behavioral attributes [75,80] for accurate identification [81,82], secure access [83,84], and health assessment [85]. The primary applications of biometric data in health care ML include the following: (1) *biometric identification and access control*, biometric features (eg, fingerprints and facial characteristics) are pivotal for precise identification and heightened security, governing access to sensitive areas, EHRs, and medical devices [80,86]; (2) *patient identification and record matching*, biometric identifiers (eg, fingerprints and iris scans) ensure precise linkage of patients to their health records, minimizing errors, with ML algorithms enhancing matching accuracy and elevating care quality [87-90]; (3) *biometric monitoring for health assessment*, continuous monitoring of biometric data (eg, heart rate and ECG data) via wearables/sensors facilitates real-time health assessment, and ML analyzes dynamic data for the early detection of anomalies, supporting timely intervention and personalized management [91-93]; (4) *behavioral biometrics for mental health monitoring*, behavioral biometrics (eg, typing and voice modulation) contribute to assessing mental health and detecting behavioral changes [94,95], and ML models discern patterns indicative of conditions, aiding targeted interventions [96]; (5) *biometric data in clinical trials*, biometric data are used for participant identification, monitoring, and data integrity, and ML assists in efficient management and analysis, ensuring study validity [97,98]; (6) *voice and speech analysis for diagnostics*, ML algorithms process voice patterns to detect potential markers for conditions like Parkinson disease [99,100] or respiratory disorders [101,102], contributing to diagnostic capabilities [103]; and (7) *facial recognition for patient monitoring*, facial recognition is used for monitoring patient well-being and distress, and ML analyzes facial expressions, offering insights into comfort levels and enhancing care quality [104,105].

### Sensor Data

Sensor data in medical contexts represent information collected from various devices (wearable, implantable, or environmental devices) to monitor health, detect condition changes, and assist diagnoses. These data continuously track physiological parameters, activity levels, and environmental conditions. The components of medical sensor data include the following: (1) *wearable sensors*, data from devices like heart rate monitors, blood pressure monitors, blood glucose monitors, activity trackers, and pulse oximeters; (2) *implantable sensors*, data from devices like cardiac monitors, glucose monitors, and intraocular pressure sensors; (3) *environmental sensors*, data gathered from air quality monitors (for pollutants and allergens affecting respiratory conditions) and temperature/humidity sensors; (4) *specialized medical sensors*, data from ECG, EEG, electromyography, gait sensors, and sleep monitors; and (5) *smart health homes*, data extracted from fall detectors and bed sensors that monitor sleep patterns, bed occupancy, and vital signs.

Sensor data used in health care ML are collected from various sources (wearables, medical equipment, and monitoring tools) [106,107], providing real-time health and activity insights. ML algorithms analyze these data to make predictions, identify patterns, and offer personalized insights. The utilization of sensor data in health care ML includes the following: (1) vital sign monitoring, (2) wearable devices for activity tracking, (3) blood glucose monitoring and continuous glucose monitoring, (4) ECG monitoring, (5) sleep monitoring, (6) environmental sensors, (7) medication adherence monitoring, (8) fall detection and activity recognition, and (9) biometric sensors for stress and emotion monitoring. First, in vital sign monitoring, ML analyzes continuous vital sign data (heart rate, blood pressure, etc) to detect anomalies, predict health deteriorations, and offer early warnings [108,109]. Second, regarding wearable devices for activity tracking, ML models analyze data from accelerometers and gyroscopes to assess physical health, detect abnormalities, and provide personalized insights for fitness and rehabilitation [110]. Third, in blood glucose monitoring and continuous glucose monitoring, ML algorithms analyze continuous glucose monitoring data to predict glucose level trends, recommend insulin dosages, and enhance diabetes management [111]. Fourth, in ECG monitoring, ML interprets ECG data to identify cardiac abnormalities, predict cardiovascular risk, and recommend early interventions [112,113]. Fifth, in sleep monitoring, ML algorithms analyze sleep data (from wearables and bed sensors) to identify disorders, provide insights into sleep hygiene, and recommend personalized interventions [114,115]. Sixth, regarding environmental sensors, ML correlates environmental data (air quality and temperature) with health outcomes, aiding in identifying triggers for respiratory conditions or allergies [116]. Seventh, in medication adherence monitoring, ML analyzes adherence patterns tracked by smart sensors, sending reminders and providing providers with compliance insights [117,118]. Eighth, regarding fall detection and activity recognition, ML models use motion sensor data for fall risk assessment, accident prediction, and adapting care plans for individuals with mobility challenges [119]. Ninth, regarding biometric sensors for stress and emotion monitoring, ML analyzes biometric signals (eg, skin conductance and heart rate variability) to provide insights into mental health, stress management, and emotional well-being [120,121].

### Patient-Generated Data

Patient-generated data (PGD) in health care ML refer to health-related information actively contributed by patients [122,123], distinct from traditional clinical records. Sourced directly via wearables, mobile apps, and patient-reported outcomes (PROs), PGD foster a patient-centric, data-driven approach, enabling personalized interventions, early detection, and improved patient-provider communication [124]. Beyond sensor data, PGD utilization in health care ML includes the following: (1) *mobile health apps and surveys*, patients input health information and feedback via apps, and ML processes these data to derive insights into treatment effectiveness, medication adherence, and satisfaction, informing personalized care plans [125]; (2) *social media and online communities*, patients share health experiences and concerns online [126], and ML conducts analyses on social media data for health-related trends, sentiment, and public health monitoring, contributing to population health research and patient perspectives [127,128]; (3) *genomic and genetic data sharing*, patients voluntarily contribute genetic information for research, and ML analyzes aggregated genomic data to identify genetic factors associated with diseases, fostering precision medicine advancements [129]; and (4) *telehealth and virtual visits*, patients offer health updates during virtual consultations, and ML algorithms analyze PGD from these visits to support clinical decision-making, monitor treatment progress, and enhance virtual health care quality [130].

### Clinical Trial Data

Clinical trial data refer to information systematically collected during trials designed to evaluate the safety, efficacy, or effectiveness of new medical interventions (drugs, devices, and procedures) in human participants [131]. These data, governed by a structured protocol, inform medical decision-making, regulatory approvals, and medical knowledge advancements. Integrating these data into ML models enables the development of predictive algorithms, risk assessment tools, and decision support systems, contributing to evidence-based medicine, personalized treatments, and enhanced clinical research efficiency [132]. The key components of clinical trial data are as follows: (1) demographic information, (2) informed consent, (3) medical history, (4) intervention details, (5) clinical assessments, (6) adverse events, (7) efficacy endpoints, (8) follow-up data, and (9) protocol deviations. First, demographic information includes details on study participants (age, gender, race, and ethnicity) incorporated into ML models to assess intervention response across groups and develop personalized treatment plans. Second, informed consent involves documentation confirming that participants were informed of risks/benefits and voluntarily agreed to participate, ensuring ethical standards and regulatory compliance, with ML incorporating this to restrict the analysis to consented data [133,134]. Third, medical history includes information on pre-existing conditions, relevant history, and concurrent medications, which is used to identify comorbidities that may impact treatment outcomes. Fourth, intervention details include specifics about the product/procedure (dosage, administration, and protocol), with ML analyzing these details to identify patterns associated with treatment success or failure, predicting efficacy in future cases [135]. Fifth, clinical assessments include physical examinations, lab tests, imaging, and measurements to assess the health status and intervention response, with ML processing this information to identify trends, correlations, or anomalies indicative of treatment responses or adverse events, aiding early detection and prediction [136]. Sixth, adverse events involve records of side effects experienced, including severity and relation to the intervention, with ML learning from these historical data to predict the likelihood of adverse events for new interventions, supporting risk assessment and proactive management [137,138]. Seventh, efficacy endpoints include measurements (eg, symptom relief and disease marker improvement) used to determine intervention effectiveness, with ML analyzing these data to develop predictive models for treatment success/failure and identify key contributing factors. Eighth, follow-up data include information on long-term outcomes, adherence, and sustained effects collected during posttrial visits, which is essential for longitudinal analyses, with ML using this information to predict the long-term effects of interventions, including sustained efficacy or potential relapse [139]. Ninth, protocol deviations involve documentation of deviations from the original plan and their reasons, with ML analyzing the data to identify how deviations impact study outcomes, allowing for adjustment or prediction of their potential effects [140].

### Prescription and Medication Data

Prescription and medication data in medical records relate to prescribed drugs, dosage, frequency, and related details. These data are crucial for patient safety, medication management, monitoring public health and treatment efficacy, research, regulatory compliance, and facilitating communication. Integrated into EHRs, they provide a comprehensive medication history, allowing health care professionals to make informed decisions and avoid potential adverse events. Aggregated and anonymized data are used in research to broadly assess medication safety and effectiveness. The key components of prescription and medication data are as follows: (1) patient information; (2) prescriber information; (3) prescription date; (4) medication name; (5) dosage, frequency, and route of administration; (6) duration of treatment; (7) instructions for use; (8) refill information; (9) allergies and contraindications; (10) adverse reactions; (11) medication changes; (12) medication discontinuation; and (13) medication administration records (MARs). First, patient information includes identification details (name, date of birth, and demographics), which are used in ML for personalized treatment. Second, prescriber information includes details about the prescribing provider, with ML models being trained to recognize prescribing practices associated with positive patient outcomes [141]. Third, prescription date is the date the prescription was issued, and temporal analysis assists in predicting medication adherence and treatment outcomes, with ML identifying patterns related to issuance timing and patient behavior [142]. Fourth, medication name involves generic and brand names, with ML models categorizing medications by therapeutic class, aiding in identifying commonalities in treatment outcomes [143]. Fifth, dosage, frequency, and route of administration include the prescribed amount/strength, how often it should be taken, and the administration method, which are crucial details for predicting adherence and adverse events, with ML models identifying optimal regimens [144]. Sixth, duration of treatment is the prescribed period, with ML models analyzing this to predict long-term outcomes, including treatment success and potential drug resistance [145]. Seventh, instructions for use include additional guidance (eg, taken with food and specific times), with natural language processing (NLP) approaches extracting insights from free-text instructions to identify nuances in patient guidance [146]. Eighth, refill information includes details on authorized refills, which are essential for predicting adherence and persistence, with ML identifying factors influencing refill behavior and predicting discontinuation likelihood [147]. Ninth, allergies and contraindications involve records of known patient allergies or contraindications, with ML identifying associations among these records, adverse drug reactions, and specific medications to predict safety and suitability for individual patients [148]. Tenth, adverse reactions involve documentation of experienced side effects, with data being analyzed to develop predictive models identifying patients at higher risk for specific side effects, enabling proactive management. Eleventh, medication changes include information about dosage adjustments or switches, with ML analyzing historical changes to predict future treatment modifications, assisting in personalized planning [129,149]. Twelfth, medication discontinuation involves the reason and date of stopping medication, with ML models predicting factors contributing to discontinuation, helping providers intervene to improve adherence [150]. Thirteenth, MARs include records of actual medication administration in a health care setting, with MAR data being used to train ML models for predicting administration patterns and identifying deviations from the prescribed regimen [151].

### Laboratory Data

Laboratory data in medical records are the results of tests and analyses conducted on patient samples, which are essential for diagnosing, monitoring, and managing various medical conditions. The types of data collected vary by patient presentation and provider assessment. ML techniques (supervised, unsupervised, and deep learning) are applied depending on the nature of the data and analysis goals. The common types of laboratory data are the results of the following tests: (1) blood tests, (2) urinalysis, (3) microbiology tests, (4) pathology tests, (5) hematology tests, (6) immunology tests, (7) endocrine tests, (8) serology tests, (9) genetic tests, and (10) radiology and imaging studies. First, blood tests include assessments of the complete blood count (cell counts), blood chemistry panel (electrolytes, glucose, and organ function tests), and lipid profile, with ML models identifying patterns in complete blood count results associated with specific diseases (eg, anemia and infections) [152]. Second, urinalysis involves examination of the physical/chemical properties of urine, with ML algorithms detecting patterns indicative of kidney disorders, urinary tract infections, or diabetes [153]. Third, microbiology tests involve identifying microorganisms and their antibiotic sensitivity, with ML assisting in microorganism identification from culture data and predicting antibiotic susceptibility for personalized treatment [154,155]. Fourth, pathology tests include tissue biopsy and cytology (cell examination) performed for disease/abnormality diagnosis (eg, cancer), with image recognition ML models trained on pathology slides assisting in identifying abnormal tissue or cancerous cells [50,156]. Fifth, hematology tests include coagulation studies (blood clotting) and erythrocyte sedimentation rate (an inflammation measure), with ML models analyzing coagulation data to predict the risk of bleeding or clotting disorders [157]. Sixth, immunology tests include antibody tests (detecting immune system antibodies) and viral load (measuring the virus in blood), with ML algorithms identifying patterns in antibody levels to diagnose autoimmune or infectious conditions [158,159]. Seventh, endocrine tests include assessments of hormone levels (eg, thyroid and insulin), with ML models analyzing these levels to predict and monitor endocrine disorders like thyroid dysfunction or diabetes [160,161]. Eighth, serology tests include analyses of serum components (proteins, enzymes, and electrolytes), with ML identifying markers associated with specific diseases, aiding early detection and monitoring [158]. Ninth, genetic tests involve the identification of specific genetic markers for condition diagnosis, with ML analyzing these data to identify disease risk, predict treatment response, or diagnose genetic disorders [70]. Tenth, radiology and imaging studies include diagnostic tests contributing to medical data, with image recognition/segmentation ML models being trained on radiology images to assist in diagnosing conditions like tumors or fractures.

### Telehealth Data

Telehealth data refer to information collected during remote health care interactions between patients and providers [162]. These data are crucial in modern health care, utilizing technology (video, phone, and online platforms) to deliver services remotely and contributing to the overall patient record [163]. The key components of telehealth data are as follows: (1) audio and video recordings, (2) text-based communication, (3) diagnostic and monitoring device data, (4) EHR integration, (5) appointment and scheduling data, (6) patient demographics and consent, (7) prescription and medication data, and (8) PROs. First, audio and video recordings include recordings of virtual consultations used for documentation and quality assurance, with ML analyzing these for sentiment or clinical insights, speech recognition transcribing audio for verbal analysis, and facial recognition/sentiment analysis assessing patient emotions and engagement [164]. Second, text-based communication involves chat logs, texts, or emails containing symptom and treatment information, with NLP extracting and categorizing information (symptoms and treatment discussions) and sentiment analysis gauging patient satisfaction and well-being [165]. Third, diagnostic and monitoring device data include data from remote devices (eg, blood pressure monitors, glucose meters, and wearables) that enable continuous remote health monitoring, with ML models analyzing trends and patterns, and time series analysis and predictive modeling detecting trends/anomalies in vital signs and predicting exacerbations of chronic conditions [166,167]. Fourth, EHR integration involves integration into EHRs/electronic medical records for a comprehensive patient view, with ML analyzing combined data to identify correlations between in-person and virtual interactions, improving diagnosis and treatment planning. Fifth, appointment and scheduling data include information on scheduling, duration, and attendance, with analysis optimizing appointment availability and patient access, and predictive analytics optimizing scheduling and resource allocation by anticipating demand. Sixth, patient demographics and consent include details about the patient and consent for services, ensuring privacy compliance and personalized care context, with ML analyzing demographic data for population health trends, targeted interventions, and service personalization. Seventh, prescription and medication data include information on prescriptions, management, and adherence, supporting virtual refills, with predictive modeling analyzing adherence patterns to inform personalized interventions and identifying medication-related risks and interactions [168]. Eighth, PROs include patient-reported data on symptoms, well-being, and effectiveness, with ML analyzing PROs via text mining and sentiment analysis to predict treatment responses and assess correlations with clinical outcomes to inform decisions [169].

### Feature Engineering for Medical Data

While raw medical data provide the foundation for downstream ML applications, they often require substantial transformation to become analytically useful. Feature engineering refers to the process of extracting, selecting, and constructing meaningful features from raw data to enhance model accuracy, interpretability, and generalizability [170]. This step is particularly critical in the health care domain due to the inherent heterogeneity, noise, and sparsity of clinical data.

The feature engineering process encompasses several essential techniques tailored to different data modalities. Normalization and encoding transform categorical variables and scale numerical values to comparable ranges. Temporal aggregation captures time-dependent patterns from longitudinal records such as EHR data. NLP embedding methods convert unstructured clinical notes into dense vector representations. Signal processing techniques extract meaningful patterns from physiological sensors and medical imaging. Dimensionality reduction methods address the curse of dimensionality while preserving informative variance in high-dimensional genomic or imaging data.

**Figure 2.**
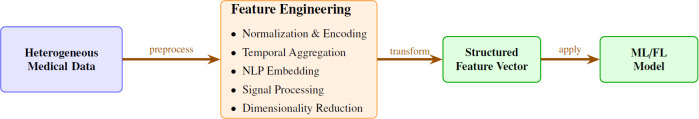
Feature engineering pipeline for heterogeneous medical data. Raw health care data from 10 diverse sources (detailed taxonomy in Figure 3) undergo systematic preprocessing and transformation to generate unified feature vectors for downstream machine learning (ML) or federated learning (FL) applications. NLP: natural language processing.

**Figure 3.**
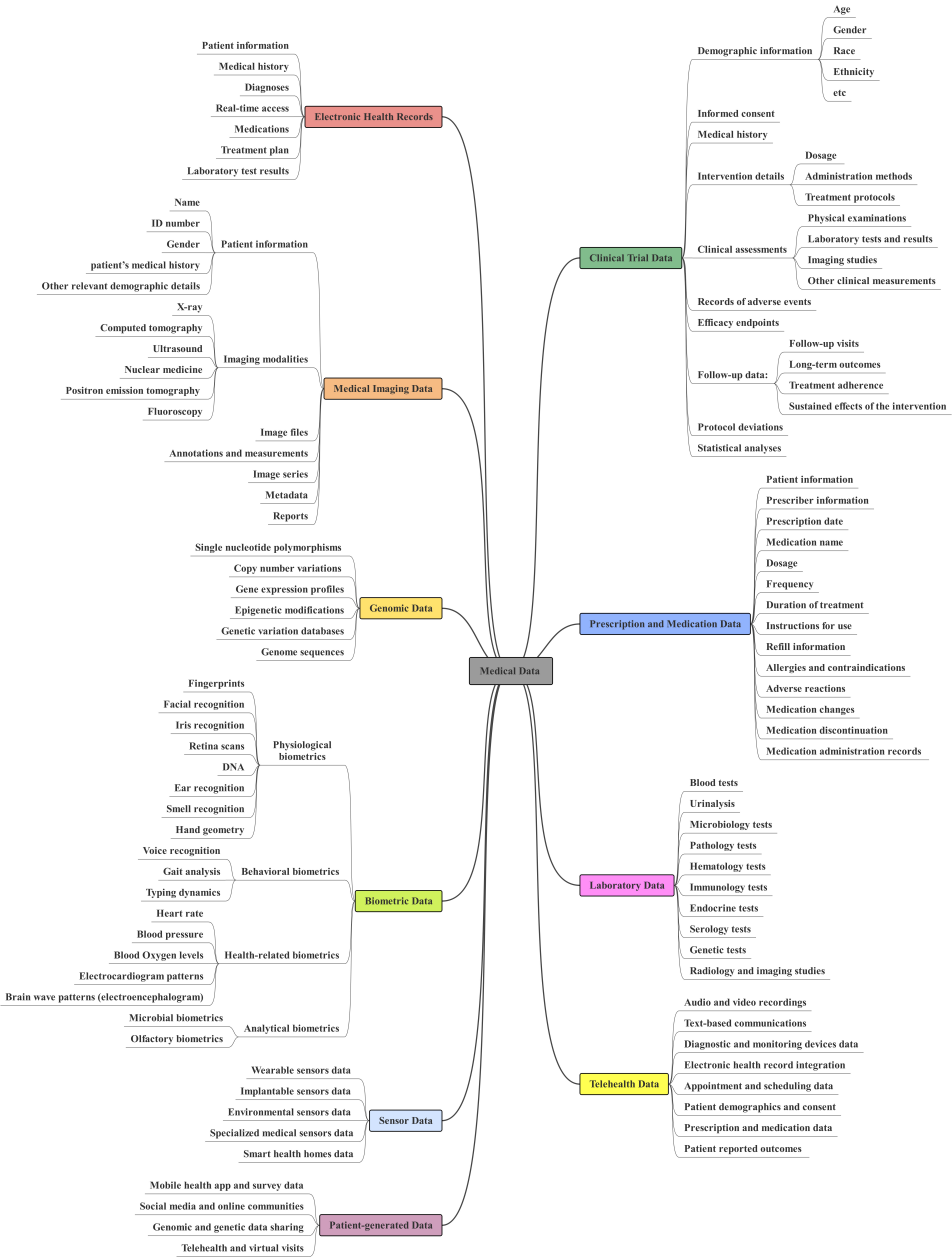
Medical data taxonomy for health care machine learning.

Effective feature engineering not only improves model performance but also enhances interpretability and supports reproducibility across diverse health care settings [171]. In FL scenarios, harmonizing feature spaces across distributed clients is crucial to ensure compatible model training and aggregation [171]. Figure 2 illustrates a high-level pipeline that uses systematic preprocessing transformations for mapping heterogeneous raw medical data (detailed taxonomy in Figure 3) to structured feature vectors suitable for downstream ML and FL modeling.

## AI and FL in Health Care

### Overview

AI is revolutionizing health care, impacting diagnosis, treatment, and patient care. This section explores the evolution of AI in health care, from its historical roots to cutting-edge developments, showcasing its potential across various health care domains. We will further delve into FL as a key technology for the future. FL’s collaborative approach enables privacy-preserving data analysis, making it a powerful tool for health care research and delivery. We will discuss its applications, case studies, current challenges, and future directions.

### Potential of AI in Health Care

**Figure 4.**
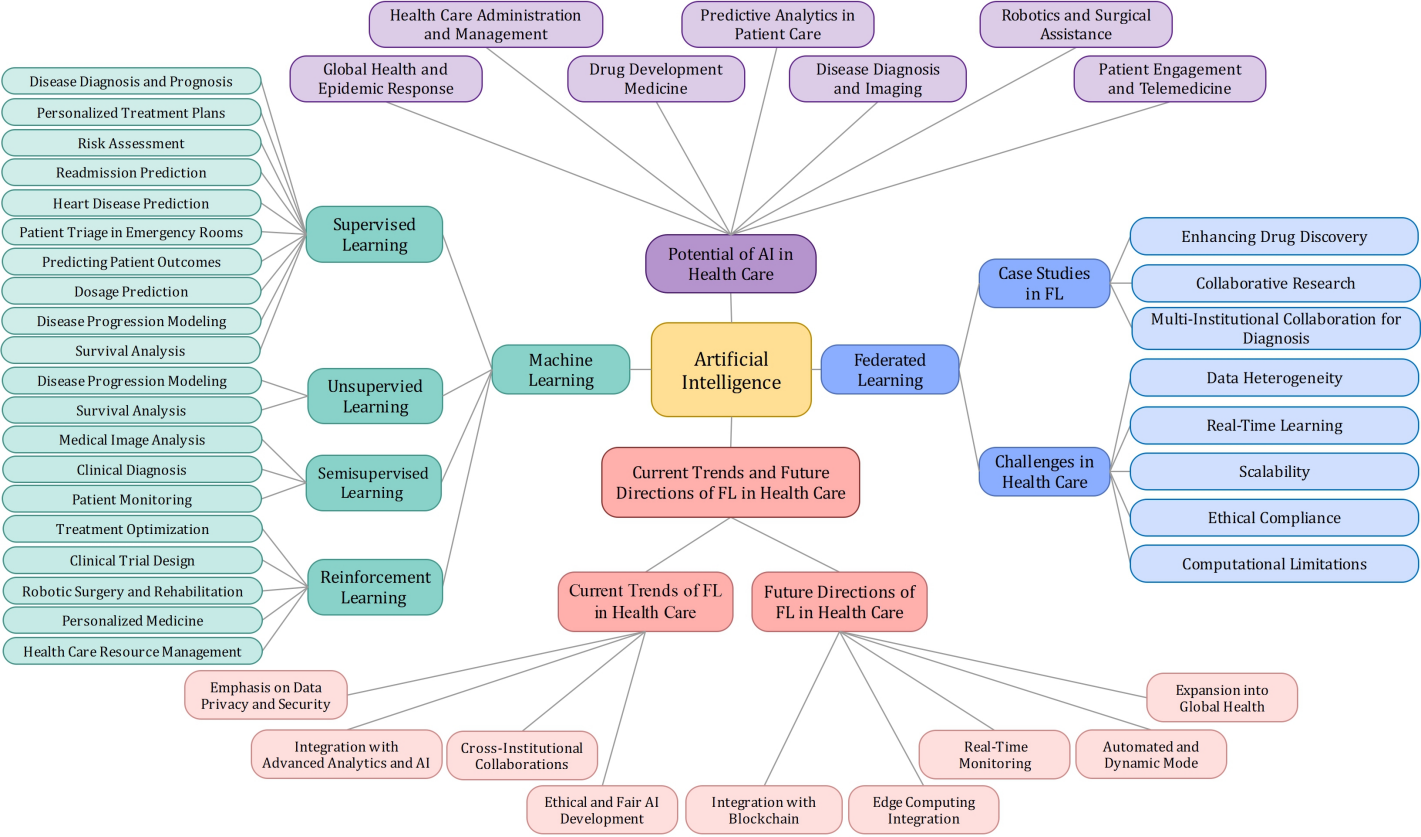
Exploring the convergence of artificial intelligence (AI) with health care: trends, applications, and future perspectives. FL: federated learning.

**Figure 5.**
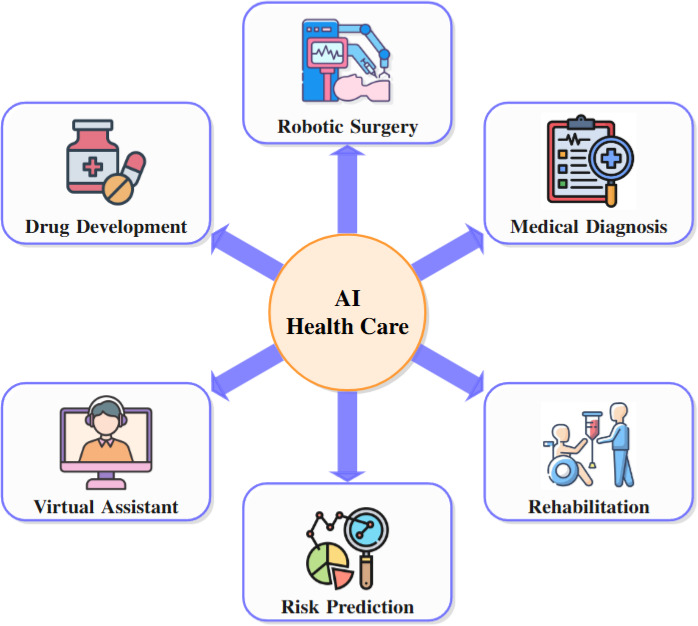
Six key application domains of artificial intelligence (AI) in health care. AI technologies enable advances across drug discovery, surgical precision, diagnostic accuracy, patient rehabilitation, risk assessment, and virtual health assistance.

AI is transforming health care across a wide spectrum, from disease diagnosis to patient care management, making significant contributions in several key areas (Figure 4). The first area is *disease diagnosis and imaging*. AI has revolutionized medical imaging, providing more accurate and faster diagnoses [172]. Deep learning models, trained on large datasets (eg, x-ray images [173], MRI scan images [174], and CT scan images [175]), can identify patterns undetectable to the human eye, aiding in the early detection of diseases like cancer, cardiovascular abnormalities, and neurological disorders. The second area is *drug development and personalized medicine*. AI algorithms streamline drug development by predicting molecular behavior [176] and identifying potential drug candidates [177]. In personalized medicine, AI analyzes patient data, including genetic information, to tailor treatments, improving efficacy and reducing side effects [123,178]. The third area is *predictive analytics in patient care*. AI’s predictive analytics are crucial to preventive medicine. EHRs are analyzed [179] to predict patient risks for diseases [180], hospital readmission [181], and adverse events, enabling proactive care [180]. The fourth area is *robotics and surgical assistance*. AI-integrated robotics improve surgical precision and outcomes [182]. AI-driven robots assist surgeons in complex procedures, reducing human error and recovery time, and AI supports surgeon training through virtual reality simulations [183]. The fifth area is *patient engagement and telemedicine*. AI-powered chatbots and virtual health assistants provide 24/7 support and health monitoring [184], enhancing patient engagement and adherence. In telemedicine, AI tools assist in remote diagnosis and consultation, making health care more accessible [185]. The sixth area is *health care administration and management*. AI streamlines administrative tasks (scheduling, billing, and claims) [186] and optimizes hospital operations, resource allocation, and patient flow, improving overall efficiency and reducing costs [187]. The seventh area is *global health and epidemic response*. AI is pivotal in tracking and predicting the spread of infectious diseases [188]. During the COVID-19 pandemic, AI models were instrumental in analyzing virus transmission [189], assessing vaccine development [190], and managing health care resources [191]. The key application domains of AI in health care are presented in Figure 5.

### Fundamentals of ML

#### ML Background

ML, a pivotal branch of AI, is fundamentally reshaping our approach to problem-solving across various domains, including health care. At its core, ML involves the development and application of algorithms that enable computers to learn from and make decisions or predictions based on data. This capacity for self-improvement and adaptation without explicit programming is what sets ML apart.

The cornerstone of ML is data. Algorithms learn from data patterns, and the quality and quantity of these data significantly influence their performance, as explained in the previous section. ML algorithms are sets of rules or instructions given to computers to help them learn from data. These algorithms can be broadly categorized into supervised learning [192-197], unsupervised learning [198-205], semisupervised learning [206-209], and reinforcement learning (RL) [210-215].

#### Supervised Learning

Supervised learning, a dominant branch of ML, is crucial in health care, leveraging labeled datasets to train models for making predictions or categorizing data [192]. This approach is powerful where the relationship between the input and output is known and can be modeled [216]. The key characteristics include the following: (1) *labeled data*, training data have known outcomes, guiding the algorithm to learn the relationship between input features and the output; (2) *classification and regression*, the two primary tasks are classification (predicting discrete outcomes, eg, diagnosing a disease) and regression (predicting continuous outcomes, eg, recovery time); and (3) *model training and validation*, the model is trained on one portion of data and validated on a separate, unseen dataset to ensure good generalization.

Supervised learning has a significant impact on health care applications, including the following: (1) *disease diagnosis and prognosis*, ML models are trained on clinical data (symptoms, lab results, and imaging) to identify diseases (eg, models trained on imaging data can detect abnormalities like tumors in radiographic images with high accuracy) [193,194]; (2) *personalized treatment plans*, algorithms analyze patient data to predict individual responses to different treatments, which is effective in fields like oncology [195] where plans are tailored based on tumor genetics; (3) *risk assessment*, models predict the risk of developing conditions (eg, diabetes and heart disease) based on lifestyle, genetics, and other factors [196]; and (4) *readmission prediction*, supervised learning predicts a patient’s likelihood of hospital readmission, which is vital for improving patient care and reducing costs [197].

ML offers invaluable tools through its classification and regression tasks. Classification entails categorizing data into predetermined classes, and this is crucial for the following: (1) *disease diagnosis*, models classify patient data into disease categories (eg, using convolutional neural networks to classify dermatological images as benign or malignant skin lesions) [217]; (2) *heart disease prediction*, algorithms analyze patient data (age, blood pressure, and cholesterol level) to classify individuals into risk categories for heart disease, aiding early intervention [218]; and (3) *patient triage in emergency rooms*, models classify patients based on condition severity (analyzing symptoms, vitals, and history) to assist in determining urgency, optimizing patient flow and resource allocation [219].

Regression tasks deal with predicting continuous outcomes in health care, and they are applied to the following: (1) *predicting patient outcomes*, models predict quantitative outcomes like length of hospital stay, surgical recovery time, or disease progression (eg, predicting blood sugar levels in diabetic patients based on lifestyle/medication) [220]; (2) *dosage prediction*, regression algorithms predict the optimal drug dosage for individual patients [144], which is particularly important in treatments like chemotherapy to balance efficacy and toxicity [221]; (3) *disease progression modeling*, models forecast the rate of progression for chronic diseases (eg, Alzheimer disease and Parkinson disease) by analyzing patient data over time, aiding treatment planning [222]; and (4) *survival analysis*, regression models are crucial in oncology for predicting patient survival times after diagnosis or treatment, which is vital for planning and management [223].

#### Unsupervised Learning

Unsupervised learning is a fundamental ML category that analyzes and groups unlabeled data based on similarities and differences, without predefined labels [198]. Two critical techniques are clustering and dimensionality reduction [199-201,203,205,224], and both are vital in genomics and medical imaging.

Clustering groups objects so that those in the same cluster are more similar to each other than to those in other groups. Its health care applications include the following: (1) *genomic data analysis*, clustering categorizes genes with similar expression patterns, aiding in understanding gene functions, identifying disease markers, and revealing biological pathways (eg, it identifies co-expressed gene groups in diseases like cancer, revealing potential therapeutic targets) [199]; (2) *patient stratification*, algorithms segment patients into groups based on similarities in medical records or genetic information [224], and this stratification identifies disease subtypes with distinct clinical outcomes or treatment responses, facilitating personalized medicine; and (3) *medical imaging*, clustering is used for image segmentation (partitioning images into pixel sets) to identify regions of interest, such as tumors in MRI or CT scans, which is crucial for accurate diagnosis and treatment planning [201].

Dimensionality reduction decreases the number of random variables by obtaining a set of principal variables, which is important for dealing with high-dimensional data common in health care as follows: (1) *genomic data analysis*, genomic data are inherently high-dimensional, with techniques like principal component analysis [202] and t-distributed stochastic neighbor embedding [203] reducing their complexity, and this simplification aids in data visualization, identifying genetic markers, and understanding the genetic architecture of diseases; and (2) *medical imaging*, high-resolution images are computationally intensive, and dimensionality reduction techniques reduce the number of features while retaining essential information [204]. This is crucial for efficient storage, processing, and analysis of medical images, facilitating the development of more efficient diagnostic algorithms [205].

#### Semisupervised Learning

Semisupervised learning is a fundamental ML category uniquely positioned in health care because it improves model performance by leveraging both labeled and unlabeled data [206,208,225]. This approach is cost-effective, utilizing the abundance of unlabeled data in health care where labeling is often expensive or time-consuming. The key features include the following: (1) *utilizing unlabeled data*, algorithms exploit the vast amounts of unlabeled data in health care databases, which, despite lacking explicit annotations, contain valuable information that complements labeled data and enhances the model’s understanding of complex medical phenomena [226]; (2) *combining labeled and unlabeled data*, by incorporating both data types during training, semisupervised algorithms learn more robust representations of the underlying data distribution [227], improving the model’s generalization capabilities and leading to more accurate predictions; and (3) *semisupervised techniques*, methods like self-training, co-training, and semisupervised support vector machines iteratively refine predictions using labeled data while leveraging unlabeled data to enhance overall performance [207].

In health care, semisupervised learning applies to diverse areas as follows: (1) *medical image analysis*, algorithms analyze large volumes of unlabeled medical images to identify subtle patterns or anomalies, and combining this unsupervised analysis with labeled data improves the accuracy of tasks such as tumor detection, organ segmentation, and disease classification [209]; (2) *clinical diagnosis*, it assists diagnosis and outcome prediction by leveraging both labeled patient data and unlabeled population health data [208], and this integrated approach enhances diagnostic accuracy and reliability, leading to more informed clinical decisions; and (3) *patient monitoring*, techniques analyze large streams of unlabeled patient data (eg, EHRs and physiological signals) to detect deviations from normal health patterns [225], and incorporating these data into predictive models allows providers to proactively identify and intervene in adverse health events. Overall, semisupervised learning offers a powerful framework to leverage the wealth of unlabeled health care data, advancing ML model performance, medical research, diagnosis, and treatment strategies [228,229].

#### Reinforcement Learning

RL is an ML type particularly suited for situations where an “agent” must make a sequence of decisions to achieve a goal [230]. Approaches to learning optimal policies include the following: (1) *dynamic programming*, breaks down decision-making into simpler, recursively solved subproblems, which is effective in environments with a perfect, known model (states and transitions); and (2) *Monte Carlo methods*, model-free approaches relying on repeated random sampling to approximate the optimal policy, which is useful for problems with stochastic dynamics and rewards.

In health care, RL offers innovative ways to approach complex, dynamic decision-making problems [210], operating on reward and penalty principles to learn optimal actions through trial and error. The key concepts include the following: (1) *agent and environment*, an “agent” (eg, a health care model) interacts with its “environment” (eg, patient data/scenarios), taking actions and receiving feedback in the form of rewards or penalties based on the outcomes; (2) *policy*, the strategy the agent uses to determine the next action based on the current environment state (eg, choosing a treatment plan); (3) *reward signal*, guides the agent’s actions, with positive rewards encouraging similar decisions and negative rewards signaling adjustment; (4) *value function*, estimates the expected cumulative reward of taking an action in a given state, helping the agent predict long-term outcomes; and (5) *exploration versus exploitation*, balancing trying new actions (exploration) with using known high-reward actions (exploitation), which is analogous to balancing experimental and tried-and-tested treatments.

RL is highly useful in health care applications, including the following: (1) *treatment optimization*, RL adjusts treatment strategies over time based on patient response [211], and for chronic diseases like diabetes, models suggest adaptive insulin dosages, diet, and exercise plans; (2) *clinical trial design*, RL helps determine the most effective trial structures, treatment regimens, and patient selection criteria, enhancing trial efficiency and success rates [231-233]; (3) *robotic surgery and rehabilitation*, used in training robotic systems to adapt to patient-specific conditions and improve based on feedback from surgical outcomes or recovery rates [212]; (4) *personalized medicine*, RL models analyze patient data over time to predict the most effective treatment plans, considering the unique health trajectory and response patterns of each patient [213,214]; and (5) *health care resource management*, algorithms assist in managing resources (hospital bed allocation, staff scheduling, and equipment usage) by learning optimal allocation strategies based on demand and resource availability [215].

#### Case Studies of ML in Health Care

ML has made significant inroads into health care, transforming patient care, diagnostics, treatment planning, and disease management. Notable real-world examples highlight both successes and challenges as follows: (1) *diagnostic imaging and radiology*, ML (especially deep learning) has revolutionized medical image interpretation (eg, Google Health’s ML model outperformed human radiologists in detecting breast cancer in mammograms [234]), and the challenges include integrating these systems into clinical workflows, dealing with diverse data quality, and ensuring consistent performance across different populations and equipment; (2) *drug discovery and development*, ML accelerates drug discovery, reducing cost and time (eg, Atomwise used AI to predict effective molecules, even identifying promising COVID-19 compounds in 2020 [235]), and the primary challenge is the time-consuming and expensive process of validating AI-discovered drugs in clinical trials; (3) *predictive analytics in patient care*, ML models are increasingly used for predictive analytics (eg, the Johns Hopkins team used ML to predict sepsis in hospitalized patients, enabling early intervention [236]), and the challenges include ensuring data privacy, overcoming data silos, and dealing with potential biases in training data; (4) *personalized medicine*, ML aids in tailoring treatments to individual genetic profiles (eg, a team at Earle A Chiles Research Institute used ML to analyze the genetic data of cancer patient for identifying the most effective treatment plans [237]), and the challenges include managing vast amounts of genetic data, ensuring accurate interpretations, and integrating these insights into routine clinical practice; and (5) *mental health applications*, ML models monitor and diagnose mental health conditions (eg, apps like Ginger.io use ML to analyze user interaction and provide personalized mental health support [238]), and the challenges include addressing privacy concerns, ensuring algorithm sensitivity and specificity across diverse populations, and integrating these tools with traditional mental health services.

### FL Details

#### FL Background

Within the field of ML, data security and privacy are paramount concerns. Traditional ML approaches often require centralized data storage, which can raise privacy issues and limit participation due to data ownership restrictions. FL has emerged as a groundbreaking solution, offering a decentralized paradigm for collaborative ML [239]. In FL, multiple entities, such as health care institutions or research centers, collaborate to train a model without sharing their raw data. Each entity trains a local model on its own data and shares only model updates, such as gradients or parameters, with a central server. This collaborative approach allows for distributed learning while preserving data privacy and security.

#### FL Types

**Figure 6.**
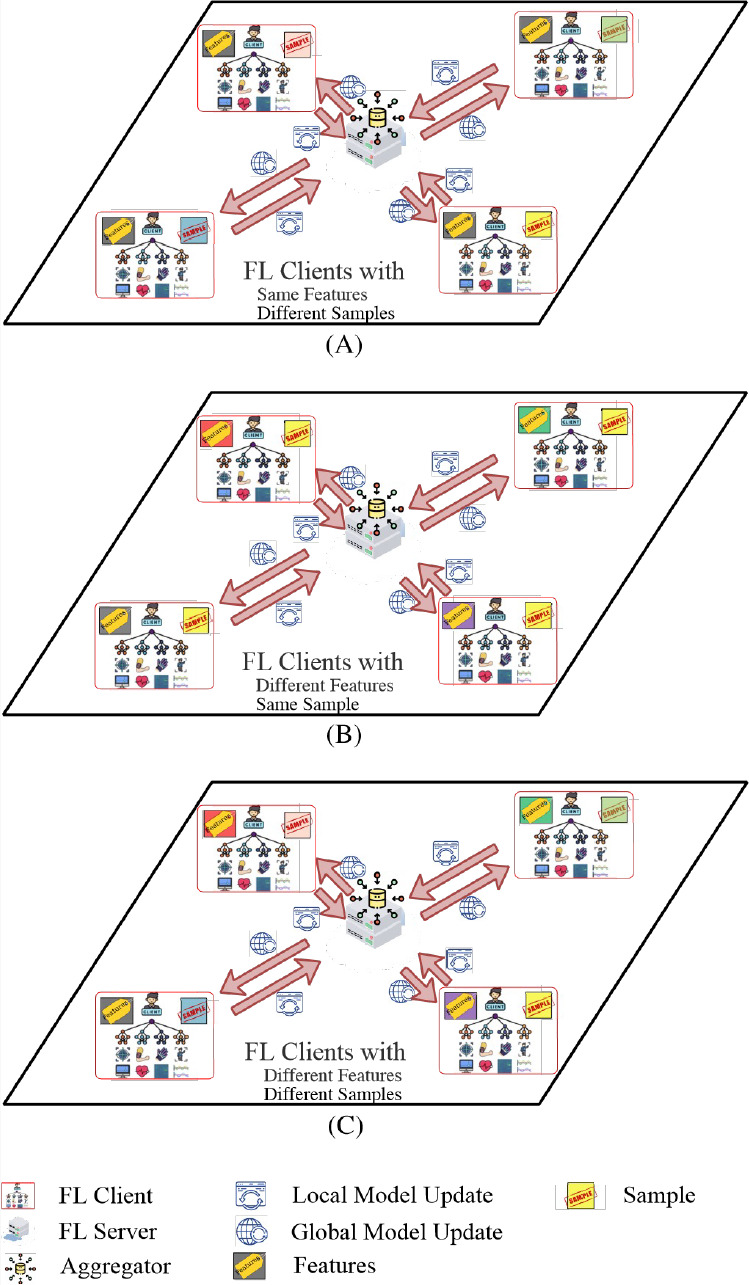
Types of federated learning (FL): (A) horizontal FL; (B) vertical FL; (C) federated transfer learning.

FL encompasses three prominent implementations tailored to diverse scenarios and constraints: (1) horizontal FL (HFL); (2) vertical FL (VFL); and (3) federated transfer learning (FTL) (Figure 6). The first implementation *HFL* involves training models across multiple devices or clients that possess similar data distributions (same features) but cannot share raw data due to privacy concerns. Each client trains a local model and shares only model updates (gradients/parameters) with a central server [240]. The server aggregates these updates to refine a global model, which is then redistributed. HFL is suitable where data are distributed with similar characteristics, such as mobile phones, IoT edge devices [241], and EEG data [242]. The challenges involve privacy-preserving techniques, communication efficiency, and aggregation strategies [243]. The second implementation *VFL* addresses scenarios where data are distributed across multiple parties with complementary features (different features) that cannot be shared due to privacy/proprietary concerns [244]. Each party holds a subset of features. The goal is collaborative model training without sharing raw data, commonly using secure multiparty computation (SMPC) and HE to enable computation on encrypted data while preserving privacy [245]. VFL is applied in health care where different institutions hold complementary patient data (eg, medical records and lab results) that are crucial for accurate models while preserving privacy [246]. The third implementation *FTL* extends traditional transfer learning to federated settings, where knowledge from related tasks or domains is leveraged across multiple decentralized datasets [247]. Unlike traditional transfer learning, FTL aggregates knowledge from decentralized clients to refine a base model (initialized from pretraining or scratch). This accommodates variations in data distributions and characteristics across clients [248]. FTL is beneficial where labeled data are scarce or unevenly distributed, such as electrocardiogram signal analysis [249], improving performance by leveraging knowledge from related domains.

#### Case Studies of FL in Health Care

FL holds promise in health care by facilitating the collaborative development of robust ML models across institutions (eg, hospitals and research centers) while safeguarding patient data privacy [250,251]. This approach trains ML models on local datasets, sharing only model updates (not raw data) to a central server for aggregation, thus mitigating privacy concerns and reducing data transfer costs [252]. FL offers enhanced privacy and utilization of diverse datasets without centralization. Real-world examples of FL in health care include the following: (1) *multi-institutional collaboration for disease diagnosis*, FL’s most significant success involves collaborative projects for disease diagnosis, and an international consortium (including institutions like Massachusetts General Hospital, University of Cambridge, Lahey Hospital, Assuta Medical Centers, Dasa S.A., National Taiwan University, and Seoul National University Hospital) successfully used FL to develop highly accurate models for predicting critical care patient outcomes (eg, mortality and length of stay) by leveraging diverse populations while maintaining data privacy; (2) *enhancing drug discovery and development*, FL has been used in pharmaceutical research to create predictive models for drug response and toxicity, and a notable instance is a project where multiple pharmaceutical companies shared algorithmic models, rather than data, to predict the success of drug compounds, expediting discovery while maintaining confidentiality of proprietary data [253]; and (3) *collaborative research*, FL enables institutions to collaborate on cancer research without sharing sensitive patient data, and a notable example is the collaboration facilitated by Intel and Penn Medicine, utilizing FL to identify brain tumors [254]. These cases demonstrate how FL advances medical research while maintaining data confidentiality.

#### Challenges of FL in Health Care

FL offers a groundbreaking opportunity to collaboratively develop potent ML models while safeguarding patient data privacy and minimizing transfer costs. However, realizing its full potential in health care requires overcoming significant challenges, including the following: (1) *data heterogeneity and model generalizability*, variability in medical data across institutions (different formats and diverse populations) complicates model convergence and performance, necessitating models that are generalizable to diverse patient populations and data types [255]; (2) *technical and computational limitations*, successful FL implementation requires substantial computational resources and technical expertise that may be unequally distributed among institutions, and balancing these disparities is crucial for success [256]; (3) *regulatory and ethical compliance*, FL must adhere to complex regulations like HIPAA (United States) and GDPR (Europe) governing data privacy and security, and ethical considerations require obtaining patient consent and ensuring equitable distribution of research benefits, necessitating clear data governance and ethical frameworks [257]; and (4) *scalability and real-time learning*, scaling FL models to accommodate real-time learning and large datasets poses technical hurdles, making the efficient management and real-time updating of models challenging [258].

Possible solutions include establishing standardized data formats across entities for consistency, implementing comprehensive data preprocessing pipelines to enhance quality, and designing FL algorithms adept at handling diverse data types and operating effectively across varying computational resources [259].

Future prospects involve integrating FL with emerging technologies like IoT devices and real-time health monitoring systems. This integration could enable the continuous improvement of ML models with real-time data from diverse, distributed sources, leading to more dynamic and responsive health care solutions. Additionally, advancements in edge computing could further enhance FL’s efficiency and scalability in health care [260].

### Current Trends and Future Directions of FL in Health Care

#### Overview

The landscape of FL in health care is rapidly evolving, driven by technological advancements and the growing need for collaborative and privacy-preserving data analysis. This section outlines the current trends shaping FL in health care and forecasts its future trajectory.

#### Current Trends of FL in Health Care

The prevailing trends shaping FL in health care reflect a dynamic evolution toward enhanced analytics, privacy, collaboration, and ethical considerations as follows: (1) *integration with advanced analytics and AI*, FL is being increasingly integrated with sophisticated AI techniques, such as deep learning, to enhance its analytical capabilities [261,262], and this allows for more complex and accurate models capable of addressing intricate health care challenges like personalized medicine and predictive analytics [250]; (2) *emphasis on data privacy and security*, due to heightened data privacy concerns, FL is gaining traction as a preferred method for collaborative health care research [263], and its inherent design, which allows for model training without sharing raw data, aligns well with stringent data privacy regulations like HIPAA and GDPR; (3) *cross-institutional collaborations*, there is a growing trend of cross-institutional collaborations, as explained previously, which is facilitated by FL, and these collaborations unite hospitals, research centers, and academic institutions, enabling them to pool knowledge and data resources for collective model improvement while maintaining data sovereignty; and (4) *ethical and fair AI development*, there is an increased focus on ethical AI development as FL evolves, ensuring that FL models are fair, unbiased, and representative of all patient demographics, thus addressing concerns around algorithmic bias [19,264,265].

#### Future Directions of FL in Health Care

FL in health care is poised for significant expansion, driven by emerging technological advancements and evolving health care landscapes. This forthcoming evolution encompasses the following: (1) *expansion into global health initiatives*, FL has the potential to significantly impact global health research, particularly in areas with stringent privacy laws or limited data-sharing capabilities, facilitating the analysis of global health trends and the development of models representative of diverse populations [250]; (2) *automated and dynamic model updating*, the future will likely see more automated and dynamic updating of FL models [266], enabling health care systems to respond quickly to new data or changing health trends, making the models more adaptive and responsive; (3) *use in remote and real-time monitoring*, with the proliferation of wearable devices and IoT in health care, FL is set to play a significant role in real-time patient monitoring and remote health care, providing personalized insights and treatments based on data from diverse patient populations [267]; (4) *edge computing integration*, integrating FL with edge computing could decentralize computational workload, allowing for faster and more efficient model training and updates, especially in real-time applications [265,268,269]; and (5) *integration with blockchain for enhanced security*, the integration of FL with blockchain technology is a promising development that bolsters data security and adds a layer of transparency and traceability to the FL process, ensuring immutable record-keeping and verifiable model updates in FL networks [13]. In summary, FL in health care is at a dynamic juncture, poised to reshape research and delivery. Its alignment with current needs for privacy, collaboration, and advanced analytics, coupled with its adaptability for future technological trends, positions FL as a key player in the future landscape of health care technology, paving the way for more equitable, secure, and efficient use of health care data globally.

## Blockchain

### Blockchain Background

Blockchain technology has undergone a remarkable evolution since its inception in 2009 with the creation of Bitcoin by an individual or group using the pseudonym Satoshi Nakamoto [270]. The primary purpose of Bitcoin was to establish a decentralized digital currency, and the innovation that made this possible was blockchain—a distributed ledger that records transactions across a network of computers securely and transparently.

In the following years, the potential applications of blockchain technology expanded beyond cryptocurrency. Vitalik Buterin introduced Ethereum [271] in 2015, introducing the concept of smart contracts—self-executing contracts with the terms of the agreement directly written into code. This development opened up a broader spectrum of decentralized applications (DApps) and laid the foundation for blockchain’s role in facilitating not only peer-to-peer (P2P) transactions but also complex programmable interactions.

The years that followed witnessed a surge in blockchain projects and platforms, each aiming to address specific challenges across various industries. The technology gained recognition for its potential to enhance transparency, security, and efficiency. Consortia and collaborations emerged, with enterprises exploring how blockchain could optimize supply chains [272-275], streamline financial transactions, and enhance data integrity.

Blockchain continues to evolve, with ongoing efforts to address scalability issues, energy consumption concerns, and regulatory considerations. From its humble beginnings as the underlying technology for Bitcoin, blockchain has grown into a versatile tool with the potential to reshape how industries manage and verify data. The technology’s journey reflects an ongoing quest for innovative solutions to long-standing challenges in the digital realm.

In this section, we provide a concise overview of fundamental concepts, features, structure, and taxonomy within the realm of blockchain technology.

### Blockchain Technology: An Overview

Blockchain technology is a decentralized and distributed ledger system designed to facilitate secure and transparent transactions without the need for a central authority. At its core, blockchain consists of a chain of blocks, each containing a list of transactions. These blocks are linked together in a chronological and immutable manner, forming a continuous chain. One of the key features of blockchain is its decentralization, meaning that the ledger is maintained by a network of nodes rather than a single central entity. This distributed nature enhances security, reduces the risk of fraud, and ensures transparency in the transaction process.

Blockchain technology possesses several distinctive features that contribute to its popularity and versatility across various industries. The features of blockchain technology are as follows: (1) decentralization, (2) immutability, (3) transparency, (4) security, (5) distributed ledger, (6) consensus mechanism, (7) anonymity and privacy, (8) efficiency and speed, (9) interoperability, and (10) ability to support smart contracts. First, regarding decentralization, blockchain operates on a P2P network, eliminating the need for a central authority or intermediary, and this enhances security, reduces the risk of a single point of failure, and promotes trust among mutually untrusted participants [276]. Second, immutability is the capability of a blockchain ledger to remain unchanged. Once a block is added to the blockchain, it becomes virtually impossible to alter or delete the information within it. Immutability ensures the integrity of the transaction history and builds trust in the accuracy of recorded data. Third, regarding transparency, the entire transaction history is visible to all participants in the network, and this fosters trust and accountability as participants can independently verify transactions and the state of the blockchain. Fourth, regarding security, blockchain uses cryptographic techniques to secure transactions and control access to the network. Consensus mechanisms, such as proof of work (PoW) and proof of stake (PoS), enhance security by preventing unauthorized changes to the blockchain [277-279]. Fifth, the ledger is distributed among the nodes over the network, and each node in the network holds a copy of the blockchain. This distribution ensures redundancy, resilience, and a shared source of trust among the participants. Sixth, consensus is a mechanism that gives the network the ability to agree upon the validity of transactions (and blocks) and the order in which they can be added to the blockchain. Seventh, regarding anonymity and privacy, while all transactions in the blockchain network are transparent, participants remain pseudonymous due to the use of public/private key pairs. Eighth, regarding efficiency and speed, blockchain reduces the need for intermediaries and manual processes, leading to faster and more efficient transactions. In some cases, however, the speed of transactions may depend on the specific consensus mechanism used. For instance, PoW blockchains, like Bitcoin, tend to be slower (ie, with less throughput) compared to traditional payment systems like Visa and Mastercard. The primary reasons for this include the inefficiency of the underlying consensus mechanisms and the way transactions are processed [280]. Ninth, blockchain interoperability refers to the capacity of various blockchain networks to interact seamlessly, facilitating the exchange of messages, data, and tokens among them [281-285]. Standards and protocols are evolving to enable such communication and data exchange between disparate blockchain platforms. The Inter-Blockchain Communication (IBC) protocol [286] is designed to facilitate this interoperability by providing a standardized way for independent blockchains to transfer and communicate with each other. Tenth, regarding the ability to support smart contracts, smart contracts are self-executing contracts with the terms directly written into code. These contracts automate and enforce predefined rules and agreements, reducing the need for intermediaries and streamlining processes [287,288].

### Consensus Mechanism in Blockchain

Consensus in the context of blockchain refers to the mechanism by which a distributed network of nodes agrees on the state of the system or the validity of transactions [289-291]. Since blockchain operates in a decentralized and trustless environment, consensus is crucial to ensure that all participants have a consistent view of the blockchain’s history and current state. The consensus mechanism is responsible for preventing double-spending (where the same digital asset is spent more than once) and maintaining the integrity of the blockchain. Different blockchain networks use various consensus algorithms, each with its own set of rules and processes. The most prominent and currently existing consensus protocols are as follows: PoW; PoS; Byzantine fault tolerance (BFT); direct acyclic graph tangle (DAG); and hybrid consensus, including PoS and PoW hybrid, proof of authority (PoA) and PoW hybrid, delegated PoS (DPoS) and PoA hybrid, PoW and BFT hybrid, and hybrid voting system.

#### PoW Protocol

This is the original consensus algorithm used by Bitcoin [270] and many more cryptocurrencies. In PoW, participants (miners) solve complex mathematical puzzles to validate transactions and create new blocks. The first miner to solve the puzzle gets the right to add a new block to the blockchain. PoW is resource-intensive and requires a significant amount of computational power [292]. Blockchains that rely on PoW are more prone to forks. A fork in blockchain technology refers to a split in the blockchain’s transaction history, resulting in two or more separate paths. This can occur for various reasons, such as changes in protocol rules, disagreements among participants, or software upgrades [293].

#### PoS Protocol

In PoS [294], validators (ie, block proposer participants) are chosen to create new blocks and validate transactions based on the amount of cryptocurrency they hold and are willing to “stake” as collateral. This eliminates the need for energy-intensive mining and aims to provide a more energy-efficient alternative to PoW. Examples include Ethereum’s [295] transition to Ethereum 2.0, Cardano [296], and Algorand [297]. DPoS is an improvement over traditional PoS in terms of scalability and efficiency [298].

#### BFT Protocols

BFT consensus algorithms are a class of protocols designed to achieve consensus in distributed systems, even in the presence of faulty or malicious nodes. In a BFT-based consensus algorithm, a network of nodes collaborates to agree on the state of the system or the validity of transactions. The term “Byzantine fault” originates from the Byzantine generals’ problem [299], a theoretical scenario where a group of generals must come to a unanimous agreement on a coordinated action, despite the possibility of some generals being traitors and sending conflicting messages. Practical BFT (pBFT) is the most prominent variant of BFT-based consensus protocols [300]. This algorithm is designed to tolerate up to one-third of the total number of nodes being faulty or malicious. This means that as long as no more than one-third of the nodes in the network exhibit Byzantine behavior (ie, they may fail arbitrarily or behave maliciously), pBFT can still reach consensus and continue to operate correctly. Some variants of BFT (eg, Hotstuff [301], pBFT, and improved BFT) are supported by Hyperledger Fabric [302].

#### DAG Protocols

DAG consensus protocols are a class of distributed consensus algorithms that use a data structure called a directed acyclic graph to achieve agreement on the order of transactions or events in a decentralized network. Unlike traditional blockchain-based consensus protocols where transactions are organized into linear blocks, DAG-based protocols organize transactions in a more flexible graph structure [303]. One of the most well-known implementations of DAG consensus is the tangle [304], which is used in the IOTA cryptocurrency network [305]. In the tangle, each transaction directly references and approves 2 previous transactions, forming a directed acyclic graph structure. The most prominent blockchains that run on proof of capacity include Signum, Chia, and SpaceMint.

#### Hybrid Consensus Protocols

##### Overview

Hybrid consensus models in blockchain combine elements of multiple traditional consensus mechanisms to leverage their respective strengths and mitigate their weaknesses. These models aim to achieve a balance among decentralization, security, scalability, and energy efficiency [306]. Some hybrid consensus models in blockchain are described below.

##### PoS and PoW Hybrid

Some blockchain networks combine PoS and PoW mechanisms to achieve consensus (eg, TwinsCoin [307]). For example, a PoW component may be used for initial block creation, while PoS is utilized for subsequent block validation or as a way to elect validators. This hybrid approach aims to maintain security through PoW while improving scalability and energy efficiency with PoS.

##### PoA and PoW Hybrid

In this hybrid model, a network may utilize PoW for initial block creation and PoA for block validation. PoW ensures the initial distribution of tokens and secures the network against Sybil attacks, while PoA provides fast finality and scalability by relying on known and trusted validators.

##### DPoS and PoA Hybrid

DPoS allows token holders to vote for a limited number of delegates who are responsible for block validation. In a hybrid approach, DPoS can be combined with PoA, where the initial set of validators is determined through PoA, and then, token holders can vote for additional delegates using DPoS. This hybrid model aims to achieve both decentralization and scalability.

##### PoW and BFT Hybrid

This hybrid model combines the energy-intensive PoW with a BFT-based consensus algorithm such as pBFT or Tendermint [308]. PoW is used for block creation, while BFT consensus ensures finality and fault tolerance. This approach aims to achieve both security and efficiency in blockchain networks [309].

##### Hybrid Voting Systems

Some blockchain networks combine different voting mechanisms, such as direct voting by token holders and voting by elected delegates [310]. This hybrid voting system aims to balance the influence of token holders with the expertise and accountability of elected representatives.

### Blockchain Data Structure

In blockchain technology, the data structure plays a pivotal role in ensuring the integrity, security, and immutability of the distributed ledger [278,311]. At its core, a blockchain is composed of a series of blocks, each containing a bundle of transactions. These blocks are cryptographically linked together sequentially, forming a continuous chain. The data structure of a block typically includes several key components: a header, a list of transactions, and a cryptographic hash. The header contains metadata, such as the block’s unique identifier (block hash), a timestamp, and a reference to the previous block’s hash, thus establishing the chronological order of blocks. The transaction list records the details of all transactions included in the block, such as sender and receiver addresses, transaction amounts, and cryptographic signatures for verification. Additionally, each block is assigned a cryptographic hash, which is computed based on its contents using a hashing algorithm like SHA-256. This hash serves as a unique identifier for the block and is crucial for maintaining the integrity of the blockchain. Any alteration to the data within a block would result in a change in its hash, thereby breaking the chain’s continuity and signaling tampering. The inherent immutability and tamper-resistance of the data structure in blockchain ensure that once recorded, transactions cannot be altered or deleted without consensus from the network participants, establishing a reliable and transparent system for recording and verifying transactions [312].

### Blockchain Network and Architecture

The network architecture in blockchain is a distributed and decentralized system that enables the secure and transparent exchange of data and value across a network of interconnected nodes. At its core, a blockchain operates as a P2P network where each participant, or node, maintains a copy of the entire blockchain ledger. This distributed architecture ensures that there is no single point of failure, as the data are replicated and synchronized across multiple nodes. Nodes communicate with each other through a consensus mechanism. Depending on the consensus algorithm used, nodes may take on different roles, such as miners in the PoW system or validators in the PoS system. Transactions are broadcast to the network and validated by consensus, typically requiring confirmation from a majority of nodes before being added to the blockchain. This network architecture provides several benefits, including resilience against censorship and tampering, increased transparency and accountability, and enhanced security through cryptographic techniques.

Additionally, the decentralized nature of blockchain networks promotes trust among participants by eliminating the need for intermediaries and central authorities, thereby fostering a more inclusive and democratic ecosystem for conducting transactions and exchanging value [313].

**Figure 7.**
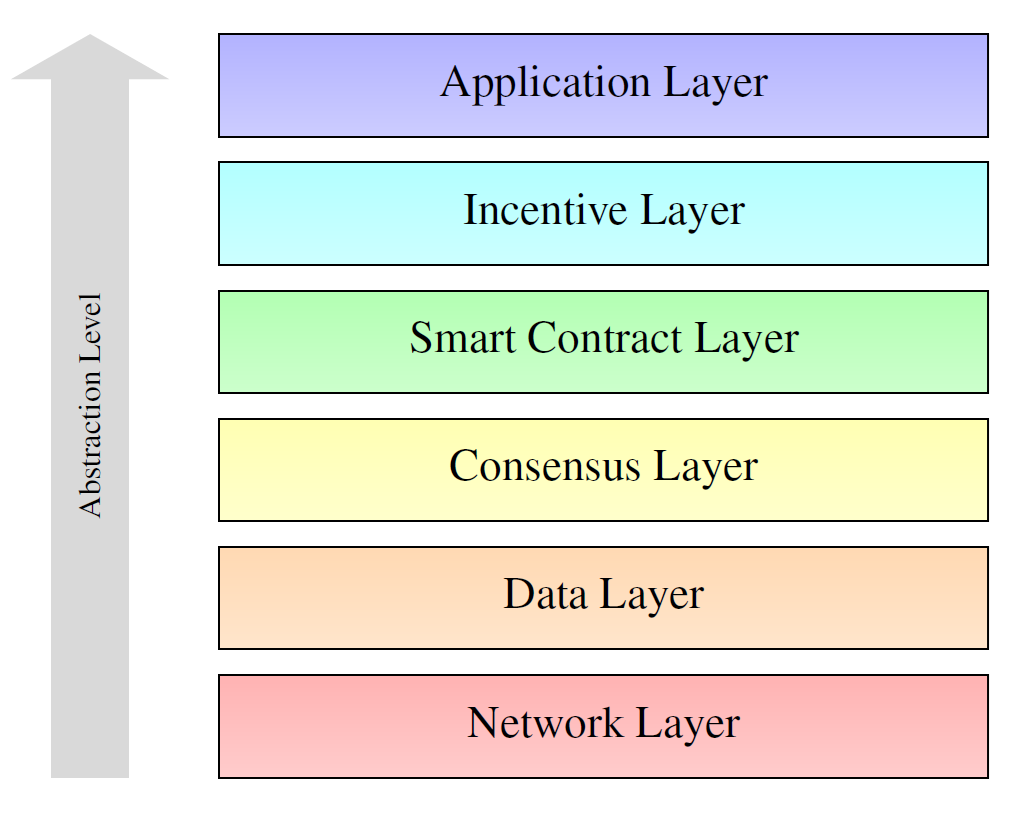
Six-layer blockchain architecture illustrating increasing abstraction from the network layer to the application layer.

Blockchain technology typically consists of 6 common layers, each serving a specific purpose in the network’s function and security (Figure 7). The six layers are as follows: (1) *network layer,* the foundation facilitating communication between nodes using protocols like TCP/IP, HTTP, and P2P protocols, responsible for transmitting data (transactions and blocks) across the network; (2) *data layer*, stores the actual blockchain data, including blocks, transactions, and smart contracts, utilizing structures and storage mechanisms optimized for secure and efficient retrieval; (3) *consensus layer*, ensures all nodes agree on the validity and order of transactions added to the blockchain using various mechanisms, such as PoW, PoS, DPoS, and pBFT; (4) *smart contract layer*, enables the creation and execution of programmable, self-executing contracts with terms written directly into code, powering various DApps; (5) *incentive layer*, provides mechanisms (typically block rewards and transaction fees, eg, Bitcoin’s block rewards and Ethereum’s gas fees) to incentivize participants (miners or validators) to contribute resources and maintain the network’s security and integrity; and (6) *application layer*, encompasses the user-facing applications and interfaces (eg, DApps, wallets, smart contracts, and other software) that interact with the blockchain protocol.

### IBC Protocol

Interoperability is one of the most important features of next-generation blockchain networks and refers to the ability of different blockchain platforms to communicate, share data, and transact with each other seamlessly. It enables interoperability between disparate blockchain networks, allowing them to interact and exchange information or assets without the need for intermediaries or centralized exchanges. Interoperability is essential for realizing the full potential of blockchain technology by facilitating cross-chain transactions, asset transfers, and data sharing between different blockchain ecosystems [286].

The IBC protocol is a set of standards and protocols designed to enable communication and interoperability between independent blockchain networks. IBC facilitates the secure and trustless transfer of assets and data across different blockchains, allowing them to interact and transact with each other directly. The protocol defines a standardized messaging format and a set of rules for validating and verifying transactions between participating blockchains. By implementing the IBC protocol, blockchain networks can establish interconnectivity, enabling cross-chain transactions, decentralized exchanges, and interoperable DApps [283,314,315].

IBC is a pillar of the Internet of Blockchains [311,316], a vision where blockchain networks are interconnected like the global internet, creating a decentralized and open ecosystem where data, assets, and services flow freely between different blockchains. Examples of interoperability solutions include the following: (1) *Cosmos,* a decentralized network utilizing the IBC protocol to enable communication and interoperability between its various blockchain platforms, with the Cosmos Hub serving as the primary connection point [317]; (2) *Polkadot,* a multichain blockchain platform that enables interoperability between different parachains (parallel blockchains) within its network via its relay chain, allowing them to share data and assets seamlessly [318]; and (3) *Wanchain,* a cross-chain blockchain platform focused on interoperability, enabling the secure and decentralized exchange of assets between different blockchain networks, including Bitcoin and Ethereum [319].

### Blockchain Taxonomy

#### Overview

At a high level, blockchain networks are classified into 3 main categories: private, public, and consortium blockchains. These are briefly explained in the following subsections.

#### Private Blockchain

A private blockchain is a permissioned blockchain network where access and participation are restricted to authorized entities only. These entities typically have known identities and are granted permission to join the network by a central authority or administrator. Private blockchains are often used by enterprises and organizations to build internal blockchain solutions for specific use cases such as supply chain management, document verification, and intercompany transactions. They offer enhanced privacy, control, and scalability compared to public blockchains [320,321]. For instance, Hyperledger Fabric is a private blockchain framework developed by the Linux Foundation’s Hyperledger project [302,322,323]. It is designed for enterprise use cases and enables organizations to create permissioned blockchain networks with customizable features and governance models.

#### Public Blockchain

A public blockchain is a permissionless blockchain network that is open to anyone to join, participate, and transact without requiring permission or identification. Public blockchains are decentralized networks where transactions are transparent, immutable, and verifiable by anyone. They offer high levels of transparency, censorship resistance, and security but may sacrifice scalability and privacy due to their open nature [324-327]. Public blockchains are often used for cryptocurrencies, DApps, and tokenized assets. For instance, Bitcoin operates as a decentralized P2P network for sending and receiving the Bitcoin cryptocurrency.

#### Consortium Blockchain

A consortium blockchain is a semidecentralized blockchain network governed by a consortium or group of organizations rather than a single centralized entity. Consortium blockchains are permissioned networks where the consensus process and governance are shared among a predefined set of participants. Consortium blockchains are commonly used in industries or sectors where multiple organizations collaborate on shared processes or infrastructure while still maintaining some level of control and privacy [328-331]. They offer a balance between the decentralization of public blockchains and the control of private blockchains. For instance, R3 Corda is a consortium blockchain platform developed by the enterprise blockchain consortium R3. Corda is designed for use cases that require privacy, scalability, and interoperability in multiple organizations from sectors such as finance, health care, and supply chain.

## Integration of FL With Blockchain

### Overview

Integrating blockchain technology with FL has emerged as a novel approach to address inherent data privacy, security, and trust challenges within distributed ML systems [21,23,332,333]. FL, characterized by training ML models across decentralized devices without centrally aggregating raw data, offers significant advantages in preserving user privacy and data confidentiality. In other words, in federated ML, the exchange occurs at the parameter level instead of transmitting raw data. This approach mitigates the risk associated with centralized computing architectures, which are susceptible to targeted attacks and potential denial of service due to their single-point-of-failure vulnerability. A detailed discussion on FL is presented in the FL Details section, including its different types (HFL, VFL, and FTL), applications (particularly in health care), and potential future directions (integration with advanced analytics and cross-institutional collaborations), highlighting FL’s pivotal role in reshaping health care technology.

However, concerns persist regarding the integrity of FL systems, particularly regarding data tampering or manipulation by malicious or compromised nodes. Blockchain, renowned for its immutable and transparent ledger capabilities, presents a compelling solution to these challenges. By leveraging blockchain’s decentralized consensus mechanisms and cryptographic primitives, FL systems can ensure data integrity, traceability, and transparency throughout the ML model training process [334-336]. Moreover, blockchain’s smart contract functionality enables the establishment of auditable and self-executing agreements among participants, further enhancing the trustworthiness of FL collaborations [23]. This integration not only addresses privacy and security concerns but also fosters a more collaborative and inclusive environment for distributed ML research and applications. As such, the motivation behind the integration of blockchain and FL lies in the pursuit of enhancing data privacy, security, and trust in decentralized ML ecosystems, ultimately advancing the adoption and efficacy of FL methodologies in various domains such as health care [13,336,337].

Blockchain technology holds immense potential to significantly enhance the security, transparency, and trustworthiness of FL systems by addressing key challenges like verifying local model updates, aggregating the global model, and incentivizing participants. The integration of blockchain into FL is explored as follows: (1) verifying local model updates, (2) global model aggregation, and (3) incentivizing participants. First, regarding verifying local model updates, blockchain offers an immutable and tamper-proof record for local model updates from participants. These updates are recorded as transactions, ensuring their validity and preventing unauthorized modifications. Furthermore, smart contracts can be used to define specific rules, and they function as verification mechanisms, executing algorithms to validate the integrity and accuracy of updates [338]. Second, regarding global model aggregation, blockchain empowers FL with decentralized consensus mechanisms (eg, PoW and PoS) that allow participants to collectively agree on the process of aggregating the global model. The entire aggregation process is transparently recorded on the blockchain, allowing participants to verify the fairness and accuracy of the final model [339]. Third, regarding incentivizing participants, a key strength of blockchain is its ability to create tokenized incentives (tokens or cryptocurrencies) to reward participants who contribute data or computational resources [340]. Smart contracts automate the distribution of these incentives based on predefined criteria (eg, quality of contribution and computational resources provided) [341]. Crucially, the entire distribution process is transparently recorded on the blockchain, ensuring accountability and fairness [342].

By leveraging the unique strengths of blockchain technology, FL systems can achieve a new level of security, transparency, and trust, ultimately fostering a more collaborative and efficient environment for AI development.

### Integration Architecture

Several research efforts have identified distinct architectures for integrating blockchain and FL. This paper proposes a framework similar to that in a previous report [343], with slight variations in terminology. Based on the level of interaction between blockchain and FL entities, we categorize these architectures as fully coupled, semicoupled, and loosely coupled.

#### Fully-Coupled Architecture

**Figure 8.**
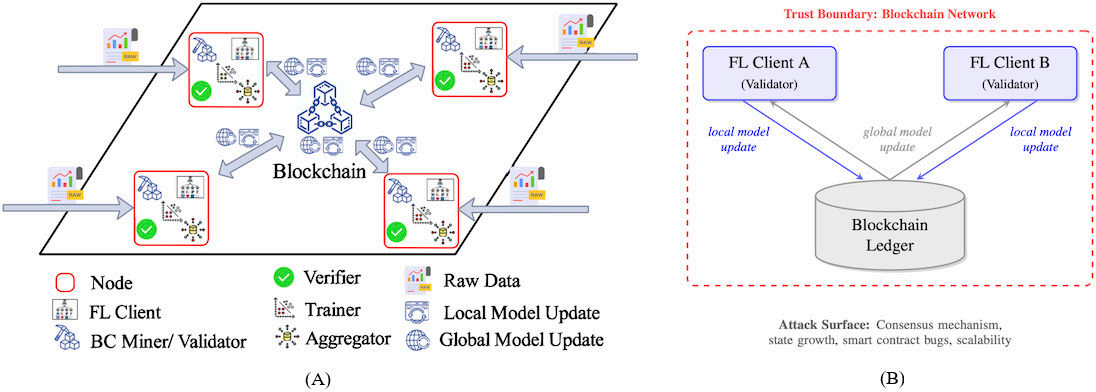
Fully-coupled blockchain-based federated learning (FL) architecture: (A) detailed architecture; (B) schematic diagram. All local updates are committed on-chain via a smart contract that performs aggregation. Clients act as validators within a unified blockchain trust domain.

In the fully-coupled architecture, blockchain nodes (ie, miners or validators) perform dual roles as FL clients. Within this architecture, FL clients engage in computing local model updates as well as validating these updates as blockchain nodes. Notably, blockchain nodes not only partake in training local models but also participate as the global model aggregator. The aggregator, which may be a selected node, a designated leader, or a collection of nodes based on the predefined protocol, is responsible for gathering local model updates. Every node in this model has the opportunity to function as a blockchain validator, a local model trainer, and a global aggregator concurrently. Consequently, both local model updates and global model updates are contained within the blockchain. Importantly, the absence of a necessity to transmit the global model to a central server mitigates the risk of a single point of failure within this architecture. A schematic of this architecture is depicted in Figure 8.

#### Semicoupled Architecture

**Figure 9.**
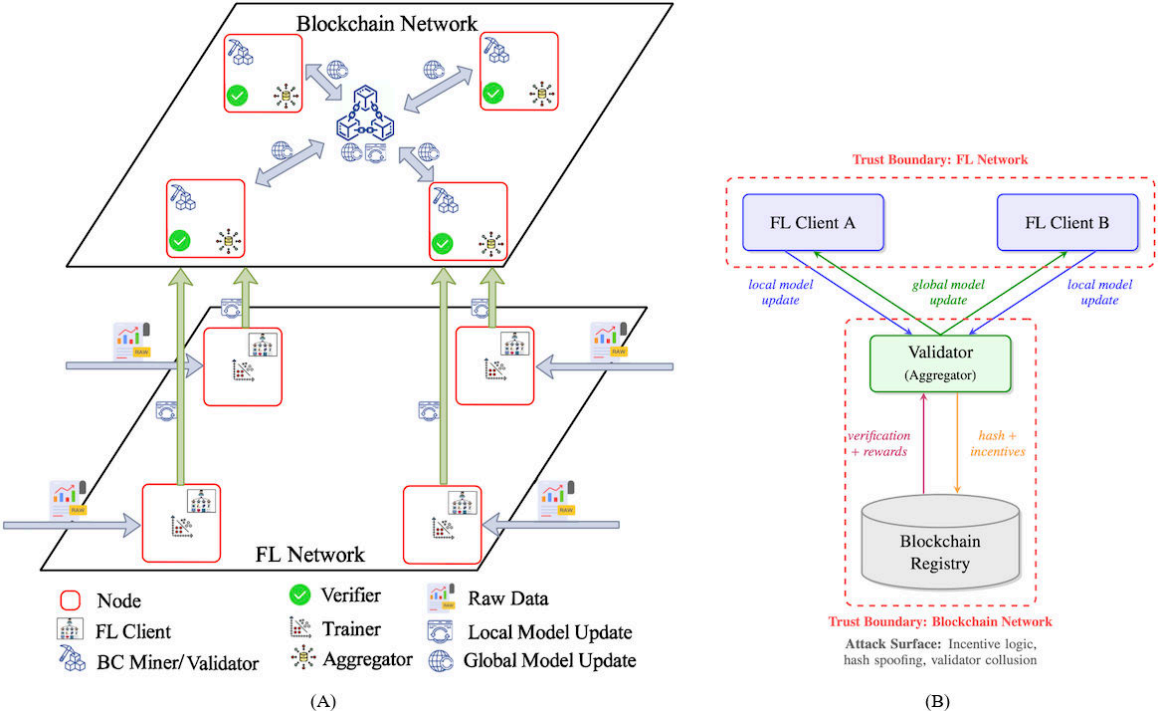
Semicoupled blockchain-based federated learning (FL) architecture: (A) detailed architecture; (B) schematic diagram. Validators perform aggregation off-chain with an on-chain registry and incentives.

In the semicoupled architecture, blockchain and FL clients inhabit separate networks, although FL clients retain the capability to interact with the blockchain and manipulate the distributed ledger. FL clients gather data from diverse sources, train local models, and subsequently upload local model updates to the blockchain. Blockchain nodes (ie, miners or validators) are tasked to validate the uploaded local model updates that will be used for training the global model. Upon the preparation of the global model, blockchain nodes will store it within the blockchain. Participant rewards are allocated based on a predefined incentive mechanism. This architecture also circumvents the potential for a single point of failure. A schematic of this architecture is depicted in Figure 9.

#### Loosely-Coupled Architecture

**Figure 10.**
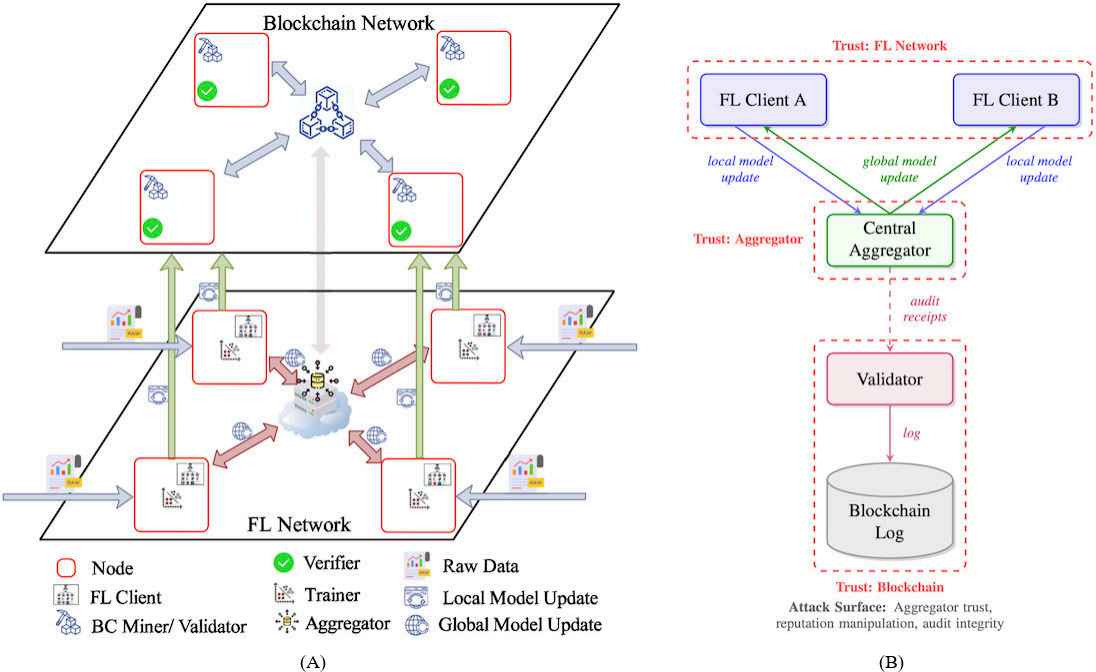
Loosely-coupled blockchain-based federated learning (FL) architecture: (A) detailed architecture; (B) schematic diagram. Central aggregator with blockchain used only for audit logs.

In the loosely-coupled architecture, blockchain nodes and FL clients are in 2 distinct networks. This architecture introduces reputation as a criterion for measuring the reliability of the clients. In this architecture, the primary function of the blockchain is to furnish a coordination mechanism for clients, manage their reputation, handle authentication, manage validation of local model updates, and manage incentives (ie, contributions are managed to ascertain reputation and incentivize participation). While the blockchain validates local model updates, it refrains from storing them. In contrast, it stores data related to the reputation of the participants. The responsibility of the FL clients is to train local models and upload updates to the blockchain for validation. After validation, these updates are sent to an aggregator, which can be a distinct server or a cloud space. A schematic of this architecture is depicted in Figure 10.

#### Cross-Architecture Synthesis and Quantitative Cost Analysis

The 3 BCFL integration patterns examined above represent distinct points on a design spectrum trading decentralization for performance and operational simplicity. The fully-coupled design maximizes auditability by executing all operations on-chain, but incurs significant latency (bounded by the blockchain finality time *t_f_*) and ledger state growth, limiting scalability to small consortia (≤50 clients). The semicoupled design achieves a pragmatic balance by performing model aggregation off-chain while recording verifiable provenance and incentive information on-chain, supporting medium-scale health care deployments (≤100 clients). The loosely-coupled design minimizes blockchain interaction, using it primarily for coordination and audit, offering high throughput for large IoMT or regional networks (≥200 clients). Practitioners can map deployment size, regulatory constraints, and trust requirements to these design points to select an optimal integration pattern, as summarized in Table 2.

**Table 2.** Comparative characteristics and quantitative cost trends across blockchain-based federated learning integration architectures.^a^

Characteristic	Fully-coupled architecture	Semicoupled architecture	Loosely-coupled architecture
Trust model	Fully decentralized (all clients act as validators)	Partially decentralized (off-chain aggregation, on-chain proofs)	Federated trust (central aggregator with optional audit)
Latency per round	High (∼*t_f_*^b^; consensus limited)	Medium (off-chain aggregation, on-chain logging)	Low (minimal blockchain interaction)
Throughput/scale	≤50 clients	≤100 clients	≥200 clients
Compute overhead ∆_BC_	12%-15% (cryptographic + consensus tasks)	5%-8% (hashing + verification)	2%-4% (logging + coordination)
Network cost pattern	Full model updates transmitted on-chain	Partial metadata and incentive records	Receipts and coordination only
On-chain storage/round	*C*^c^*q*^d^*|W|*^e^ (hundreds of MBs)	10-100 kB	*C*×0.3 to 1 kB
Auditability	Very high (complete lifecycle recorded)	High (key model lineage and incentives)	Moderate (hash receipts only)
Best use	High-security, small-scale consortia	Balanced health care networks	Large-scale IoMT^f^ or regional systems

a Typical health care configuration: clients per round (*C*) = 50, model size (|*W*|) = 80 MB (FP32), compression ratio (*q*) = 0.1, global rounds (*R*) = 200, finality time (*t*_*f*_) = 1 to 10 s, client churn rate (*λ*) ≈ 0.05.

b *t*_*f*_: finality time.

c *C*: clients per round.

d *q*: compression ratio.

e |*W*|: model size*.*

f IoMT: Internet of Medical Things.

The quantitative cost model formalizes these tradeoffs. The per-client computational load combines local training and blockchain overhead as follows: FLOPs_client_ ≈ *E n f* (1 + ∆_BC_), where *E* represents local training epochs per round, *n* represents samples in the local dataset, *f* represents floating-point operations per sample, and ∆_BC_ represents blockchain overhead from cryptographic signing, consensus participation, and data serialization. Empirical studies report ∆_BC_ values of approximately 2%-4% for the loosely-coupled design, 5%-8% for the semicoupled design, and 12%-15% for the fully-coupled design [339,344].

Network usage grows linearly with communication rounds as follows: Bytes_client_ ≈ 2*q* |*W*| *R*(1 + λ), where |*W*| represents model parameter size, *q* represents the compression ratio (0<*q*≤1), *R* represents total rounds, and λ represents the client churn rate. The factor of 2 accounts for upload and download traffic. Storage requirements differ sharply for the loosely-coupled design (*C*×0.3 to 1 kB/round), semicoupled design (10 to 100 kB/round), and fully-coupled design (*Cq*|*W*|/round). For typical health care configurations, fully-coupled deployments require approximately 400 MB on-chain per round and approximately 3.2 GB total transfer per client, which are considered impractical for most BFT blockchains [302,345], whereas semicoupled and loosely-coupled designs reduce bandwidth by 40%-50% with a submegabyte on-chain state. Round latency is lower-bounded by finality time *t_f_*, which increases with validator set size *N_v_* due to *O*(*N_v_*^2^) message complexity of the BFT consensus [345,346]. Consequently, the fully-coupled design suits small, high-security networks, while the loosely-coupled design offers practical scalability for large federations.

#### Assumptions and Scope

All values are order-of-magnitude estimates derived under synchronous federated averaging [347] and BFT blockchain configurations (eg, Hyperledger Fabric [302] and Tendermint [348]). While real-world performance may vary with model architecture, bandwidth, or consensus protocol, the relative scaling behavior and architectural tradeoffs among computation, communication, and on-chain costs remain consistent across implementations, including asynchronous or adaptive variants.

### Security and Privacy Considerations

#### Overview

Integrating FL and blockchain addresses security and privacy challenges inherent in collaborative health care learning. While FL enhances privacy by training models on local datasets, it introduces vulnerabilities like model poisoning, gradient leakage (potential for patient reidentification), and coordination failures [349].

Blockchain mitigates these risks via its immutable, decentralized framework, strengthening FL through the following: (1) *immutable audit trails*, every model update, contribution, and access event is cryptographically recorded and timestamped on the blockchain, creating a verifiable and transparent history for forensic investigation [350]; (2) *decentralized trust and consensus*, blockchain eliminates reliance on a central authority, distributing trust across nodes to enhance resilience against single points of failure; and (3) *cryptographic security*, mechanisms ensure data integrity, authenticity, and nonrepudiation for all transactions and model updates, reinforcing the overall trustworthiness.

To further enhance privacy and compliance in BCFL systems, the following advanced cryptographic and security techniques are used: (1) ZKPs, (2) HE, (3) SMPC, (4) trusted execution environments (TEEs), and (5) DP. First, ZKPs enable participants to prove compliance (eg, data quality and consent) without revealing underlying sensitive patient data, allowing verifiable protocol adherence while safeguarding privacy [351,352]. However, ZKPs face substantial computational challenges, limiting practical applicability in real-time clinical systems [353,354]. Second, HE permits computation on encrypted data, enabling secure aggregation of model updates [352,355]. Although improved, HE introduces computational overhead (eg, 3.7 times to 8.2 times in health datasets) that presents scalability concerns and requires specialized hardware in resource-constrained settings [356]. Third, SMPC enables joint computation of aggregate functions (eg, model averaging) over private inputs without revealing individual contributions, offering strong privacy during aggregation [357,358]. SMPC protocols introduce significant computational and communication overhead through intensive cryptographic operations, posing a challenge in bandwidth-constrained health care networks [359,360]. Fourth, regarding TEEs, hardware-based secure enclaves provide isolated computation for sensitive operations (eg, aggregation) with cryptographic attestation [361,362]. However, TEEs suffer from memory limitations necessitating layer-wise training (performance overhead) and remain vulnerable to side-channel attacks (eg, cache timing and speculative execution) that can compromise confidentiality [363,364]. Fifth, DP introduces controlled noise to model updates or gradients for quantifiable privacy guarantees [365]. DP introduces critical privacy-utility tradeoffs (eg, 1%‐3% accuracy reduction in COVID-19 models), and the required privacy budget calibration is challenging, especially in non-independent and identically distributed health care data settings where noise and heterogeneity can slow model convergence [366,367].

#### Threat Model for BCFL in Health Care

We define a comprehensive threat model for BCFL systems in health care, identifying adversaries, capabilities, attack objectives, and mitigation strategies. This model provides a formal foundation for understanding security guarantees and tradeoffs, enabling practitioners to select appropriate defenses for specific deployment scenarios.

#### Adversaries and Capabilities

The BCFL ecosystem faces four adversary classes as follows: (1) *malicious clients*, these are compromised health care institutions or IoMT devices executing data poisoning, model poisoning [6,368], free-riding, or inference attacks with full access to local training processes; (2) *malicious aggregators*, these are present in loosely-coupled or semicoupled architectures, and they may infer private data from updates, bias global models through selective exclusion, or manipulate aggregation; (3) *Byzantine validators*, they comprise colluding blockchain nodes approving invalid updates, double-spending incentive tokens, or disrupting consensus; and (4) *external adversaries*, they include passive eavesdroppers intercepting communications, performing traffic analysis, or executing membership inference attacks.

#### Attack Objectives

Adversaries pursue distinct objectives through various attacks as follows: (1) *integrity attacks*, corrupt model reliability through model poisoning [6,368,369], backdoor insertion [370,371], and Sybil attacks, potentially causing misdiagnosis; (2) *privacy attacks*, extract patient data via gradient inversion [372-374], membership inference [375,376], and model inversion, violating HIPAA and GDPR guidelines; (3) *availability attacks*, disrupt services through denial-of-service on FL coordination, blockchain consensus, or participants, impacting clinical decision support; and (4) *fairness attacks*, introduce demographic biases or exclude underrepresented populations [377], exacerbating health care disparities.

#### Threat Mitigation

Table 3 maps threats to mitigation techniques with quantified performance implications from the Empirical Validation and Performance Evaluation section. These mitigations provide complementary protection layers deployable based on application requirements. Byzantine-robust aggregation [368,378] maintains integrity with up to 30% compromised participants but incurs 3%-5% accuracy degradation. HE provides semantic security with 3.7 to 8.2 times computational overhead [379]. DP [380] offers formal (ϵ,δ)-privacy guarantees with 1%-3% accuracy reduction, as observed in COVID-19 models.

**Table 3.** Threat mitigation mapping for blockchain-based federated learning in health care.

Threat	Mitigation	Security guarantee	Cost
Model poisoning	Byzantine-robust aggregation (Krum, FLTrust) [368]; Reputation systems	Resilient to ≤30% malicious clients	3%-5% accuracy drop
Gradient leakage	HE^a^ [352]; SMPC^b^ [379]	Semantic security for updates	HE: 3.7 to 8.2 times overhead
Membership inference	DP^c^ [380,381]; Gradient masking	(ϵ,δ)-privacy	1%-3% accuracy loss
Free-riding	Proof of federated work [382,383]; Tokenized incentives	Verifiable contribution	On-chain verification overhead
Model inversion	ZKPs^d^ [351]; TEEs^e^	Verifiable computation	ZKPs: 8-15 s proof time
Consensus attacks	BFT^f^ protocols (PBFT^g^, IBFT^h^) [300,345]	Tolerates less than one-third Byzantine nodes	*O*(*N*^2^)^i^ message complexity

a HE: homomorphic encryption.

b SMPC: secure multiparty computation.

c DP: differential privacy.

d ZKPs: zero-knowledge proofs.

e TEEs: trusted execution environments.

f BFT: Byzantine fault tolerance.

g PBFT: practical Byzantine fault tolerance.

h IBFT: improved Byzantine fault tolerance.

i *O*(*N*2): quadratic time complexity.

#### Architectural Security Tradeoffs

The 3 BCFL architectures provide distinct security-performance tradeoffs. Fully-coupled architectures maximize security through on-chain execution but incur approximately *t_f_* latency per round and 12%-15% overhead, and they are suitable for high-security consortia with stringent audit requirements. Semicoupled architectures balance security and performance through selective on-chain logging, maintaining strong auditability with 5%-8% overhead and supporting up to 100 participants. Loosely-coupled architectures optimize scalability with minimal blockchain interaction, and they are suitable for large-scale IoMT deployments but require stronger trust in the central aggregator.

Empirical validation shows that these layered mitigations maintain accuracy within 2%-3% of nonprivate baselines. Combining DP (*ϵ*<0.1) with encrypted updates sustains utility, with an approximately 2.8 times compute overhead, while Byzantine robust aggregation maintains less than 5% degradation with up to 30% compromised clients. Mitigation selection should be guided by regulatory compliance (HIPAA/GDPR), data sensitivity, and clinical impact on patient outcomes.

#### Practical Deployment Challenges

The integration of privacy-enhancing technologies into health care involves technical intricacies regarding compatibility, efficiency, and communication overhead [12]. FL requires uniform infrastructure and capabilities across all sites, and unreliable infrastructure can disrupt training [384]. Cross-institutional coordination is amplified by complex data governance, regulatory requirements (eg, HIPAA and GDPR), and heterogeneous resource constraints [344]. Methodological flaws (eg, privacy issues, generalization, and communication costs) have rendered most recent FL health care studies inappropriate for clinical use [385].

Integration architecture significantly impacts tradeoffs as follows: (1) the fully-coupled architecture provides maximum auditability by logging all FL operations on-chain (highest security) but faces scalability challenges due to blockchain throughput limitations and consensus inefficiency, particularly with traditional PoW or basic PoS [344,386]; (2) the semicoupled architecture balances security and performance by selectively recording critical events (eg, model updates) on-chain while keeping routine operations off-chain; and (3) the loosely-coupled architecture offers the greatest scalability by using blockchain primarily for coordination and incentives, relying on off-chain security for the learning process, but it requires additional trust assumptions. Emerging architectures utilize sidechains to address scalability by enabling parallel processing and faster verification [386]. Addressing implementation challenges requires continued advancement in specialized hardware acceleration (eg, DARPA’s FHE-focused DPRIVE project [387]), adaptive privacy mechanisms, and standardized protocols for cross-institutional deployments.

#### Operational Resilience and Failure Modes

Beyond cryptographic security, BCFL operational resilience requires predefined failure protocols with consortium-approved, measurable triggers. We define the following policy parameters: τ_quorum_ (minimum client quorum), Θ_round_ (round deadline), τ_byz_ (maximum tolerated Byzantine clients for robust aggregation [388]), δ_AUROC_ (maximum allowed area under the receiver operating characteristic curve drop), δ_ECE_ (maximum allowed expected calibration error increase), τ^major^_PSI_ (population stability index bound for major drift), τ_txfail_ (transaction failure rate bound), and ε_post_ (postupgrade error rate threshold). All thresholds are established during pilot deployments, documented in consortium standard operating procedures (SOPs), and calibrated to each clinical task’s criticality and site capabilities. Table 4 summarizes these operational playbooks, specifying for each failure mode its trigger condition, automated response procedures, and monitoring signals, and the associated on-chain audit events. These playbooks link directly to the verifiability and auditability layer discussed in the Auditability and Verifiability in BCFL section, ensuring that every remediation event is immutably logged and cryptographically attestable on-chain.

**Table 4.** Operational failure protocols for blockchain-based federated learning in health care (thresholds set via governance).

Failure mode	Trigger condition	Response playbook	Monitoring signals	Audit events
Client dropout	Quorum *< 𝜏*_quorum_^a^ after Θ_round_^b^; Byzantine clients *>* 𝜏_byz_^c^	Proceed with partial aggregation using Byzantine-robust methods [388]; checkpoint current state; suspend chronic offenders; re-enroll upon verification	Participation rate, round latency, missing sites, straggler ratio	Round degraded, site suspended, site reenrolled
Model drift	AUROC^d^ drop > 𝛿AUROC^e^; ECE^f^ increase > 𝛿ECE^g^; PSI^h^ > τ^major^_PSI_^i^	Pause promotion; perform shadow evaluation; rollback if unresolved; initiate root cause analysis	AUROC by cohort, ECE by cohort, PSI, drift alerts	Drift alert, model freeze, model promote, model rollback
Contract upgrade	Security vulnerability, policy change, or tx^j^ failure rate > 𝜏txfail^k^	Execute time locked multisig proxy upgrade; perform staged rollout; autorevert if postupgrade errors *>* 𝜀post^l^	tx failure rate, version mismatch, event gaps	Upgrade proposed, upgrade executed, upgrade reverted
Key compromise	Unexpected signer behavior, attestation failure, or CRL^m^ entry detected	Revoke key; rotate via threshold MPC^n^ [389]; reattest using HSM^o^/TEE^p^; reaggregate any tainted data	Signature verify fail rate, unexpected signer count, anomalous payouts	Key revoked, key rotated, site re-enrolled

a τ_quorum_: minimum client quorum.

b Θ_round_: round deadline.

c τ_byz_: maximum tolerated Byzantine clients for robust aggregation.

d AUROC: area under the receiver operating characteristic curve.

e δ_AUROC_: maximum allowed AUROC drop.

f ECE: expected calibration error.

g δ_ECE_: maximum allowed ECE increase.

h PSI: population stability index.

i τmajor_PSI_: PSI bound for major drift.

j tx: transaction.

k τ_txfail_: transaction failure rate bound.

l ε_post_: postupgrade error rate threshold.

m CRL: certificate revocation list.

n MPC: multiparty computation.

o HSM: hardware security module.

p TEE: trusted execution environment.

#### Case Study: Multi-Institutional Brain-Tumor Segmentation

To concretely illustrate these operational protocols, we consider BCFL deployment for glioblastoma segmentation across 3 academic medical centers (hospitals A, B, and C) utilizing the federated tumor segmentation benchmark [390]. Hospital A serves as the semicoupled aggregator in this consortium. The workflow encompasses the following: (1) *data types and preparation*, where each institution contributes multimodal DICOM MRI scans (T1, T1Gd, T2, and FLAIR sequences) accompanied by expert-annotated segmentation masks for enhancing, necrotic, and edema tumor subregions; (2) *consent and governance*, managed through smart contracts encoding tiered patient permissions that allow model training, permit secondary research (opt-in), and prohibit commercial use; (3) *training execution*, where each hospital trains a local 3D U-Net model on its data, computing gradient hashes *h*(∇*W_i_*) submitted to the blockchain via the ModelUpdate smart contract after each federated round; and (4) *operational resilience*, demonstrated when hospital B experiences a network outage mid-round—the system automatically proceeds using the quorum threshold τ_quorum_ = 0.67 (the quorum threshold τ_quorum_ = 0.67 ensures BFT consistent with standard BFT safety guarantees [n ≥ 3*f* + 1], allowing global aggregation to proceed only when at least two-thirds of clients contribute authenticated updates), and after 3 consecutive missed rounds, hospital B is suspended until manual infrastructure verification. This end-to-end scenario demonstrates how BCFL protocols maintain continuity in collaborative clinical research despite real-world operational challenges.

### Regulatory-Compliant Integration

#### Overview

The integration of blockchain with FL in health care environments requires strict adherence to comprehensive regulatory frameworks governing patient data protection, medical device validation, and cross-jurisdictional data exchange. This section analyzes architectural approaches for achieving regulatory compliance while preserving the security and privacy benefits of BCFL.

#### Regulatory Framework Overview

Health care data processing operates under multiple regulatory frameworks with stringent requirements for data handling, storage, and sharing. In the United States, HIPAA [391] establishes comprehensive privacy and security standards for protected health information (PHI), mandating administrative, physical, and technical safeguards [15]. Similarly, the GDPR [392] in the European Union requires explicit consent for data processing, adherence to data minimization principles, and provision of data portability and erasure rights. These regulatory requirements create significant challenges for conventional centralized ML approaches, often requiring detailed audit trails, comprehensive data lineage tracking, and the ability to selectively remove individual patient contributions from trained models [393].

The proliferation of AI and ML in medical applications has introduced additional regulatory considerations. The FDA framework for AI/ML-based medical devices emphasizes transparent model development, continuous performance monitoring, and the ability to track model behavior across diverse patient populations [394]. These requirements align well with BCFL capabilities, where immutable audit trails and decentralized validation mechanisms provide the transparency and accountability necessary for regulatory compliance in distributed settings [394].

Table 5 maps these key regulatory obligations to concrete FL and blockchain design mechanisms, highlighting how BCFL architectures can be engineered to support compliance across HIPAA, GDPR, and FDA requirements.

**Table 5.** Mapping of HIPAA^a^, GDPR^b^, and FDA^c^ requirements to FL^d^ and blockchain design mechanisms.

Guideline and key requirement		FL plus blockchain design mechanisms
HIPAA		
Privacy: PHI^e^ confidentiality; Security: electronic PHI, integrity/availability; Minimum necessary use		FL data locality, encrypted model updates, blockchain stores only hashed metadata (not PHI); Permissioned blockchain with BFT^f^ consensus, tamper-evident logs, redundant FL storage; Local feature selection, DP^g^ and gradient clipping, blockchain smart contracts enforce data policies
Audit controls		Immutable blockchain ledger for access/update logs, fine-grained access control lists, cryptographic signatures
Transmission security		TLS^h^ for FL channels, authenticated blockchain overlays, health care public key infrastructure integration
GDPR		
Lawfulness, fairness, transparency; Purpose limitation; Data accuracy; Right to erasure		Blockchain consent smart contracts, transparent logging, explainable FL models; Task-specific FL models, local data filtering, blockchain policies restrict model reuse; Cross-institutional FL validation, blockchain model lineage, rollback capability; Off-chain personal data, blockchain pseudonymous references, key revocation and model forgetting
Privacy by design		FL with DP/secure aggregation, minimal on-chain data, role-based blockchain permissions
Data protection impact assessment		Blockchain logs as impact assessment evidence, threat modeling, anomaly detection for poisoning/leakage
FDA		
Software as a medical device lifecycle control; Good machine learning practice and validation		Blockchain-anchored model versioning, immutable training/deployment records; Federated evaluation across sites, blockchain-backed data/model provenance
Postmarket monitoring		Continuous FL updates, blockchain logs for drift/adverse events, auto-rollback triggers
Change management		Blockchain registry for approved versions, on-chain governance for model rollout
Cybersecurity integrity		Signed FL/blockchain nodes, remote attestation, blockchain-based software bill of materials/model bill of materials tracking

a HIPAA: Health Insurance Portability and Accountability Act.

b GDPR: General Data Protection Regulation.

c FDA: Food and Drug Administration.

d FL: federated learning.

e PHI: protected health information.

f BFT: Byzantine fault tolerance.

g DP: differential privacy.

h TLS: transport layer security.

#### Compliance Challenges in FL

Traditional FL implementations face several compliance challenges in health care environments [395]. Data governance becomes complex when training spans multiple institutions with different privacy policies and jurisdictional requirements. Cross-border data sharing restrictions, exemplified by national data localization laws, can limit the scope of federated collaborations [396]. The distributed nature of FL can complicate compliance auditing, as conventional centralized monitoring approaches are inadequate for tracking model updates and validating data usage across multiple participating nodes. Model explainability requirements present another challenge, given increasing regulatory demands for transparent decision-making processes in clinical AI systems [397]. The aggregated nature of federated model updates can obscure individual data source contributions, hindering the provision of detailed explanations required for regulatory compliance and clinical validation.

#### Blockchain-Enhanced Compliance Architecture

Blockchain technology provides fundamental mechanisms that directly address regulatory compliance requirements in federated health care systems. The immutable distributed ledger creates comprehensive audit trails that record all model updates, participant contributions, and data access events, satisfying requirements for detailed recordkeeping and accountability. Smart contracts can automate compliance verification by embedding regulatory requirements directly into the blockchain protocol, ensuring that data sharing and model training activities automatically conform to predefined privacy and security standards.

The 3 integration architectures offer varying degrees of compliance capabilities. First, for the fully-coupled architecture, all FL operations are recorded on the blockchain ledger, providing maximum transparency and auditability. Each model update is cryptographically signed and timestamped, creating an immutable record of the entire training process. This architecture suits environments requiring the highest levels of regulatory oversight but may face scalability constraints with high-frequency model updates. Second, the semicoupled architecture is a hybrid approach that selectively records critical compliance events on the blockchain while maintaining routine operations off-chain. Compliance-relevant activities, such as participant authentication, consent management, and model validation, are tracked on-chain, while frequent model updates occur off-chain with periodic on-chain checkpoints. This balance provides robust compliance capabilities while addressing scalability concerns. Third, for the loosely-coupled architecture, the blockchain primarily serves as a coordination and reputation management layer, with detailed compliance tracking implemented through conventional audit mechanisms. This approach offers maximum flexibility for integration with existing health care IT infrastructure but requires careful design to ensure adequate regulatory compliance.

#### Auditability and Verifiability in BCFL

Beyond confidentiality and integrity guarantees, BCFL must also provide transparent mechanisms for independent verification and traceability of all learning activities. To ensure transparent operations, verifiability and auditability in BCFL are defined as complementary properties that enable independent validation of all training activities without compromising privacy. Verifiability denotes the ability of any authorized entity to cryptographically confirm the authenticity and integrity of model updates, aggregation results, and participating institutions through signed digital evidence recorded on-chain. Auditability extends this notion by enabling authorized auditors to reconstruct and verify the entire lifecycle of the learning process (including the provenance of training rounds, participant consent scope, and compliance checks) using immutable ledger entries and corresponding off-chain records.

In the proposed framework, each local model update (∆*W_i_*) is hashed (eg, SHA-256) and digitally signed by the participant before being submitted to the blockchain. The on-chain record contains the following: (1) the participant’s pseudonymous identifier, (2) the signed update hash, (3) the round number and timestamp, (4) a consent-proof token (linking to consent metadata stored off-chain), and (5) the result of automated policy-compliance checks executed via smart contracts. Full model parameters, gradients, raw medical data, and detailed computational logs remain off-chain within secure institutional storage to preserve privacy and reduce blockchain overhead. This hybrid design allows auditors to verify consistency between on-chain commitments and off-chain artifacts, providing cryptographically verifiable evidence of model provenance, policy adherence, and regulatory compliance while maintaining confidentiality of protected health information.

#### Case Study: Regulatory Verification

Building upon brain-tumor segmentation deployment, a health care regulator conducts comprehensive compliance verification through systematic analysis of on-chain evidence across the following four critical dimensions: (1) *consent verification*, achieved by querying the ConsentRegistry smart contract to cryptographically validate that all training data possess active consent tokens with appropriate scope limitations and no revocation events during the model training period; (2) *model provenance audit*, tracing the complete lineage of the global segmentation model through immutable, timestamped update records that capture each hospital’s contribution sequence and aggregation proofs, thereby creating an auditable chain of custody from raw gradients to the final model; (3) *policy compliance validation*, verifying through smart contract execution logs that every model update underwent automated HIPAA security rule validation and Institutional Review Board (IRB) requirement checks prior to integration into the global model; and (4) *data governance assurance*, confirming through ZKP attestation that all patient data processing remained within institutional boundaries, with only cryptographic commitments—never raw medical images or sensitive patient information— traversing the network. This comprehensive verification process, fully reconstructible from blockchain evidence, demonstrates how the semicoupled BCFL architecture (Figure 9) enables rigorous regulatory oversight. The on-chain audit trail provides concrete verification artifacts, as follows:

Block #18345: hospital*_A_* → *h*(∇*W_A_*) + timestamp + consent proof + HIPAA check; Block #18346: hospital*_C_* → *h*(∇*W_C_*) + timestamp + consent proof + HIPAA check; Block #18347: aggregator → *h*(*W*_global_) + aggregation proof + ZKP attestation

This immutable record preserves patient privacy through cryptographic guarantees while maintaining institutional autonomy through verifiable local computation.

#### Cross-Border Compliance Considerations

International health care collaborations must navigate different regulatory requirements across jurisdictions [398]. BCFL architectures can incorporate jurisdiction-specific compliance rules through modular smart contract designs. Participants from different regulatory environments can implement their local compliance requirements while engaging in collaborative model training. The blockchain ledger can maintain separate compliance tracks for different jurisdictions [399], ensuring each participant’s regulatory obligations are met while enabling global collaboration. Data localization requirements are addressed through FL’s inherent data locality principle, where sensitive patient data remain within its originating jurisdiction. The blockchain component facilitates cross-border collaboration by managing model updates and coordination without requiring direct data sharing, satisfying both privacy regulations and data sovereignty requirements.

#### Implementation Guidelines for Regulatory Compliance

Successful deployment of regulatory-compliant BCFL requires careful attention to several implementation considerations. Organizations must establish clear data governance frameworks that define roles, responsibilities, and accountability measures for all participating entities [400]. Consent management systems must be integrated into the blockchain architecture to ensure patient permissions are tracked and honored throughout the FL process [12]. The technical infrastructure must support both the scalability demands of FL and the audit trail requirements of regulatory compliance [401]. This includes implementing robust key management systems for cryptographic operations, establishing secure communication channels between participating nodes, and designing backup and recovery procedures that maintain compliance during system failures. Regular compliance auditing must be embedded in the system design, with automated monitoring capabilities to detect potential regulatory violations and alert administrators to compliance issues. The blockchain’s immutable audit trail provides the foundation for these monitoring systems, enabling continuous compliance verification and rapid response to potential violations.

#### Ethical and Patient-Centric Considerations

Beyond regulatory and technical safeguards, ethical considerations are essential for responsible BCFL deployment in health care. The decentralized nature of FL and blockchain complicates the acquisition and management of patient consent across autonomous institutions with diverse governance frameworks [402,403]. Traditional consent models, designed for single-institution data use, are inadequate when patient data contribute to shared models with different stakeholders and objectives [402]. To address this, consent management should leverage verifiable cryptographic mechanisms, such as consent tokens, ZKPs, and smart contract–based registries, to enable patients to grant, track, and revoke permissions without disclosing sensitive data [12,404]. These mechanisms uphold autonomy and transparency while aligning with evolving interpretations of HIPAA and GDPR.

Blockchain immutability also conflicts with the GDPR’s “right to be forgotten” (Article 17) [405,406]. To reconcile this, BCFL designs should store personally identifiable data off-chain while recording only hash-based consent attestations and model metadata [407,408]. This preserves auditability and enables consent withdrawal without breaching immutability.

Fairness is equally critical, as institutions with greater computational capacity or larger datasets may dominate model updates, biasing outcomes against underrepresented groups [409,410]. Mitigation requires fair aggregation [403], representative datasets [411], and fairness-aware or distributionally robust optimization ensuring consistent performance across demographics [412,413].

Ethical governance mechanisms, including IRBs [414,415] and data ethics councils [416,417], should oversee consent processes, fairness evaluations, and the protection of vulnerable populations [414,418]. Clear accountability across AI developers, health care providers, and blockchain participants is vital [419]. Finally, explainable AI approaches [420-422] must ensure that model decisions are interpretable by clinicians and understandable to patients, reinforcing trust and autonomy. Ethical BCFL thus requires a shift from a compliance-driven design to a patient-centric design, where transparency, fairness, and accountability become foundational principles for building trust and ensuring sustainable adoption of collaborative health care AI systems [402,423].

#### Ethics and Patient Voice

Ethical deployment of BCFL in health care requires patient-centric design, translating consent, autonomy, and transparency into actionable system features. Consent should evolve from binary opt-in to granular, preference-based models, allowing patients to specify which data types (eg, EHRs, genomic data, and imaging data) and purposes are permitted through layered interfaces [424,425]. A revocation mechanism records consent withdrawal as an on-chain event, preventing further participation. While revocation cannot retroactively remove previously aggregated updates, federated unlearning protocols mitigate historical influence by efficiently retraining global models without revoked participants [426,427]. Meaningful transparency extends beyond cryptographic verifiability to interpretable communication, and systems should notify contributors when models trained on their data are deployed and provide accessible indicators of model performance, bias, and fairness. When BCFL outcomes inform clinical decisions, explainable AI (XAI) techniques (eg, Shapley Additive Explanations [SHAP] and confidence decomposition) support instance-level explanations that bridge distributed model complexity with clinician and patient understanding [422,428,429].

Embedding granular consent, verifiable revocation, and interpretable transparency within the BCFL architecture ensures that distributed health care AI respects patient autonomy; aligns with regulatory obligations under HIPAA, GDPR, and the FDA’s AI/ML-based SaMD action plan [430]; and fosters sustained public trust in collaborative health systems [431].

#### Future Regulatory Developments

The regulatory landscape for AI in health care continues to evolve, with new guidelines and requirements emerging regularly. BCFL architectures must be designed with flexibility to accommodate changing regulatory requirements without requiring a complete system redesign. The modularity of smart contract–based compliance frameworks provides this adaptability, enabling organizations to update their compliance mechanisms as regulatory requirements evolve. Emerging regulatory trends, such as algorithmic accountability requirements and mandatory AI explainability, are well-suited to blockchain-based approaches that provide transparent audit trails and verifiable model development processes. As health care organizations increasingly adopt AI and ML technologies, the compliance advantages of BCFL will become increasingly important for regulatory acceptance and clinical adoption.

## Empirical Validation and Performance Evaluation

### Overview

This section grounds our BCFL integration in evidence from clinical deployments, consortia-scale studies, and controlled benchmarks. We synthesize outcomes along four axes: (1) accuracy versus centralized baselines; (2) scalability and latency/throughput under permissioned consensus; (3) robustness and privacy under realistic threat models; and (4) compliance, provenance, and operational constraints. Where possible, we report representative ranges from cited studies rather than single-point claims.

### Large-Scale Clinical and Multi-Institutional Evidence

Table 6 summarizes emblematic deployments. Across drug discovery (MELLODDY [253]), neuro-oncology (federated tumor segmentation [390]), pandemic diagnostics [432], and IoMT monitoring [251,433], BCFL consistently delivers accuracy within approximately 2%-3% of centralized training while providing auditability and coordination via permissioned chains or distributed ledgers.

**Table 6.** Representative blockchain-based federated learning deployments in health care.

Setting/cohort	Scale	Task	Blockchain role	Accuracy	Latency/overhead	Source
MELLODDY (10 pharma companies)	>2.6 billion data points, 21 million compounds	Drug prediction	Distributed ledger for auditability, consensus, traceability	Median ∼2% RIPtoP^a^ gain	Comparable to centralized	[253]
FeTS^b^ (71 sites)	71 international sites	Brain tumor segmentation	Coordination and registry	Within 2%‐3% of centralized	Real-world federation	[434,435]
COVID-19 consortium	Multinational	CT^c^ scan diagnosis	Aggregation logging, verification	94.2% versus 95.1% centralized	∼3.2 s/round	[433]
IoMT^d^ monitoring	50 patients	Anomaly detection	Consent, notarization	92.7%	<5 s for alerts	[436,437]
EHR^e^ management	Multi-institutional	Clinical analytics	Model validation, audit trails	95.2%	<150 ms blockchain latency (10,000 transactions)	[344]

a RIPtoP: relative improvement of proximity to perfection.

b FeTS is primarily a federated learning platform; blockchain integration is demonstrated in other health care applications listed here.

c CT: computed tomography.

d IoMT: Internet of Medical Things.

e EHR: electronic health record.

### Performance and Scalability Benchmarks

Controlled experiments report stable training under 50‐200 participants with permissioned consensus, and recent empirical studies demonstrate that blockchain-enabled FL can reduce communication overhead by 40%‐60% through communication-aware aggregation schemes [339,344]. Specifically, there was a 43% communication overhead reduction and a 37% lower computational cost while maintaining 95.2% model accuracy [344]. Sharded or committee-based designs sustain 80-120 TPS at the consortium scale while keeping the consensus overhead under 5% for ≤50 nodes, and at 100 nodes, PBFT-style protocols exhibit 12%-15% overhead [438,439].

### Security, Privacy, and Robustness

Against gradient inversion and membership inference, combining DP (eg, *ϵ*<0.1) with encrypted or masked updates sustains utility within 3%‐5% of nonprivate baselines at an approximately 2.8 times compute overhead, while Byzantine-robust aggregation maintains <5% degradation under up to 30% compromised clients [19,352]. Recent work has demonstrated that blockchain-enabled frameworks maintain robustness against multiple adversarial attack vectors with accuracy levels above 93% [344]. For verifiable compliance, ZKPs amortize to proof generation of 8‐15 s and verification of 2‐4 s per minute-scale update [440].

### Compliance, Provenance, and Operational Considerations

Empirical audits indicate technical adherence to approximately 93% of HIPAA/GDPR provisions, with 70%‐80% manual audit effort reduction when provenance is automated via smart contracts [395]. For FDA transparency obligations on AI/ML devices, immutable lineage reduces evidence cycles from an initial 6‐9 months to 2‐3 months in reported pipelines [397]. Operational deployment of blockchain-enabled FL systems faces several infrastructure challenges, including maintaining high system availability, managing blockchain storage growth as institutions scale, balancing computational overhead from cryptographic operations, and ensuring adequate network bandwidth for model parameter exchange [9,441].

### Architectural Tradeoffs

In permissioned health care networks, PoA achieves 200-300 TPS and approximately 95% lower energy than PoW, whereas PBFT offers stronger Byzantine resilience at 40%‐50% lower throughput [442]. Comparative syntheses show that BCFL improves privacy metrics by 40%‐60% over vanilla FL while staying within approximately 3% of centralized accuracy [344,384]. Recent systematic reviews covering literature from 2023‐2024 confirm these patterns across diverse health care applications [12,400]. Fully-coupled blockchain-FL designs minimize latency (reduction of 30%‐40%) but demand 2‐3 times the resources, while semicoupled architectures offer a balanced middle ground [443].

### Threats to Validity and Replicability

#### Information

A crucial aspect of interpreting empirical studies on BCFL involves recognizing their inherent threats to validity. Specifically, researchers must account for the impact of various factors. First, non-independent and identically distributed data and silo imbalance are important aspects, where skewed label and cohort distributions across participating institutions necessitate the reporting of per-site metrics and federated confusion matrices. Second, WAN effects must be meticulously reported for wide-area deployments, including round times, network latency, bandwidth, and specific block/endorsement parameters, to avoid misleading claims based on LAN-only results. Third, attack realism requires results to be reproducible with adaptive rather than static poisoning, along with full disclosure of clipping rules, aggregation mechanisms, and defense strategies. Fourth, from a privacy perspective, privacy budgets must be transparent, demanding the publication of (ϵ,δ) values, sensitivity assumptions, and composition accounting across all training rounds. Fifth, achieving determinism is essential for reproducibility, requiring the release of seeds, exact model/optimizer configurations, client sampling methods, and chaincode/smart contract versions. Finally, accurate compute/power accounting must distinguish between the computational overhead dedicated to model training versus the overhead attributed to blockchain operations, detailing the validator count and hardware thermal design power (TDP). Recent comprehensive reviews have systematically identified these validity concerns across the literature from 2018‐2024 [384,400].

#### Takeaway

Across clinically meaningful tasks, BCFL achieves near-centralized utility with measurable but manageable overheads when engineered with communication-efficient aggregation and permissioned consensus. Recent empirical evidence from 2024‐2025 confirms consistent patterns: accuracy within 2%‐3% of centralized baselines, communication overhead reductions of 40%‐50%, and successful deployment across 50‐200 participating institutions. The primary value additions in health care are auditability, provenance for regulatory processes, and robustness under adversarial and privacy constraints, while the primary bottlenecks are large-scale coordination (≥500 nodes) and legacy-system interoperability.

## Related Work

### Overview

In this section, we comprehensively explore existing research endeavors focusing on integrating BCFL frameworks within health care contexts. Initially, we delve into the corpus of literature dedicated to elucidating the practical applications, methodologies, and outcomes of utilizing blockchain technology in conjunction with FL for health care use cases. In addition, we aim to provide a taxonomy and categorize the existing work based on the insights garnered throughout this paper. To achieve this, we classify the research according to integration architecture (ie, fully coupled, semicoupled, and loosely coupled), blockchain platform, FL type, and data type. Furthermore, we highlight their primary contributions as well as their limitations. Later, we examine existing surveys and reviews focusing on blockchain-assisted FL in health care. This will provide insights into the overall research landscape and help identify any gaps or areas for further exploration.

### Literature Review

Samantray and Reddy [444] proposed a hybrid blockchain architecture with quantum key encryption for Healthcare 5.0. Bhasker et al [445] presented a Healthcare 5.0 smart health care system for addressing confidentiality, trust, and compliance standards for safe health monitoring. Bhardwaj et al [428] introduced a privacy-preserving Federated Blockchain Explainable Artificial Intelligence Optimization (PPFBXAIO) framework for the integration of blockchain and XAI, and optimization techniques to ensure privacy, traceability, and robustness in FL-based systems. Ali et al [446] presented a ZKP and HE approach for EHR security. Das et al [447] presented a meta-learning approach for improved model generalization.

Chen et al [19] proposed a trustworthy and fair blockchain-based FL framework to address challenges like malicious attacks (model/data poisoning) and free-rider behavior. In another study [448], a quality-aware BCFL with a secure key-sharing mechanism to protect local model parameters and ensure data security was proposed. Mazid et al [436] proposed a blockchain-driven FL framework for secure health care services using IoMT devices. Liang et al [449] proposed a software architecture integrating FL and blockchain to mitigate bias and fairness issues in health care predictive modeling while safeguarding patient privacy. Om Kumar et al [450] introduced a mechanism to reward organizations participating in the FL process, ensuring privacy-preserving model transfer using loosely-coupled integration with the Ethereum blockchain. Moulahi et al [437] integrated FL and blockchain to develop a trusted system for predicting diabetes risk while ensuring data privacy and model integrity.

Ali et al [451] focused on integrating blockchain with FL for secure and decentralized analysis of electronic medical records in precision medicine. Chang et al [442] proposed an integration of adaptive DP and gradient verification-based consensus protocols in a fully-coupled architecture for health care analytics. Lian et al [452] presented a blockchain-based personalized FL system for ensuring security and privacy in IoMT.

Farooq et al [453] developed an automated system for analyzing patients’ live data within a fully-coupled architecture, while Zhang et al [454] proposed a blockchain-enabled FL framework for health care data privacy protection. Aich et al [336] introduced a blockchain-assisted FL framework for personal data preservation, and Passerat-Palmbach et al [335] proposed a novel architecture for FL integrated with blockchain within health care systems. A taxonomy of existing research studies, along with additional relevant work, has been compiled in Table 7.

**Table 7.** Related work on blockchain-assisted FL^a^ in health care.

Reference	Main contribution	Architecture	Blockchain platform	FL type	Data type	Limitations
Chen et al [19]	Introduces a trustworthy and fair blockchain-based FL framework (FedCFB) to address challenges like malicious attacks (model/data poisoning) and free-rider behavior	Semicoupled	Custom-designed	HFL^b^	Unknown (MNIST)	No discussion on throughput/latency tradeoffs for large-scale FL deployments.
Samantray and Reddy [444]	Hybrid blockchain architecture with quantum key encryption for futuristic health care in smart cities	Loosely coupled	Ethereum	HFL	Medical images and documents	Performance and scalability issues in large-scale implementations.
Bhasker et al [445]	A health care 5.0 smart health care system for addressing confidentiality, trust, and compliance standards for safe health monitoring	Loosely coupled	Unknown	HFL	Wearable devices and sensors	Lack of real-world deployment scenarios and practical validation in actual health care settings.
Bhardwaj and Sumangali [428]	Proposes a privacy-preserving federated blockchain explainable artificial intelligence optimization (PPFBX- AIO) framework, which integrates blockchain, explainable artificial intelligence, and optimization techniques to ensure privacy, traceability, and robustness in FL-based systems	Fully coupled	Unknown	HFL	EHRs^c^	Real-world deployment gaps (ie, does not address practical deployment challenges, such as regulatory compliance with health care standards (HIPAA^d^ or GDPR^e^) and integration with existing hospital information systems.
Ali et al [446]	Leverages zero-knowledge proofs and homomorphic encryption for EHR security	Semicoupled	Unknown	HFL	EHRs	Limited empirical validation or real-world testing of the proposed framework.
Das et al [447]	Meta-learning approach for improved model generalization	Semicoupled	Hyperledger Besu	HFL	IoMT^f^ data	No real-world implementation (the entire study is simulation-based with no actual deployment and no real medical IoT^g^ devices involved), incomplete blockchain implementation, and FL implementation is unclear.
Munusamy and Jothi [344]	Proposes an enhanced privacy-preserving blockchain-enabled federated learning (EPP-BCFL) framework that integrates blockchain with hybrid privacy mechanisms and intelligent aggregation strategies for secure EHR	Semicoupled	Hyperledger Fabric	HFL	Medical imaging data (CIFAR-10)	Evaluation limited to CIFAR-10 rather than clinical datasets.
Lo et al [455]	Proposes a BCFL^h^ architecture to enable accountability in FL systems.	Loosely coupled	Ethereum	HFL	Medical imaging data (COVID-19 CT^i^)	Lack of real-world deployment analysis.
Kumar et al [448]	Proposes a quality-aware BCFL with a secure key-sharing mechanism to protect local model parameters and ensure data security	Loosely coupled	Unknown	HFL	Unknown	Limited scale and real-world testing (eg, not tested in actual hospital environments, no real health care institutions involved, etc).
Mazid et al [436]	Proposes a blockchain-driven FL framework for secure health care services using IoMT devices	Loosely coupled	Unknown	HFL	Medical imaging (colon pathology, breast tumor)	Validated only through simulations, using benchmark datasets (2D colon pathology, breast tumor, and CIFAR-10). There is no real-world hospital or IoMT deployment, which limits conclusions.
Liang et al [449]	Software architecture integrating FL and blockchain to tackle bias and fairness issues in health care predictive modeling while safeguarding patient privacy	Semicoupled	Rahasak	Unknown	Unknown	Limited real-world validation. This study was conducted in a simulated environment with only 5 simulated medical centers rather than actual health care institutions. This significantly limits the generalizability of findings to real-world health care settings.
Om Kumar et al [450]	Introduces a mechanism to reward organizations participating in the FL process, ensuring privacy-preserving model transfer between users and organizations using blockchain	Loosely coupled	Ethereum	HFL	Medical imaging data (COVID-19 CT)	Not explicitly stated.
Moulahi et al [437]	Integrates FL and blockchain technology to develop a trusted system for predicting diabetes risk while ensuring data privacy and model integrity	Semicoupled	Ethereum	HFL	Sensor data (IoT)	Not explicitly stated.
Ali et al [451]	Integrates blockchain with FL to enable secure and decentralized analysis of EMRs in precision medicine	Loosely coupled	Ethereum, Hyperledger Fabric (simulation)	HFL	EMR	The paper appears to be primarily theoretical and simulation-based without actual deployment in real health care settings (eg, there is no evidence of testing with actual health care institutions or real EMR) and limited experimental validation (eg, no specific dataset characteristics are provided).
Chang et al [442]	Integrates adaptive differential privacy and gradient verification-based consensus protocols	Fully coupled	Ethereum	HFL/VFL^j^	IoMT sensor data	Scalability challenges, increased complexity, potential computational overhead, and the need for further validation across diverse medical conditions and datasets.
Lian et al [452]	Blockchain-based personalized FL system to address security and privacy concerns in IoMT	Fully coupled	Unknown (consortium PoS^k^ blockchain)	VFL	IoMT sensor data (Fashion-MNIST)	Limited experimental validation (eg, only simulated experiments were used rather than real-world deployment, tested on Fashion-MNIST and CIFAR-10 [image classification datasets]; no actual medical data, etc).
Farooq et al [453]	Develops an automated system for analyzing patients’ live data	Fully coupled	Ethereum	HFL	IoMT sensor data	Limited empirical validation or real-world testing of the proposed framework.
Zhang et al [454]	Proposes a BCFL framework for health care data privacy protection	Fully coupled	Unknown (conceptual)	HFL	EHR (MNIST)	Theoretical model only, not supported via experimental implementations.
Aich et al [336]	Proposes a blockchain-assisted FL framework for personal data preserving	Loosely coupled	Unknown (conceptual)	—^l^	No data	Reliance on conceptual assumptions without real-world application.
Passerat-Palmbach et al [335]	Proposes a novel architecture for FL integrated with blockchain within a health care system	Loosely coupled	Ethereum	—	No data	Not explicitly stated.
Rahman et al [438]	Proposes a BCFL framework for COVID-19 applications to classify IoHT^m^ data	Loosely coupled	Ethereum	FL type	Sensor data (IoMT)	Not explicitly stated.
Singh et al [456]	Integrates blockchain technology with FL to enhance privacy preservation and scalability in health care data management	Fully coupled	Unknown	Unknown	Sensor data (IoT)	Theoretical model only, not supported via experimental implementations.
Nguyen et al [457]	Proposes a new blockchain-enabled FL-based generative adversarial network framework for secure COVID-19 data analytics	Semicoupled	Unknown (conceptual)	HFL	Medical imaging data (COVID-19 CT)	The blockchain model is conceptual, and a real-world implementation was not reported.
Liu et al [458]	Proposes a framework of blockchain-empowered FL in health care–based cyber-physical systems	Fully coupled	Ethereum	HFL	EHR (MNIST HAM10000)	Not explicitly stated.
Otoum et al [459]	Proposes a novel solution for revolutionizing health care systems by considering concepts like distributivity, self-learnability, and autonomy.	—	Unknown (conceptual)	Unknown	No data	Only introduces a theoretical framework without an empirical validation.
Kumar et al [433]	Blockchain-empowered method to detect patterns of COVID-19 from lung CT scans	Fully coupled	Unknown	HFL	Medical imaging data (COVID-19 CT)	The paper does not extensively discuss the generalization capabilities of the proposed model to handle variations in COVID-19 manifestations across different patients.
Durga and Poovammal [460]	A concise review of BCFL in health care, concepts, and taxonomy	—	Unknown	Unknown	Unknown	The article is very brief and does not cover all concepts.
Lakhan et al [461]	Proposes a FL-based blockchain-enabled task scheduling (FLBETS) framework with different dynamic heuristics for health care applications	Loosely coupled	Unknown	Unknown	Sensor data (IoMT)	Dynamic and run-time unknown attacks against IoMT were not considered in this work.
Samuel et al [462]	Introduces FedMedChain as a BCFL framework for medical data privacy preservation	Loosely coupled	Unknown	Unknown	Sensor data (Unknown)	Not explicitly stated.
Yang and Xing [463]	Introduces a privacy protection framework for medical data using blockchain and FL for secure and auditable data sharing among medical institutions using a secure aggregation scheme based on homomorphic encryption	Loosely coupled	Ethereum	HFL	EHR (MNIST different datasets)	Not explicitly stated.

a FL: federated learning.

b HFL: horizontal federated learning.

c EHRs: electronic health records.

d HIPAA: Health Insurance Portability and Accountability Act.

e GDPR: General Data Protection Regulation.

f IoMT: Internet of Medical Things.

g IoT: Internet of Things.

h BCFL: blockchain-based federated learning.

i CT: computed tomography.

j VFL: vertical federated learning.

k PoS: proof of stake.

l Not applicable/not available.

m IoHT: Internet of Healthcare Things.

### Existing Surveys

To the best of our knowledge, comprehensive surveys or reviews focusing on the integration of blockchain and FL for health care use cases are scarce. While individual studies have explored the potential of each technology independently within health care settings, there is a notable lack of resources delving into the synergistic benefits and challenges of combining blockchain’s immutable ledger capabilities with FL’s decentralized model training approach.

Ngoupayou Limbepe et al [12] presented a taxonomy of FL-based privacy mechanisms categorized into privacy-enhancing technologies and hybrid techniques. This survey integrates privacy-enhancing technologies with blockchain in FL frameworks for smart health care systems. Noteworthy initial explorations, such as those conducted by Myrzashova et al [13] and Nguyen et al [464], offer valuable insights but often lack a broader perspective. Myrzashova et al [13], for instance, analyzed the advantages and disadvantages of BCFL integration in health care, but they overlooked the importance of a data type taxonomy. Understanding the diverse medical data types (eg, genomics and imaging) used in health care ML is crucial, as different data may have varying security and privacy requirements. Similarly, Nguyen et al [464] introduced a new conceptual architecture that integrates blockchain and AI for combating the COVID-19 pandemic. While offering valuable insights into addressing specific challenges posed by the pandemic, its scope is limited to COVID-19 and related data, lacking a broader analysis of the integration’s potential for various health care use cases beyond this specific context. Hemdan et al [465] examined the convergence of digital twin technology, blockchain, and FL in the medical field, with a specific focus on their technical architecture and real-world applications. Orabi et al [400] presented a literature review of BCFL applications in health care for protecting sensitive data. This paper investigated how integrating FL with blockchain can enhance its security, performance, and reliability.

In contrast to these existing reports, this tutorial offers a more comprehensive perspective. We present a taxonomy of medical data used for ML, providing a foundational understanding of the diverse data types relevant to this integration. Furthermore, we unveil an innovative architecture meticulously designed for the seamless integration of blockchain and FL within health care systems, addressing the need for secure and privacy-preserving health care analytics.

## Future Research Directions

### Overview

The integration of FL and blockchain in health care introduces a transformative paradigm for secure, privacy-preserving, and collaborative medical AI systems. Despite notable theoretical advances, several research frontiers remain underexplored. These can be broadly categorized into 4 interrelated areas: cryptographic foundations and quantum-resilient privacy, scalable and interoperable infrastructure, health care–specific consensus and incentivization, and full-stack integration with regulatory automation.

### Cryptographic Foundations and Quantum-Resilient Privacy

A core requirement for the secure deployment of BCFL systems in health care is the development of advanced cryptographic mechanisms that ensure long-term privacy, data integrity, and resilience against quantum adversaries. Postquantum cryptographic primitives (such as lattice-based schemes like CRYSTALS-Kyber and Dilithium, and hash-based schemes like SPHINCS+) must be integrated into the BCFL stack to secure signatures and encryption layers [466]. In parallel, ZKPs and zero-knowledge virtual machines offer promising approaches to verifiably audit FL operations and regulatory compliance without exposing raw medical data. Furthermore, HE schemes, particularly multikey variants of the CKKS (Cheon-KimKim-Song) cryptosystem [467], enable secure aggregation of encrypted model parameters from multiple health care institutions without revealing individual contributions. The substantial computational overhead of such operations can be alleviated through specialized hardware accelerators, notably smart network interface cards, which offload cryptographic computations to dedicated processing units, thereby improving efficiency and scalability [468]. Complementarily, DP mechanisms require refinement to handle medical-specific feature types, with research needed into adaptive noise strategies and budget allocation tailored to data sensitivity. Together, these innovations will enable secure, auditable, and privacy-preserving collaborative learning in health care environments.

### Scalable and Interoperable BCFL Infrastructure

As clinical systems generate increasingly voluminous and heterogeneous data, BCFL infrastructure must address throughput, latency, and interoperability challenges to support real-time learning and inference [401,439]. Health care–specific sharding architectures (such as parallel blockchains for EHRs, medical imaging, and genomics) can significantly improve throughput by distributing model updates across application domains. Complementary to this, layer-2 scaling solutions, including rollups [469] and state channels, offer reductions in transaction costs and ledger bloat, making frequent model updates feasible without congesting the blockchain [470]. Additionally, cross-chain interoperability mechanisms, such as those based on the Cosmos IBC protocol or Polkadot’s relay chain model, are essential for enabling collaboration among health care institutions operating on heterogeneous blockchain platforms. Moreover, edge-centric FL will become increasingly important as IoMT and wearable devices proliferate. Furthermore, split learning and energy-efficient blockchain clients will be crucial to bring BCFL capabilities to constrained edge nodes while preserving security guarantees. These advancements will enable a scalable and interoperable ecosystem capable of supporting global health care collaborations.

### Health Care–Specific Consensus and Incentive Mechanisms

Generic blockchain consensus protocols are ill-suited for the privacy, latency, and regulatory demands of health care. There is a growing need to design health care–oriented consensus protocols, such as medical and practical BFT [471], which can minimize communication overhead and incorporate privacy-aware authorization and medical rule validation into the consensus process. Similarly, reputation-based BFT [472] can ensure the credibility of participating institutions and practitioners, dynamically adjusting voting power based on behavior and contribution history. To sustain active participation in FL processes, especially among resource-constrained or data-rich stakeholders, incentive mechanisms must be tailored to health care environments. The proof-of-federated-work paradigm introduces an innovative strategy to quantify participant contributions using metrics like local accuracy, data diversity, and compliance adherence [473]. These are integrated into token-based or hybrid compensation schemes, further reinforced by game-theoretic models that optimize incentives based on Nash equilibrium to deter free-riding and encourage high-quality model updates. Such mechanisms are foundational to the long-term viability of decentralized health care learning networks.

### System Integration, Compliance Automation, and Real-World Translation

For BCFL frameworks to be deployed at scale, they must integrate seamlessly with health care systems, satisfy regulatory requirements, and maintain usability in real-time clinical contexts. Smart contracts should evolve to support automated, upgradeable compliance verification that is capable of adapting to dynamic legal frameworks such as HIPAA, GDPR, and FDA AI guidelines [474]. By incorporating ZKP-based attestations, institutions can demonstrate compliance without revealing sensitive patient-level operations [440]. Simultaneously, embedding explainable AI techniques, such as SHAP values and attention heatmaps, into the FL pipeline is essential to ensure clinician trust and support regulatory transparency [475]. Algorithmically, innovations in personalized FL, multitask learning (eg, MOCHA [476]), and community-based learning [477] can help address patient heterogeneity and improve predictive power in distributed environments. Real-time FL capabilities, triggered by event-driven smart contracts (eg, emergency alerts), are crucial for responsive applications like ICU triage and chronic condition monitoring [432]. To ensure long-term sustainability, quantum-resistant blockchain architectures should be explored using crypto-agile protocols and hybrid classical/postquantum stacks. These systems must also support interoperability, modular compliance, and secure cross-border data flows, forming the backbone of globally federated, privacy-aware health care AI infrastructure.

## Conclusion

This tutorial introduced a comprehensive, clinically oriented, and compliance-aware framework integrating FL and blockchain for secure and privacy-preserving health care analytics. We demonstrated how FL enables decentralized model training across health care institutions while maintaining data locality and how blockchain enhances trust, integrity, and auditability through immutable ledgers and decentralized consensus mechanisms. Our key contributions include the following: (1) a systematic taxonomy of diverse medical data types and their FL requirements; (2) three novel integration architectures (fully coupled, semicoupled, and loosely coupled) with rigorous analysis of security, scalability, and regulatory compliance tradeoffs; (3) comprehensive security analysis identifying health care–specific vulnerabilities and mitigation strategies using advanced cryptographic techniques, including ZKPs, HE, and DP; and (4) a practical regulatory compliance framework addressing HIPAA, GDPR, and FDA guidelines for AI/ML-based medical devices. We validated BCFL effectiveness across critical health care applications, including disease prediction, medical imaging analysis, patient monitoring, and drug discovery. Looking ahead, crucial research frontiers involve quantum-resilient cryptography, scalable interoperable infrastructure, health care–specific consensus mechanisms, and automated compliance frameworks. This tutorial provides a foundational reference for developing trustworthy and patient-centric AI systems in health care. By integrating blockchain and FL, these systems can transform health care delivery while safeguarding privacy, ensuring regulatory compliance, and ultimately driving improved patient outcomes and accelerating medical discoveries in an increasingly connected health care ecosystem.

## Abbreviations

AI: artificial intelligence
BCFL: blockchain-based federated learning
BFT: Byzantine fault tolerance
CT: computed tomography
DAG: direct acyclic graph tangle
DApps: decentralized applications
DICOM: Digital Imaging and Communications in Medicine
DP: differential privacy
DPoS: delegated proof of stake
ECG: electrocardiography
EEG: electroencephalography
EHR: electronic health record
FDA: Food and Drug Administration
FL: federated learning
FTL: federated transfer learning
GDPR: General Data Protection Regulation
HE: homomorphic encryption
HFL: horizontal federated learning
HIPAA: Health Insurance Portability and Accountability Act
IBC: Inter-Blockchain Communication
IoMT: Internet of Medical Things
IoT: Internet of Things
MAR: medication administration record
ML: machine learning
MRI: magnetic resonance imaging
NLP: natural language processing
P2P: peer-to-peer
pBFT: practical Byzantine fault tolerance
PGD: patient-generated data
PoA: proof of authority
PoS: proof of stake
PoW: proof of work
PRO: patient-reported outcomes
RL: reinforcement learning
SHAP: Shapley Additive Explanations
SMPC: secure multiparty computation
VFL: vertical federated learning
ZKP: zero-knowledge proof

## References

[1] Ometov A, Shubina V, Klus L (2021). A survey on wearable technology: history, state-of-the-art and current challenges. Computer Networks.

[2] Kang HS, Exworthy M (2022). Wearing the future-wearables to empower users to take greater responsibility for their health and care: scoping review. JMIR Mhealth Uhealth.

[3] Wu M (2019). Wearable technology applications in healthcare: a literature review. Online J Nurs Inform.

[4] Zhang C, Xie Y, Bai H, Yu B, Li W, Gao Y (2021). A survey on federated learning. Knowl Based Syst.

[5] Babar M, Qureshi B, Koubaa A (2024). Review on federated learning for digital transformation in healthcare through big data analytics. Future Generation Computer Systems.

[6] Fang M, Cao X, Jia J, Gong N Local model poisoning attacks to byzantine-robust federated learning. https://www.usenix.org/system/files/sec20-fang.pdf.

[7] Kumar KN, Mohan CK, Cenkeramaddi LR, Awasthi N (2025). Minimal data poisoning attack in federated learning for medical image classification: an attacker perspective. Artif Intell Med.

[8] Bouacida N, Mohapatra P (2021). Vulnerabilities in federated learning. IEEE Access.

[9] Cai Z, Chen J, Fan Y, Zheng Z, Li K (2025). Blockchain-empowered federated learning: benefits, challenges, and solutions. IEEE Trans Big Data.

[10] Abbas SR, Abbas Z, Zahir A, Lee SW (2024). Federated learning in smart healthcare: a comprehensive review on privacy, security, and predictive analytics with IoT integration. Healthcare (Basel).

[11] Cheng H, Qu Y, Liu W, Gao L, Zhu T (2025). Decentralized federated learning for private smart healthcare: a survey. Mathematics.

[12] Ngoupayou Limbepe Z, Gai K, Yu J (2025). Blockchain-based privacy-enhancing federated learning in smart healthcare: a survey. Blockchains.

[13] Myrzashova R, Alsamhi SH, Shvetsov AV, Hawbani A, Wei X (2023). Blockchain meets federated learning in healthcare: a systematic review with challenges and opportunities. IEEE Internet Things J.

[14] Datta P, Namin AS, Chatterjee M A survey of privacy concerns in wearable devices.

[15] Qayyum A, Qadir J, Bilal M, Al-Fuqaha A (2021). Secure and robust machine learning for healthcare: a survey. IEEE Rev Biomed Eng.

[16] Cilliers L (2020). Wearable devices in healthcare: privacy and information security issues. Health Inf Manag.

[17] Lyu L, Yu H, Ma X (2024). Privacy and robustness in federated learning: attacks and defenses. IEEE Trans Neural Netw Learning Syst.

[18] Ye H, Liang L, Li GY (2022). Decentralized federated learning with unreliable communications. IEEE J Sel Top Signal Process.

[19] Chen L, Zhao D, Tao L (2024). A credible and fair federated learning framework based on blockchain. IEEE Trans Artif Intell.

[20] Gupta M, Kumar M, Dhir R (2024). Unleashing the prospective of blockchain-federated learning fusion for IoT security: a comprehensive review. Computer Science Review.

[21] Qu Y, Uddin MP, Gan C, Xiang Y, Gao L, Yearwood J (2023). Blockchain-enabled federated learning: a survey. ACM Comput Surv.

[22] Zhu J, Cao J, Saxena D, Jiang S, Ferradi H (2023). Blockchain-empowered federated learning: challenges, solutions, and future directions. ACM Comput Surv.

[23] Qammar A, Karim A, Ning H, Ding J (2023). Securing federated learning with blockchain: a systematic literature review. Artif Intell Rev.

[24] Ramakrishnaiah Y, Macesic N, Webb GI, Peleg AY, Tyagi S (2025). EHR-ML: a data-driven framework for designing machine learning applications with electronic health records. Int J Med Inform.

[25] Shen Y, Yu J, Zhou J, Hu G (2025). Twenty-five years of evolution and hurdles in electronic health records and interoperability in medical research: comprehensive review. J Med Internet Res.

[26] Nowrozy R, Ahmed K, Kayes ASM, Wang H, McIntosh TR (2024). Privacy preservation of electronic health records in the modern era: a systematic survey. ACM Comput Surv.

[27] Arbet J, Brokamp C, Meinzen-Derr J, Trinkley KE, Spratt HM (2021). Lessons and tips for designing a machine learning study using EHR data. J Clin Trans Sci.

[28] Wu H, Yamal JM, Yaseen A, Maroufy V (2020). Statistics and Machine Learning Methods for EHR Data: From Data Extraction to Data Analytics.

[29] Johnston SS, Morton JM, Kalsekar I, Ammann EM, Hsiao CW, Reps J (2019). Using machine learning applied to real-world healthcare data for predictive analytics: an applied example in bariatric surgery. Value Health.

[30] Muniasamy A, Tabassam S, Hussain MA, Sultana H, Muniasamy V, Bhatnagar R, Hassanien A, Azar A, Gaber T, Bhatnagar R, Tolba M (2020). The International Conference on Advanced Machine Learning Technologies and Applications (AMLTA2019) AMLTA 2019 Advances in Intelligent Systems and Computing.

[31] Singh P, Singh N, Singh KK, Singh A, Singh KK, Elhoseny M, Singh A, Elngar AA (2021). Machine Learning and the Internet of Medical Things in Healthcare.

[32] Ahsan MM, Luna SA, Siddique Z (2022). Machine-learning-based disease diagnosis: a comprehensive review. Healthcare (Basel).

[33] Komal Kumar N, Vigneswari D, Hura GS, Singh AK, Siong Hoe L (2021). Advances in Communication and Computational Technology ICACCT 2019 Lecture Notes in Electrical Engineering.

[34] Abhisheka B, Biswas SK, Purkayastha B, Das D, Escargueil A (2023). Recent trend in medical imaging modalities and their applications in disease diagnosis: a review. Multimed Tools Appl.

[35] Lalitha S, Sanjana T, Bhavana H, Bhan I, Harshith G, Shinde SV, Mahalle PN, Bendre V, Castillo O (2022). Disruptive Developments in Biomedical Applications.

[36] Santhi K (2022). A survey on medical imaging techniques and applications. J Innov Image Process.

[37] Mustapha MT, Uzun B, Ozsahin DU, Ozsahin I, Ozsahin I, Ozsahin DU, Uzun B (2021). Applications of Multi-Criteria Decision-Making Theories in Healthcare and Biomedical Engineering.

[38] Withers PJ, Bouman C, Carmignato S (2021). X-ray computed tomography. Nat Rev Methods Primers.

[39] De Pietro S, Di Martino G, Caroprese M (2024). The role of MRI in radiotherapy planning: a narrative review “from head to toe”. Insights Imaging.

[40] Avola D, Cinque L, Fagioli A, Foresti G, Mecca A (2022). Ultrasound medical imaging techniques. ACM Comput Surv.

[41] Cullen A, O’Connell M, Redmond C, Lee M (2020). Tutorials in Diagnostic Radiology for Medical Students.

[42] Wernick MN, Aarsvold JN (2004). Emission Tomography: The Fundamentals of PET and SPECT.

[43] Jones AK, Balter S, Rauch P, Wagner LK (2014). Medical imaging using ionizing radiation: optimization of dose and image quality in fluoroscopy. Med Phys.

[44] Ulrich H, Kock-Schoppenhauer AK, Deppenwiese N (2022). Understanding the nature of metadata: systematic review. J Med Internet Res.

[45] Bhuiyan MN, Rahman MM, Billah MM, Saha D (2021). Internet of things (IoT): a review of its enabling technologies in healthcare applications, standards protocols, security, and market opportunities. IEEE Internet Things J.

[46] Larobina M (2023). Thirty years of the DICOM standard. Tomography.

[47] Wang J, Wang S, Zhang Y (2025). Deep learning on medical image analysis. CAAI Trans on Intel Tech.

[48] Rana M, Bhushan M (2023). Machine learning and deep learning approach for medical image analysis: diagnosis to detection. Multimed Tools Appl.

[49] Jeyaraj PR, Samuel Nadar ER (2019). Computer-assisted medical image classification for early diagnosis of oral cancer employing deep learning algorithm. J Cancer Res Clin Oncol.

[50] Echle A, Rindtorff NT, Brinker TJ, Luedde T, Pearson AT, Kather JN (2021). Deep learning in cancer pathology: a new generation of clinical biomarkers. Br J Cancer.

[51] Savadjiev P, Chong J, Dohan A (2019). Image-based biomarkers for solid tumor quantification. Eur Radiol.

[52] Xu Y, Hosny A, Zeleznik R (2019). Deep learning predicts lung cancer treatment response from serial medical imaging. Clin Cancer Res.

[53] Ahishakiye E, Van Gijzen MB, Tumwiine J, Wario R, Obungoloch J (2021). A survey on deep learning in medical image reconstruction. Intelligent Medicine.

[54] Vamathevan J, Clark D, Czodrowski P (2019). Applications of machine learning in drug discovery and development. Nat Rev Drug Discov.

[55] Ginsburg GS, Willard HF (2009). Genomic and personalized medicine: foundations and applications. Transl Res.

[56] Chen YM, Hsiao TH, Lin CH, Fann YC (2025). Unlocking precision medicine: clinical applications of integrating health records, genetics, and immunology through artificial intelligence. J Biomed Sci.

[57] Gupta S, Janu N, Nawal M, Goswami A, Choudhary S, Kumar S, Gowroju S, Gulhane M, Sri Lakshmi R (2025). Genomics at the Nexus of AI, Computer Vision, and Machine Learning.

[58] Suissa JS, De La Cerda GY, Graber LC (2024). Data-driven guidelines for phylogenomic analyses using SNP data. Appl Plant Sci.

[59] Hollox EJ, Zuccherato LW, Tucci S (2022). Genome structural variation in human evolution. Trends Genet.

[60] Audic S, Claverie JM (1997). The significance of digital gene expression profiles. Genome Res.

[61] Dai W, Qiao X, Fang Y (2024). Epigenetics-targeted drugs: current paradigms and future challenges. Sig Transduct Target Ther.

[62] Schmitz MJ, Bashar A, Soman V (2025). Leveraging diverse genomic data to guide equitable carrier screening: Insights from gnomAD v.4.1.0. Am J Hum Genet.

[63] Gulamali FF, Sawant AS, Nadkarni GN (2022). Machine learning for risk stratification in kidney disease. Curr Opin Nephrol Hypertens.

[64] Jamalinia M, Weiskirchen R (2025). Advances in personalized medicine: translating genomic insights into targeted therapies for cancer treatment. Ann Transl Med.

[65] Mani S, Lalani SR, Pammi M (2025). Genomics and multiomics in the age of precision medicine. Pediatr Res.

[66] Vadapalli S, Abdelhalim H, Zeeshan S, Ahmed Z (2022). Artificial intelligence and machine learning approaches using gene expression and variant data for personalized medicine. Brief Bioinform.

[67] Quazi S (2022). Artificial intelligence and machine learning in precision and genomic medicine. Med Oncol.

[68] Khan M (2023). Bioinformatics and machine learning: analyzing genomic data for personalized medicine. Open Science Framework.

[69] Dara S, Dhamercherla S, Jadav SS, Babu CM, Ahsan MJ (2022). Machine learning in drug discovery: a review. Artif Intell Rev.

[70] Kearney E, Wojcik A, Babu D (2020). Artificial intelligence in genetic services delivery: utopia or apocalypse?. J Genet Couns.

[71] Benning L, Peintner A, Peintner L (2022). Advances in and the applicability of machine learning-based screening and early detection approaches for cancer: a primer. Cancers (Basel).

[72] Tao K, Bian Z, Zhang Q (2020). Machine learning-based genome-wide interrogation of somatic copy number aberrations in circulating tumor DNA for early detection of hepatocellular carcinoma. EBioMedicine.

[73] Mullen M, Zhang A, Lui GK, Romfh AW, Rhee JW, Wu JC (2021). Race and genetics in congenital heart disease: application of iPSCs, omics, and machine learning technologies. Front Cardiovasc Med.

[74] Cortés-Ciriano I, Gulhan DC, Lee JJK, Melloni GEM, Park PJ (2022). Computational analysis of cancer genome sequencing data. Nat Rev Genet.

[75] Dargan S, Kumar M (2020). A comprehensive survey on the biometric recognition systems based on physiological and behavioral modalities. Expert Syst Appl.

[76] Uwaechia AN, Ramli DA (2021). A comprehensive survey on ECG signals as new biometric modality for human authentication: recent advances and future challenges. IEEE Access.

[77] Paranjape RB, Mahovsky J, Benedicenti L, Koles’ Z The electroencephalogram as a biometric.

[78] Franceschetti L, Lodetti G, Blandino A, Amadasi A, Bugelli V (2024). Exploring the role of the human microbiome in forensic identification: opportunities and challenges. Int J Legal Med.

[79] Sharma A, Kumar R, Varadwaj P (2023). Smelling the disease: diagnostic potential of breath analysis. Mol Diagn Ther.

[80] Abdulrahman SA, Alhayani B (2023). A comprehensive survey on the biometric systems based on physiological and behavioural characteristics. Mater Today.

[81] Kisku DR, Gupta P, Sing JK (2019). Design and Implementation of Healthcare Biometric Systems.

[82] Mason J, Dave R, Chatterjee P, Graham-Allen I, Esterline A, Roy K (2020). An investigation of biometric authentication in the healthcare environment. Array.

[83] Kaul SD, Murty VK, Hatzinakos D Secure and privacy preserving biometric based user authentication with data access control system in the healthcare environment.

[84] Barka E, Al Baqari M, Kerrache CA, Herrera-Tapia J (2022). Implementation of a biometric-based blockchain system for preserving privacy, security, and access control in healthcare records. J Sens Actuator Netw.

[85] Choi J h., Khamraev K, Cheriyan D (2022). Hybrid health risk assessment model using real-time particulate matter, biometrics, and benchmark device. J Clean Prod.

[86] Baseri Y, Hafid A, Firoozjaei MD, Cherkaoui S, Ray I (2024). Statistical privacy protection for secure data access control in cloud. J Inf Secur Appl.

[87] Riplinger L, Piera-Jiménez J, Dooling JP (2020). Patient identification techniques - approaches, implications, and findings. Yearb Med Inform.

[88] Sohn JW, Kim H, Park SB (2020). Clinical study of using biometrics to identify patient and procedure. Front Oncol.

[89] Fatimah B, Singh P, Singhal A, Pachori RB (2022). Biometric identification from ECG signals using Fourier decomposition and machine learning. IEEE Trans Instrum Meas.

[90] Prakash AJ, Patro KK, Samantray S, Pławiak P, Hammad M (2023). A deep learning technique for biometric authentication using ECG beat template matching. Information.

[91] Mohsin AH, Zaidan AA, Zaidan BB (2018). Real-time remote health monitoring systems using body sensor information and finger vein biometric verification: a multi-layer systematic review. J Med Syst.

[92] Ismail SNA, Nayan NA, Jaafar R, May Z (2022). Recent advances in non-invasive blood pressure monitoring and prediction using a machine learning approach. Sensors (Basel).

[93] Fei C, Liu R, Li Z, Wang T, Baig FN, Manocha AK, Jain S, Singh M, Paul S (2021). Computational Intelligence in Healthcare Health Information Science.

[94] Gomes N, Pato M, Lourenço AR, Datia N (2023). A survey on wearable sensors for mental health monitoring. Sensors (Basel).

[95] Killoran J, Cui Y (Gina, Park A, van Esch P, Kietzmann J (2023). Can behavioral biometrics make everyone happy?. Bus Horiz.

[96] Garcia-Ceja E, Riegler M, Nordgreen T, Jakobsen P, Oedegaard KJ, Tørresen J (2018). Mental health monitoring with multimodal sensing and machine learning: a survey. Pervasive Mob Comput.

[97] Harrer S, Shah P, Antony B, Hu J (2019). Artificial intelligence for clinical trial design. Trends Pharmacol Sci.

[98] Weissler EH, Naumann T, Andersson T (2021). The role of machine learning in clinical research: transforming the future of evidence generation. Trials.

[99] Moro-Velazquez L, Gomez-Garcia JA, Arias-Londoño JD, Dehak N, Godino-Llorente JI (2021). Advances in Parkinson’s disease detection and assessment using voice and speech: a review of the articulatory and phonatory aspects. Biomed Signal Process Control.

[100] Ngo QC, Motin MA, Pah ND, Drotár P, Kempster P, Kumar D (2022). Computerized analysis of speech and voice for Parkinson’s disease: a systematic review. Comput Methods Programs Biomed.

[101] Lella KK, Pja A (2021). Automatic COVID-19 disease diagnosis using 1D convolutional neural network and augmentation with human respiratory sound based on parameters: cough, breath, and voice. AIMS Public Health.

[102] Idrisoglu A, Dallora AL, Anderberg P, Berglund JS (2023). Applied machine learning techniques to diagnose voice-affecting conditions and disorders: systematic literature review. J Med Internet Res.

[103] Lyakso E, Frolova O, Nikolaev A (2021). Psychological Applications and Trends.

[104] Onyema EM, Shukla PK, Dalal S, Mathur MN, Zakariah M, Tiwari B (2021). Enhancement of patient facial recognition through deep learning algorithm: ConvNet. J Healthc Eng.

[105] Jeon B, Jeong B, Jee S (2019). A facial recognition mobile app for patient safety and biometric identification: design, development, and validation. JMIR Mhealth Uhealth.

[106] Ghazal TM, Hasan MK, Alshurideh MT (2021). IoT for smart cities: machine learning approaches in smart healthcare—a review. Future Internet.

[107] Balakrishna S, Thirumaran M, Solanki VK, Balas V, Solanki V, Kumar R, Ahad M (2020). A Handbook of Internet of Things in Biomedical and Cyber Physical System Intelligent Systems Reference Library.

[108] Lv Z, Li Y (2022). Wearable sensors for vital signs measurement: a survey. J Sens Actuator Netw.

[109] Junaid SB, Imam AA, Shuaibu AN (2022). Artificial intelligence, sensors and vital health signs: a review. Appl Sci (Basel).

[110] Gupta N, Gupta SK, Pathak RK, Jain V, Rashidi P, Suri JS (2022). Human activity recognition in artificial intelligence framework: a narrative review. Artif Intell Rev.

[111] Zhu T, Uduku C, Li K, Herrero P, Oliver N, Georgiou P (2022). Enhancing self-management in type 1 diabetes with wearables and deep learning. NPJ Digit Med.

[112] Liyakat KKS, Shaik A (2025). AI-Powered Advances in Pharmacology.

[113] Kwon SH, Dong L (2022). Flexible sensors and machine learning for heart monitoring. Nano Energy.

[114] Lin SY, Tsai CY, Majumdar A (2024). Combining a wireless radar sleep monitoring device with deep machine learning techniques to assess obstructive sleep apnea severity. J Clin Sleep Med.

[115] Arora A, Chakraborty P, Bhatia MPS (2020). Analysis of data from wearable sensors for sleep quality estimation and prediction using deep learning. Arab J Sci Eng.

[116] Zhang W, Ram S (2020). A comprehensive analysis of triggers and risk factors for asthma based on machine learning and large heterogeneous data sources. MIS Q.

[117] Bohlmann A, Mostafa J, Kumar M (2021). Machine learning and medication adherence: scoping review. JMIRx Med.

[118] Roh H, Shin S, Han J, Lim S (2021). A deep learning-based medication behavior monitoring system. Math Biosci Eng.

[119] Meyer BM, Tulipani LJ, Gurchiek RD (2020). Wearables and deep learning classify fall risk from gait in multiple sclerosis. IEEE J Biomed Health Inform.

[120] Kyamakya K, Al-Machot F, Haj Mosa A, Bouchachia H, Chedjou JC, Bagula A (2021). Emotion and stress recognition related sensors and machine learning technologies. Sensors (Basel).

[121] Gedam S, Paul S (2021). A review on mental stress detection using wearable sensors and machine learning techniques. IEEE Access.

[122] Fountzilas E, Pearce T, Baysal MA, Chakraborty A, Tsimberidou AM (2025). Convergence of evolving artificial intelligence and machine learning techniques in precision oncology. NPJ Digit Med.

[123] Acosta JN, Falcone GJ, Rajpurkar P, Topol EJ (2022). Multimodal biomedical AI. Nat Med.

[124] Hsueh PYS, Dey S, Das S, Wetter T, Gundlapalli AV, Jaulent MC, Zhao D (2017). MEDINFO 2017: Precision Healthcare through Informatics.

[125] Mendo IR, Marques G, de la Torre Díez I, López-Coronado M, Martín-Rodríguez F (2021). Machine learning in medical emergencies: a systematic review and analysis. J Med Syst.

[126] van der Boon RMA, Camm AJ, Aguiar C (2024). Risks and benefits of sharing patient information on social media: a digital dilemma. Eur Heart J Digit Health.

[127] Gupta A, Katarya R (2020). Social media based surveillance systems for healthcare using machine learning: a systematic review. J Biomed Inform.

[128] Hasib KM, Islam MR, Sakib S, Akbar MA, Razzak I, Alam MS (2023). Depression detection from social networks data based on machine learning and deep learning techniques: an interrogative survey. IEEE Trans Comput Soc Syst.

[129] Johnson KB, Wei WQ, Weeraratne D (2021). Precision medicine, AI, and the future of personalized health care. Clin Transl Sci.

[130] Verma S, Malviya R, Alam MA, Tripathi BD, Malviya R, Ghinea G, Dhanaraj RK, Balusamy B, Sundram S (2022). Deep Learning for Targeted Treatments: Transformation in Healthcare.

[131] Moorthy V, Abubakar I, Qadri F (2024). The future of the global clinical trial ecosystem: a vision from the first WHO Global Clinical Trials Forum. The Lancet.

[132] Pettit RW, Fullem R, Cheng C, Amos CI (2021). Artificial intelligence, machine learning, and deep learning for clinical outcome prediction. Emerg Top Life Sci.

[133] Cohen IG (2019). Informed consent and medical artificial intelligence: what to tell the patient?. SSRN Journal.

[134] McKeown A, Mourby M, Harrison P, Walker S, Sheehan M, Singh I (2021). Ethical issues in consent for the reuse of data in health data platforms. Sci Eng Ethics.

[135] Chien I, Enrique A, Palacios J (2020). A machine learning approach to understanding patterns of engagement with internet-delivered mental health interventions. JAMA Netw Open.

[136] Benke K, Benke G (2018). Artificial intelligence and big data in public health. Int J Environ Res Public Health.

[137] Song L, Li Y, Nie S (2023). Using machine learning to predict adverse events in acute coronary syndrome: a retrospective study. Clin Cardiol.

[138] Yang J, Wan J, Feng L (2024). Machine learning algorithms for the prediction of adverse prognosis in patients undergoing peritoneal dialysis. BMC Med Inform Decis Mak.

[139] Badwan BA, Liaropoulos G, Kyrodimos E, Skaltsas D, Tsirigos A, Gorgoulis VG (2023). Machine learning approaches to predict drug efficacy and toxicity in oncology. Cell Rep Methods.

[140] Feijoo F, Palopoli M, Bernstein J, Siddiqui S, Albright TE (2020). Key indicators of phase transition for clinical trials through machine learning. Drug Discov Today.

[141] MacEachern SJ, Forkert ND (2021). Machine learning for precision medicine. Genome.

[142] Chalasani SH, Syed J, Ramesh M, Patil V, Pramod Kumar TM (2023). Artificial intelligence in the field of pharmacy practice: a literature review. Explor Res Clin Soc Pharm.

[143] Askr H, Elgeldawi E, Aboul Ella H, Elshaier Y, Gomaa MM, Hassanien AE (2023). Deep learning in drug discovery: an integrative review and future challenges. Artif Intell Rev.

[144] Li Q, Tang B, Wu Y (2024). Machine learning: a new approach for dose individualization. Clin Pharma and Therapeutics.

[145] Del Fabro L, Bondi E, Serio F, Maggioni E, D’Agostino A, Brambilla P (2023). Machine learning methods to predict outcomes of pharmacological treatment in psychosis. Transl Psychiatry.

[146] D’Costa A, Zatale A (2021). AI and the cardiologist: when mind, heart and machine unite. Open Heart.

[147] Hasan MM, Young GJ, Shi J (2021). A machine learning based two-stage clinical decision support system for predicting patients’ discontinuation from opioid use disorder treatment: retrospective observational study. BMC Med Inform Decis Mak.

[148] Kim HR, Sung M, Park JA (2022). Analyzing adverse drug reaction using statistical and machine learning methods: a systematic review. Medicine (Baltimore).

[149] Zhang S, Bamakan SMH, Qu Q, Li S (2019). Learning for personalized medicine: a comprehensive review from a deep learning perspective. IEEE Rev Biomed Eng.

[150] Meng W, Zhang X, Ru B, Guan Y (2023). A machine learning approach to real‐world time to treatment discontinuation prediction. Advanced Intelligent Systems.

[151] Gu Y, Zalkikar A, Liu M (2021). Predicting medication adherence using ensemble learning and deep learning models with large scale healthcare data. Sci Rep.

[152] Khan S, Sajjad M, Hussain T, Ullah A, Imran AS (2020). A review on traditional machine learning and deep learning models for WBCs classification in blood smear images. IEEE Access.

[153] De Bruyne S, De Kesel P, Oyaert M (2023). Applications of artificial intelligence in urinalysis: is the future already here?. Clin Chem.

[154] Goodswen SJ, Barratt JLN, Kennedy PJ, Kaufer A, Calarco L, Ellis JT (2021). Machine learning and applications in microbiology. FEMS Microbiol Rev.

[155] Ghannam RB, Techtmann SM (2021). Machine learning applications in microbial ecology, human microbiome studies, and environmental monitoring. Comput Struct Biotechnol J.

[156] Unger M, Kather JN (2024). Deep learning in cancer genomics and histopathology. Genome Med.

[157] Obstfeld AE (2023). Hematology and machine learning. J Appl Lab Med.

[158] Danieli MG, Brunetto S, Gammeri L (2024). Machine learning application in autoimmune diseases: state of art and future prospectives. Autoimmun Rev.

[159] Usategui I, Barbado J, Torres AM, Cascón J, Mateo J (2023). Machine learning, a new tool for the detection of immunodeficiency patterns in systemic lupus erythematosus. J Investig Med.

[160] Thomasian NM, Kamel IR, Bai HX (2022). Machine intelligence in non-invasive endocrine cancer diagnostics. Nat Rev Endocrinol.

[161] Hong N, Park H, Rhee Y (2020). Machine learning applications in endocrinology and metabolism research: an overview. Endocrinol Metab.

[162] Amjad A, Kordel P, Fernandes G (2023). A review on innovation in healthcare sector (telehealth) through artificial intelligence. Sustainability.

[163] Gajarawala SN, Pelkowski JN (2021). Telehealth benefits and barriers. J Nurse Pract.

[164] Schünke LC, Mello B, da Costa CA (2022). A rapid review of machine learning approaches for telemedicine in the scope of COVID-19. Artif Intell Med.

[165] Hou Y, Huang J (2025). Natural language processing for social science research: a comprehensive review. Chin J Sociol.

[166] Hughes A, Shandhi MMH, Master H, Dunn J, Brittain E (2023). Wearable devices in cardiovascular medicine. Circ Res.

[167] Liu Y, Wang B (2025). Advanced applications in chronic disease monitoring using IoT mobile sensing device data, machine learning algorithms and frame theory: a systematic review. Front Public Health.

[168] Segal G, Segev A, Brom A, Lifshitz Y, Wasserstrum Y, Zimlichman E (2019). Reducing drug prescription errors and adverse drug events by application of a probabilistic, machine-learning based clinical decision support system in an inpatient setting. J Am Med Inform Assoc.

[169] Verma D, Bach K, Mork PJ (2021). Application of machine learning methods on patient reported outcome measurements for predicting outcomes: a literature review. Informatics (MDPI).

[170] Björneld O, Carlsson M, Löwe W (2023). Case study - Feature engineering inspired by domain experts on real world medical data. Intelligence-Based Medicine.

[171] Xu X, Li J, Zhu Z (2024). A comprehensive review on synergy of multi-modal data and AI technologies in medical diagnosis. Bioengineering (Basel).

[172] Zhou SK, Greenspan H, Davatzikos C (2021). A review of deep learning in medical imaging: imaging traits, technology trends, case studies with progress highlights, and future promises. Proc IEEE.

[173] Khalifa M, Albadawy M (2024). AI in diagnostic imaging: revolutionising accuracy and efficiency. Computer Methods and Programs in Biomedicine Update.

[174] Aggarwal R, Sounderajah V, Martin G (2021). Diagnostic accuracy of deep learning in medical imaging: a systematic review and meta-analysis. NPJ Digit Med.

[175] Prasad VK, Verma A, Bhattacharya P (2024). Revolutionizing healthcare: a comparative insight into deep learning’s role in medical imaging. Sci Rep.

[176] Zhang K, Yang X, Wang Y (2025). Artificial intelligence in drug development. Nat Med.

[177] Kant S, Roy S (2025). Artificial intelligence in drug discovery and development: transforming challenges into opportunities. Discov Pharm Sci.

[178] Gomes B, Ashley EA (2023). Artificial intelligence in molecular medicine. N Engl J Med.

[179] Davis S, Zhang J, Lee I (2022). Effective hospital readmission prediction models using machine-learned features. BMC Health Serv Res.

[180] Dixon D, Sattar H, Moros N (2024). Unveiling the influence of AI predictive analytics on patient outcomes: a comprehensive narrative review. Cureus.

[181] Oh EG, Oh S, Cho S, Moon M (2025). Predicting readmission among high-risk discharged patients using a machine learning model with nursing data: retrospective study. JMIR Med Inform.

[182] Varghese C, Harrison EM, O’Grady G, Topol EJ (2024). Artificial intelligence in surgery. Nat Med.

[183] Antel R, Abbasgholizadeh-Rahimi S, Guadagno E, Harley JM, Poenaru D (2022). The use of artificial intelligence and virtual reality in doctor-patient risk communication: a scoping review. Patient Educ Couns.

[184] Wah JNK (2025). Revolutionizing e-health: the transformative role of AI-powered hybrid chatbots in healthcare solutions. Front Public Health.

[185] Alowais SA, Alghamdi SS, Alsuhebany N (2023). Revolutionizing healthcare: the role of artificial intelligence in clinical practice. BMC Med Educ.

[186] Davenport T, Kalakota R (2019). The potential for artificial intelligence in healthcare. Future Healthc J.

[187] Bravo F, Braun M, Farias V (2021). Optimization-driven framework to understand health care network costs and resource allocation. Health Care Manag Sci.

[188] Wong F, de la Fuente-Nunez C, Collins JJ (2023). Leveraging artificial intelligence in the fight against infectious diseases. Science.

[189] Alimadadi A, Aryal S, Manandhar I, Munroe PB, Joe B, Cheng X (2020). Artificial intelligence and machine learning to fight COVID-19. Physiol Genomics.

[190] Bhardhwaj HS, Tutika S, Kaushik L.S. S, Gupta PK, Reddy K.S. T, Zarrintaj P, Yazdi MK, Bencherif SA, Saeb MR, Mozafari M (2026). Artificial Intelligence in Biomaterials Design and Development.

[191] Wang L, Zhang Y, Wang D (2021). Artificial intelligence for COVID-19: a systematic review. Front Med.

[192] Lo Vercio L, Amador K, Bannister JJ (2020). Supervised machine learning tools: a tutorial for clinicians. J Neural Eng.

[193] Erickson BJ, Korfiatis P, Akkus Z, Kline TL (2017). Machine learning for medical imaging. Radiographics.

[194] Kukreja S, Kumar A, Khan GA, Dagur A, Singh K, Mehra PS, Shukla DK (2023). Artificial Intelligence, Blockchain, Computing and Security.

[195] Bertsimas D, Wiberg H (2020). Machine learning in oncology: methods, applications, and challenges. JCO Clin Cancer Inform.

[196] Asri H, Mousannif H, Moatassime HA, Noel T (2016). Using machine learning algorithms for breast cancer risk prediction and diagnosis. Procedia Comput Sci.

[197] Talwar A, Lopez-Olivo MA, Huang Y, Ying L, Aparasu RR (2023). Performance of advanced machine learning algorithms overlogistic regression in predicting hospital readmissions: a meta-analysis. Explor Res Clin Soc Pharm.

[198] Colace F, Gupta BB, Lorusso A, Troiano A, Santaniello D, Valentino C, Gupta BB, Colace F (2024). Handbook of Research on AI and ML for Intelligent Machines and Systems.

[199] Makino M, Shimizu K, Kadota K (2024). Enhanced clustering-based differential expression analysis method for RNA-seq data. MethodsX.

[200] Kauffman J, Miotto R, Klang E (2025). Embedding methods for electronic health record research. Annu Rev Biomed Data Sci.

[201] Wu J, Cui Y, Sun X (2017). Unsupervised clustering of quantitative image phenotypes reveals breast cancer subtypes with distinct prognoses and molecular pathways. Clin Cancer Res.

[202] Ringnér M (2008). What is principal component analysis?. Nat Biotechnol.

[203] van der Maaten L, Hinton G (2008). Visualizing data using t-SNE. J Mach Learn Res.

[204] Smith SM (2002). Fast robust automated brain extraction. Hum Brain Mapp.

[205] Menze BH, Jakab A, Bauer S (2015). The multimodal brain tumor image segmentation benchmark (BRATS). IEEE Trans Med Imaging.

[206] Qiu L, Cheng J, Gao H, Xiong W, Ren H (2023). Federated semi-supervised learning for medical image segmentation via pseudo-label denoising. IEEE J Biomed Health Inform.

[207] Yang X, Song Z, King I, Xu Z (2022). A survey on deep semi-supervised learning. IEEE Trans Knowl Data Eng.

[208] Eckardt JN, Bornhäuser M, Wendt K, Middeke JM (2022). Semi-supervised learning in cancer diagnostics. Front Oncol.

[209] Jiao R, Zhang Y, Ding L (2024). Learning with limited annotations: a survey on deep semi-supervised learning for medical image segmentation. Comput Biol Med.

[210] Yu C, Liu J, Nemati S, Yin G (2023). Reinforcement learning in healthcare: a survey. ACM Comput Surv.

[211] Komorowski M, Celi LA, Badawi O, Gordon AC, Faisal AA (2018). The artificial intelligence clinician learns optimal treatment strategies for sepsis in intensive care. Nat Med.

[212] Chi W (2019). Context-aware learning for robot-assisted endovascular catheterization [Dissertation]. https://spiral.imperial.ac.uk/entities/publication/17ec29f1-20bb-4171-95c5-7249267a5478.

[213] Wang Y, Zhao Y, Petzold L Predicting the need for blood transfusion in intensive care units with reinforcement learning.

[214] McLaverty B (2023). Unifying data-driven modeling with machine learning to improve personalized treatment of critical care patients [Dissertation]. https://d-scholarship.pitt.edu/concern/etds/1325d248-7852-4eba-aa0e-0a43f2fa4243.

[215] Almagrabi AO, Ali R, Alghazzawi D, AlBarakati A, Khurshaid T (2022). A reinforcement learning-based framework for crowdsourcing in massive health care Internet of Things. Big Data.

[216] Rashidi HH, Albahra S, Robertson S, Tran NK, Hu B (2023). Common statistical concepts in the supervised machine learning arena. Front Oncol.

[217] Esteva A, Kuprel B, Novoa RA (2017). Dermatologist-level classification of skin cancer with deep neural networks. Nature New Biol.

[218] Shah D, Patel S, Bharti SK (2020). Heart disease prediction using machine learning techniques. SN Comput Sci.

[219] Levin S, Toerper M, Hamrock E (2018). Machine-learning-based electronic triage more accurately differentiates patients with respect to clinical outcomes compared with the emergency severity index. Ann Emerg Med.

[220] Zou Q, Qu K, Luo Y, Yin D, Ju Y, Tang H (2018). Predicting diabetes mellitus with machine learning techniques. Front Genet.

[221] Mucaki EJ, Zhao JZL, Lizotte DJ, Rogan PK (2019). Predicting responses to platin chemotherapy agents with biochemically-inspired machine learning. Signal Transduct Target Ther.

[222] Battineni G, Sagaro GG, Chinatalapudi N, Amenta F (2020). Applications of machine learning predictive models in the chronic disease diagnosis. J Pers Med.

[223] Dhiman P, Ma J, Andaur Navarro CL (2022). Methodological conduct of prognostic prediction models developed using machine learning in oncology: a systematic review. BMC Med Res Methodol.

[224] Palomino-Echeverria S, Huergo E, Ortega-Legarreta A (2024). A robust clustering strategy for stratification unveils unique patient subgroups in acutely decompensated cirrhosis. J Transl Med.

[225] Sinha A, Aljrees T, Pandey SK (2023). Semi-supervised clustering-based DANA algorithm for data gathering and disease detection in healthcare wireless sensor networks (WSN). Sensors (Basel).

[226] Ren Z, Yeh RA, Schwing AG Not all unlabeled data are equal: learning to weight data in semi-supervised learning.

[227] Huang L (2023). Combination of information in labeled and unlabeled data via evidence theory. IEEE Trans Artif Intell.

[228] Qiu S, Chen Y, Yang Y (2024). A review on semi-supervised learning for EEG-based emotion recognition. Information Fusion.

[229] Christopoulou SC (2024). Machine learning models and technologies for evidence-based telehealth and smart care: a review. BioMedInformatics.

[230] Naeem M, Rizvi STH, Coronato A (2020). A gentle introduction to reinforcement learning and its application in different fields. IEEE Access.

[231] Sverdlov O, Ryeznik Y, Wong WK (2021). Opportunity for efficiency in clinical development: an overview of adaptive clinical trial designs and innovative machine learning tools, with examples from the cardiovascular field. Contemp Clin Trials.

[232] Kumar CUO, Singh I, Suguna M (2024). Optimizing patient recruitment for clinical trials: a hybrid classification model and game-theoretic approach for strategic interaction. IEEE Access.

[233] Malheiro V, Santos B, Figueiras A, Mascarenhas-Melo F (2025). The potential of artificial intelligence in pharmaceutical innovation: from drug discovery to clinical trials. Pharmaceuticals (Basel).

[234] McKinney SM, Sieniek M, Godbole V (2020). International evaluation of an AI system for breast cancer screening. Nature New Biol.

[235] Zhavoronkov A, Ivanenkov YA, Aliper A (2019). Deep learning enables rapid identification of potent DDR1 kinase inhibitors. Nat Biotechnol.

[236] Adams R, Henry KE, Sridharan A (2022). Prospective, multi-site study of patient outcomes after implementation of the TREWS machine learning-based early warning system for sepsis. Nat Med.

[237] Piening B, Bapat B, Weerasinghe RK (2023). Improved outcomes from reflex comprehensive genomic profiling-guided precision therapeutic selection across a major US healthcare system. JCO.

[238] Iyortsuun NK, Kim SH, Jhon M, Yang HJ, Pant S (2023). A review of machine learning and deep learning approaches on mental health diagnosis. Healthcare (Basel).

[239] Konečný J, McMahan HB, Ramage D, Richtárik P (2016). Federated optimization: distributed machine learning for on-device intelligence. arXiv.

[240] Yang Q, Liu Y, Chen T, Tong Y (2019). Federated machine learning: concept and applications. ACM Transactions on Intelligent Systems and Technology.

[241] Zhang X, Mavromatis A, Vafeas A, Nejabati R, Simeonidou D (2023). Federated feature selection for horizontal federated learning in IoT networks. IEEE Internet Things J.

[242] Gao D, Ju C, Wei X, Liu Y, Chen T, Yang Q (2019). HHHFL: hierarchical heterogeneous horizontal federated learning for electroencephalography. arXiv.

[243] Huang W, Li T, Wang D, Du S, Zhang J, Huang T (2022). Fairness and accuracy in horizontal federated learning. Inf Sci (Ny).

[244] Liu Y, Kang Y, Zou T (2024). Vertical federated learning: concepts, advances, and challenges. IEEE Trans Knowl Data Eng.

[245] Feng S, Yu H, Zhu Y (2024). MMVFL: a simple vertical federated learning framework for multi-class multi-participant scenarios. Sensors (Basel).

[246] Gupta M, Sharma P, Kalra R, Hassan A, Prasad VK, Bhattacharya P, Dutta P, Damaševičius R (2024). Federated Learning and AI for Healthcare 5.0.

[247] Liu Y, Kang Y, Xing C, Chen T, Yang Q (2020). A secure federated transfer learning framework. IEEE Intell Syst.

[248] Chen Y, Qin X, Wang J, Yu C, Gao W (2020). FedHealth: a federated transfer learning framework for wearable healthcare. IEEE Intell Syst.

[249] Chorney W, Wang H (2024). Towards federated transfer learning in electrocardiogram signal analysis. Comput Biol Med.

[250] Rieke N, Hancox J, Li W (2020). The future of digital health with federated learning. NPJ Digit Med.

[251] Nguyen DC, Pham QV, Pathirana PN (2023). Federated learning for smart healthcare: a survey. ACM Comput Surv.

[252] Li T, Sahu AK, Talwalkar A, Smith V (2020). Federated learning: challenges, methods, and future directions. IEEE Signal Process Mag.

[253] Heyndrickx W, Mervin L, Morawietz T (2024). MELLODDY: cross-pharma federated learning at unprecedented scale unlocks benefits in QSAR without compromising proprietary information. J Chem Inf Model.

[254] Foley P, Sheller MJ, Edwards B (2022). OpenFL: the open federated learning library. Phys Med Biol.

[255] Mora A, Bujari A, Bellavista P (2024). Enhancing generalization in federated learning with heterogeneous data: a comparative literature review. Future Generation Computer Systems.

[256] Almanifi ORA, Chow CO, Tham ML, Chuah JH, Kanesan J (2023). Communication and computation efficiency in federated learning: a survey. Internet of Things.

[257] Dhatterwal JS, Malik K, Kaswan KS, Elngar AA, Elngar AA, Oliva D, Balas VE (2024). Artificial Intelligence Using Federated Learning.

[258] Mohammadi S, Balador A, Sinaei S, Flammini F (2024). Balancing privacy and performance in federated learning: a systematic literature review on methods and metrics. J Parallel Distrib Comput.

[259] Saha S, Hota A, Chattopadhyay AK, Nag A, Nandi S (2024). A multifaceted survey on privacy preservation of federated learning: progress, challenges, and opportunities. Artif Intell Rev.

[260] Narula M, Meena J, Vishwakarma DK (2024). A comprehensive review on federated learning for data-sensitive application: open issues & challenges. Eng Appl Artif Intell.

[261] Malik H, Anees T (2024). Federated learning with deep convolutional neural networks for the detection of multiple chest diseases using chest x-rays. Multimed Tools Appl.

[262] Dasaradharami Reddy K, Gadekallu TR (2023). A comprehensive survey on federated learning techniques for healthcare informatics. Comput Intell Neurosci.

[263] Kaissis GA, Makowski MR, Rückert D, Braren RF (2020). Secure, privacy-preserving and federated machine learning in medical imaging. Nat Mach Intell.

[264] Rafi TH, Noor FA, Hussain T, Chae DK (2024). Fairness and privacy preserving in federated learning: a survey. Information Fusion.

[265] Djebrouni Y, Benarba N, Touat O (2023). Bias mitigation in federated learning for edge computing. Proc ACM Interact Mob Wearable Ubiquitous Technol.

[266] You L, Guo Z, Zuo B, Chang Y, Yuen C (2024). SLMFed: a stage-based and layerwise mechanism for incremental federated learning to assist dynamic and ubiquitous IoT. IEEE Internet Things J.

[267] Mazzocca C, Romandini N, Montanari R, Bellavista P (2024). Enabling federated learning at the edge through the IOTA tangle. Future Generation Computer Systems.

[268] Ji J, Shu Z, Li H (2024). Edge-computing-based knowledge distillation and multitask learning for partial discharge recognition. IEEE Trans Instrum Meas.

[269] Elhattab F, Bouchenak S, Boscher C (2024). PASTEL: privacy-preserving federated learning in edge computing. Proc ACM Interact Mob Wearable Ubiquitous Technol.

[270] Nakamoto S (2008). Bitcoin: a peer-to-peer electronic cash system. https://bitcoin.org/bitcoin.pdf.

[271] Wood G (2025). Ethereum: a secure decentralised generalised transaction ledger. https://ethereum.github.io/yellowpaper/paper.pdf.

[272] Dutta P, Choi TM, Somani S, Butala R (2020). Blockchain technology in supply chain operations: applications, challenges and research opportunities. Transp Res E Logist Transp Rev.

[273] Casado-Vara R, Prieto J, la Prieta FD, Corchado JM (2018). How blockchain improves the supply chain: case study alimentary supply chain. Procedia Comput Sci.

[274] Korpela K, Hallikas J, Dahlberg T Digital supply chain transformation toward blockchain integration.

[275] Queiroz MM, Telles R, Bonilla SH (2019). Blockchain and supply chain management integration: a systematic review of the literature. Supply Chain Management.

[276] Raval S (2016). Decentralized Applications: Harnessing Bitcoin’s Blockchain Technology.

[277] Joshi AP, Han M, Wang Y (2018). A survey on security and privacy issues of blockchain technology. Mathematical Foundations of Computing.

[278] Mohanta BK, Jena D, Panda SS, Sobhanayak S (2019). Blockchain technology: a survey on applications and security privacy challenges. Internet of Things.

[279] Ul Hassan M, Rehmani MH, Chen J (2022). Anomaly detection in blockchain networks: a comprehensive survey. IEEE Commun Surv Tutorials.

[280] Shahsavari Y, Zhang K, Talhi C (2020). A theoretical model for block propagation analysis in bitcoin network. IEEE Trans Eng Manage.

[281] Belchior R, Vasconcelos A, Guerreiro S, Correia M (2022). A survey on blockchain interoperability: past, present, and future trends. ACM Comput Surv.

[282] Lafourcade P, Lombard-Platet M (2020). About blockchain interoperability. Inf Process Lett.

[283] Schulte S, Sigwart M, Frauenthaler P, Borkowski M, Di Ciccio C (2019). Business Process Management: Blockchain and Central and Eastern Europe Forum BPM 2019 Lecture Notes in Business Information Processing.

[284] Hardjono T, Lipton A, Pentland A (2019). Toward an interoperability architecture for blockchain autonomous systems. IEEE Trans Eng Manage.

[285] Zhang P, White J, Schmidt DC, Lenz G (2017). Applying software patterns to address interoperability in blockchainbased healthcare apps. arXiv.

[286] Qasse IA, Abu Talib M, Nasir Q Inter blockchain communication.

[287] Zou W, Lo D, Kochhar PS (2019). Smart contract development: challenges and opportunities. IIEEE Trans Software Eng.

[288] Delmolino K, Arnett M, Kosba A, Miller A, Shi E, Clark J, Meiklejohn S, Ryan P, Wallach D, Brenner M, Rohloff K (2016). Financial Cryptography and Data Security FC 2016 Lecture Notes in Computer Science.

[289] Xu J, Wang C, Jia X (2023). A survey of blockchain consensus protocols. ACM Comput Surv.

[290] Nguyen GT, Kim K (2018). A survey about consensus algorithms used in blockchain. J Inf Process Syst.

[291] Monrat AA, Schelen O, Andersson K (2019). A survey of blockchain from the perspectives of applications, challenges, and opportunities. IEEE Access.

[292] Gervais A, Karame GO, Wüst K, Glykantzis V, Ritzdorf H, Capkun S On the security and performance of proof of work blockchains.

[293] Shahsavari Y, Zhang K, Talhi C A theoretical model for fork analysis in the bitcoin network.

[294] Nguyen CT, Hoang DT, Nguyen DN, Niyato D, Nguyen HT, Dutkiewicz E (2019). Proof-of-stake consensus mechanisms for future blockchain networks: fundamentals, applications and opportunities. IEEE Access.

[295] Buterin V (2014). A next-generation smart contract and decentralized application platform. https://cryptorating.eu/whitepapers/Ethereum/Ethereum_white_paper.pdf.

[296] Introduction. Cardano Documentation.

[297] Gilad Y, Hemo R, Micali S, Vlachos G, Zeldovich N Algorand: scaling byzantine agreements for cryptocurrencies.

[298] Wang Q, Xu M, Li X, Qian H Revisiting the fairness and randomness of delegated proof of stake consensus algorithm.

[299] Lamport L, Shostak R, Pease M, Malkhi D (2019). Concurrency: The Works of Leslie Lamport.

[300] Zhong W, Yang C, Liang W (2023). Byzantine fault-tolerant consensus algorithms: a survey. Electronics (Basel).

[301] Shahsavari Y, Zhang K, Talhi C Performance modeling and analysis of hotstuff for blockchain consensus.

[302] Androulaki E, Barger A, Bortnikov V Hyperledger fabric: a distributed operating system for permissioned blockchains.

[303] Li Y, Cao B, Peng M (2020). Direct acyclic graph-based ledger for Internet of Things: performance and security analysis. IEEE/ACM Trans Networking.

[304] Popov S (2018). The tangle. Semantic Scholar.

[305] Saa O, Cullen A, Vigneri L (2023). IOTA 2.0 incentives and tokenomics whitepaper. https://files.iota.org/papers/IOTA_2.0_Incentives_And_Tokenomics_Whitepaper.pdf.

[306] Pass R, Shi E (2016). Hybrid consensus: efficient consensus in the permissionless model. Cryptology ePrint Archive.

[307] Chepurnoy A, Duong T, Fan L, Zhou HS (2017). Twinscoin: a cryptocurrency via proof-of-work and proof-of-stake. Cryptology ePrint Archive.

[308] Kwon J (2014). Tendermint: consensus without mining. https://tendermint.com/static/docs/tendermint.pdf.

[309] Cheng Z, Wu G, Wu H, Zhao M, Zhao L, Cai Q (2018). A new hybrid consensus protocol: deterministic proof of work. arXiv.

[310] Jayakumari B, Sheeba SL, Eapen M (2024). E-voting system using cloud-based hybrid blockchain technology. J Saf Sci Resil.

[311] Zarrin J, Wen Phang H, Babu Saheer L, Zarrin B (2021). Blockchain for decentralization of internet: prospects, trends, and challenges. Cluster Comput.

[312] Dinh TTA, Liu R, Zhang M, Chen G, Ooi BC, Wang J (2018). Untangling blockchain: a data processing view of blockchain systems. IEEE Trans Knowl Data Eng.

[313] Decker C, Wattenhofer R Information propagation in the bitcoin network.

[314] Kan L, Wei Y, Hafiz Muhammad A, Siyuan W, Gao LC, Kai H A multiple blockchains architecture on inter-blockchain communication.

[315] Chen Z dong, Yu Z, Duan Z bo, Hu K (2017). Inter-blockchain communication. DEStech Transactions on Computer Science and Engineering.

[316] Tam Vo H, Wang Z, Karunamoorthy D, Wagner J, Abebe E, Mohania M Internet of blockchains: techniques and challenges ahead.

[317] Kwon J, Buchman E Cosmos: a network of distributed ledgers. GitHub.

[318] Wood G (2016). Polkadot: vision for a heterogeneous multi-chain framework. https://assets.polkadot.network/Polkadot-whitepaper.pdf.

[319] Wanchain.

[320] Mahajan HB, Junnarkar AA (2023). Smart healthcare system using integrated and lightweight ECC with private blockchain for multimedia medical data processing. Multimed Tools Appl.

[321] Pahlajani S, Kshirsagar A, Pachghare V Survey on private blockchain consensus algorithms.

[322] Antwi M, Adnane A, Ahmad F, Hussain R, Habib ur Rehman M, Kerrache CA (2021). The case of HyperLedger fabric as a blockchain solution for healthcare applications. Blockchain: Research and Applications.

[323] Al-Sumaidaee G, Alkhudary R, Zilic Z, Swidan A (2023). Performance analysis of a private blockchain network built on hyperledger fabric for healthcare. Inf Process Manag.

[324] Irresberger F, John K, Mueller P, Saleh F (2020). The public blockchain ecosystem: an empirical analysis.

[325] Ferdous MS, Chowdhury MJM, Hoque MA (2021). A survey of consensus algorithms in public blockchain systems for crypto-currencies. J Netw Comput Appl.

[326] Benhamouda F, Gentry C, Gorbunov S, Pass R, Pietrzak K (2020). Theory of Cryptography TCC 2020 Lecture Notes in Computer Science.

[327] Rebello GAF, Camilo GF, de Souza LAC (2024). A survey on blockchain scalability: from hardware to layer-two protocols. IEEE Commun Surv Tutorials.

[328] Dib O, Brousmiche KL, Durand A, Thea E, Hamida EB (2018). Consortium blockchains: overview, applications and challenges. Int J Adv Telecommun.

[329] Li Z, Kang J, Yu R, Ye D, Deng Q, Zhang Y (2017). Consortium blockchain for secure energy trading in industrial Internet of Things. IEEE Trans Ind Inf.

[330] Yao W, Ye J, Murimi R, Wang G (2021). A survey on consortium blockchain consensus mechanisms. arXiv.

[331] Zhang A, Lin X (2018). Towards secure and privacy-preserving data sharing in e-health systems via consortium blockchain. J Med Syst.

[332] Li D, Han D, Weng TH (2022). Blockchain for federated learning toward secure distributed machine learning systems: a systemic survey. Soft Comput.

[333] Houda ZAE, Hafid AS, Khoukhi L, Brik B (2022). When collaborative federated learning meets blockchain to preserve privacy in healthcare. IEEE Trans Netw Sci Eng.

[334] Issa W, Moustafa N, Turnbull B, Sohrabi N, Tari Z (2023). Blockchain-based federated learning for securing Internet of Things: a comprehensive survey. ACM Comput Surv.

[335] Passerat-Palmbach J, Farnan T, McCoy M Blockchain-orchestrated machine learning for privacy preserving federated learning in electronic health data.

[336] Aich S, Sinai NK, Kumar S Protecting personal healthcare record using blockchain & federated learning technologies.

[337] El Rifai O, Biotteau M, de Boissezon X, Megdiche I, Ravat F, Teste O, Michalowski M, Moskovitch R (2020). Artificial Intelligence in Medicine AIME 2020 Lecture Notes in Computer Science.

[338] Kim YJ, Hong CS Blockchain-based node-aware dynamic weighting methods for improving federated learning performance.

[339] Lu Y, Huang X, Zhang K, Maharjan S, Zhang Y (2020). Communication-efficient federated learning and permissioned blockchain for digital twin edge networks. IEEE Internet Things J.

[340] Pandey SR, Nguyen LD, Popovski P (2022). FedToken: tokenized incentives for data contribution in federated learning. arXiv.

[341] Behera MR, Upadhyay S, Shetty S (2021). Federated learning using smart contracts on blockchains, based on reward driven approach. arXiv.

[342] Ma S, Cao Y, Xiong L Transparent contribution evaluation for secure federated learning on blockchain.

[343] Wang Z, Hu Q (2021). Blockchain-based federated learning: a comprehensive survey. arXiv.

[344] Munusamy S, Jothi KR (2025). Blockchain-enabled federated learning with edge analytics for secure and efficient electronic health records management. Sci Rep.

[345] Castro M, Liskov B Practical Byzantine fault tolerance.

[346] Castro M, Liskov B (2002). Practical Byzantine fault tolerance and proactive recovery. ACM Trans Comput Syst.

[347] McMahan B, McMahan E, Ramage D, Hampson S, Aguera y Arcas B (2017). Proceedings of the 20th International Conference on Artificial Intelligence and Statistics.

[348] Buchman E (2016). Tendermint: Byzantine fault tolerance in the age of blockchains [Dissertation]. https://atrium.lib.uoguelph.ca/items/5459099e-67aa-4a23-83ae-d3471d8d8336.

[349] Hu K, Gong S, Zhang Q, Seng C, Xia M, Jiang S (2024). An overview of implementing security and privacy in federated learning. Artif Intell Rev.

[350] Xie M, Zhang Z, Hong H, Zhang G, Qin Y (2025). Secure medical data sharing featuring traceable data usage and automatic audit mechanism. IEEE Internet Things J.

[351] Li C, Xing Z, Liu J (2025). Integrating zero-knowledge proofs into federated learning: a path to on-chain verifiable and privacy-preserving federated learning frameworks. Int J Web Inf Syst.

[352] Han B, Li B, Jurdak R (2025). PBFL: a privacy-preserving blockchain-based federated learning framework with homomorphic encryption and single masking. IEEE Internet Things J.

[353] Lavin R, Liu X, Mohanty H, Norman L, Zaarour G, Krishnamachari B (2024). A survey on the applications of zero-knowledge proofs. arXiv.

[354] Zhou Z, Li Y, Wang Y ZHE: efficient zero-knowledge proofs for HE evaluations.

[355] Wang X, Chen T, Dai HN (2025). A privacy-enhanced method for privacy-preserving and verifiable federated learning. IEEE Internet Things J.

[356] Munjal K, Bhatia R (2023). A systematic review of homomorphic encryption and its contributions in healthcare industry. Complex Intell Syst.

[357] Kalapaaking AP, Stephanie V, Khalil I, Atiquzzaman M, Yi X, Almashor M (2022). SMPC-based federated learning for 6G-enabled Internet of Medical Things. IEEE Netw.

[358] Liu F, Zheng Z, Shi Y, Tong Y, Zhang Y (2024). A survey on federated learning: a perspective from multi-party computation. Front Comput Sci.

[359] Naresh VS, Venkata Raju A, Srinivasa Rao O (2025). Secure multiparty computation for privacy‐preserving machine learning in healthcare: a comprehensive survey. WIREs Computational Stats.

[360] Hosseini SM, Sikaroudi M, Babaei M, Tizhoosh HR, Albarqouni S (2022). Distributed, Collaborative, and Federated Learning, and Affordable AI and Healthcare for Resource Diverse Global Health DeCaF FAIR 2022 2022 Lecture Notes in Computer Science.

[361] Kalapaaking AP, Khalil I, Rahman MS, Atiquzzaman M, Yi X, Almashor M (2023). Blockchain-based federated learning with secure aggregation in trusted execution environment for Internet-of-Things. IEEE Trans Ind Inf.

[362] Zhu L, Hu S, Zhu X, Meng C, Huang M (2023). Enhancing the security and privacy in the IoT supply chain using blockchain and federated learning with trusted execution environment. Mathematics.

[363] Jiang J, Soriente C, Karame G On the challenges of detecting side-channel attacks in SGX.

[364] Guo J, Vaswani K, Paverd A, Pietzuch P (2025). VerifiableFL: verifiable claims for federated learning using exclaves. arXiv.

[365] Zhang L, Fang G, Tan Z (2025). FedCCW: a privacy-preserving Byzantine-robust federated learning with local differential privacy for healthcare. Cluster Comput.

[366] Tayyeh HK, AL-Jumaili ASA (2024). Balancing privacy and performance: a differential privacy approach in federated learning. Computers.

[367] Shukla S, Rajkumar S, Sinha A, Esha M, Elango K, Sampath V (2025). Federated learning with differential privacy for breast cancer diagnosis enabling secure data sharing and model integrity. Sci Rep.

[368] Cao X, Fang M, Liu J, Gong NZ FLTrust: Byzantine-robust federated learning via trust bootstrapping.

[369] Yan G, Wang H, Yuan X, Li J (2023). DeFL: defending against model poisoning attacks in federated learning via critical learning periods awareness. AAAI.

[370] Bagdasaryan E, Veit A, Hua Y, Estrin D, Shmatikov V (2020). Proceedings of the Twenty Third International Conference on Artificial Intelligence and Statistics.

[371] Xie C, Chen M, Chen PY, Li B (2021). Proceedings of the 38th International Conference on Machine Learning.

[372] Geiping J, Bauermeister H, Droge H, Moeller M (2020). Advances in Neural Information Processing Systems 33 (NeurIPS 2020).

[373] Hatamizadeh A, Yin H, Roth H GradViT: gradient inversion of vision transformers.

[374] Geng J, Mou Y, Li Q (2024). Improved gradient inversion attacks and defenses in federated learning. IEEE Trans Big Data.

[375] Nguyen T, Lai P, Tran K, Phan N, Thai MT (2023). Proceedings of The 26th International Conference on Artificial Intelligence and Statistics.

[376] Bai L, Hu H, Ye Q, Li H, Wang L, Xu J (2025). Membership inference attacks and defenses in federated learning: a survey. ACM Comput Surv.

[377] Commey D, Hounsinou SG, Crosby GV (2025). Post-quantum secure blockchain-based federated learning framework for healthcare analytics. IEEE Netw Lett.

[378] Li S, Ngai ECH, Voigt T (2024). An experimental study of Byzantine-robust aggregation schemes in federated learning. IEEE Trans Big Data.

[379] Bonawitz K, Ivanov V, Kreuter B Practical secure aggregation for privacy-preserving machine learning.

[380] McMahan HB, Ramage D, Talwar K, Zhang L (2017). Learning differentially private recurrent language models. arXiv.

[381] Geyer RC, Klein T, Nabi M (2017). Differentially private federated learning: a client level perspective. arXiv.

[382] Rangwala M, Venugopal KR, Buyya R (2025). Blockchain-enabled federated learning. arXiv.

[383] Fang F, Feng L, Xie J BCFL: a trustworthy and efficient federated learning framework based on blockchain in iot.

[384] Teo ZL, Jin L, Li S (2024). Federated machine learning in healthcare: a systematic review on clinical applications and technical architecture. Cell Rep Med.

[385] Li M, Xu P, Hu J, Tang Z, Yang G (2025). From challenges and pitfalls to recommendations and opportunities: implementing federated learning in healthcare. Med Image Anal.

[386] Salim MM, Yang LT, Park JH (2024). Privacy-preserving and scalable federated blockchain scheme for healthcare 4.0. Computer Networks.

[387] Moore SK (2024). Chips to compute with encrypted data are coming: fully homomorphic encryption could make data unhackable. IEEE Spectr.

[388] Karimireddy SP, He L, Jaggi M (2021). Proceedings of the 38th International Conference on Machine Learning.

[389] Komlo C, Goldberg I, Dunkelman O, Jacobson MJ, O’Flynn C (2021). Selected Areas in Cryptography SAC 2020 Lecture Notes in Computer Science.

[390] Pati S, Baid U, Edwards B (2022). The federated tumor segmentation (FeTS) tool: an open-source solution to further solid tumor research. Phys Med Biol.

[391] The HIPAA privacy rule. US Department of Health and Human Services.

[392] European Parliament and Council of the European Union (2016). General Data Protection Regulation (GDPR), Regulation (EU) 2016/679. EUR-Lex.

[393] Altameem A, Kovtun V, Al-Ma’aitah M, Altameem T, H F, Youssef AE (2022). Patient’s data privacy protection in medical healthcare transmission services using back propagation learning. Computers and Electrical Engineering.

[394] Artificial intelligence in software as a medical device. US Food and Drug Administration.

[395] Sinaci AA, Gencturk M, Alvarez-Romero C (2024). Privacy-preserving federated machine learning on FAIR health data: a real-world application. Comput Struct Biotechnol J.

[396] Tölle M, Garthe P, Scherer C (2025). Real world federated learning with a knowledge distilled transformer for cardiac CT imaging. NPJ Digit Med.

[397] Shick AA, Webber CM, Kiarashi N (2024). Transparency of artificial intelligence/machine learning-enabled medical devices. NPJ Digit Med.

[398] Leslie K, Moore J, Robertson C (2021). Regulating health professional scopes of practice: comparing institutional arrangements and approaches in the US, Canada, Australia and the UK. Hum Resour Health.

[399] Tan E, Lerouge E, Du Caju J, Du Seuil D (2023). Verification of education credentials on European blockchain services infrastructure (EBSI): action research in a cross-border use case between Belgium and Italy. BDCC.

[400] Orabi MM, Emam O, Fahmy H (2025). Adapting security and decentralized knowledge enhancement in federated learning using blockchain technology: literature review. J Big Data.

[401] Kalapaaking AP, Khalil I, Yi X, Lam KY, Huang GB, Wang N (2024). Auditable and verifiable federated learning based on blockchain-enabled decentralization. IEEE Trans Neural Netw Learning Syst.

[402] Kostick-Quenet KM, Compagnucci MC, Aboy M, Minssen T (2025). Patient-centric federated learning: automating meaningful consent to health data sharing with smart contracts. J Law Biosci.

[403] Siniosoglou I, Argyriou V, Sarigiannidis P (2023). Post-processing fairness evaluation of federated models: an unsupervised approach in healthcare. IEEE/ACM Trans Comput Biol and Bioinf.

[404] McKay F, Williams BJ, Prestwich G, Bansal D, Treanor D, Hallowell N (2023). Artificial intelligence and medical research databases: ethical review by data access committees. BMC Med Ethics.

[405] Zafar A (2025). Reconciling blockchain technology and data protection laws: regulatory challenges, technical solutions, and practical pathways. Journal of Cybersecurity.

[406] Belen-Saglam R, Altuncu E, Lu Y, Li S (2023). A systematic literature review of the tension between the GDPR and public blockchain systems. Blockchain: Research and Applications.

[407] Balistri E, Casellato F, Giannelli C, Stefanelli C (2021). BlockHealth: blockchain-based secure and peer-to-peer health information sharing with data protection and right to be forgotten. ICT Express.

[408] Makhdoom I, Zhou I, Abolhasan M, Lipman J, Ni W (2020). PrivySharing: a blockchain-based framework for privacy-preserving and secure data sharing in smart cities. Computers & Security.

[409] Zhang F, Shuai Z, Kuang K, Wu F, Zhuang Y, Xiao J (2024). Unified fair federated learning for digital healthcare. Patterns (N Y).

[410] Poulain R, Tarek MFB, Beheshti R (2023). Improving fairness in AI models on electronic health records: the case for federated learning methods. arXiv.

[411] Liu M, Ning Y, Teixayavong S (2025). A scoping review and evidence gap analysis of clinical AI fairness. NPJ Digit Med.

[412] Li S, Wu Q, Zhou D (2025). FairFML: fair federated machine learning with a case study on reducing gender disparities in cardiac arrest outcome prediction. NPJ Health Syst.

[413] Xing H, Sun R, Ren J (2025). Achieving flexible fairness metrics in federated medical imaging. Nat Commun.

[414] Friesen P, Douglas‐Jones R, Marks M (2021). Governing AI‐driven health research: are IRBs up to the task?. Ethics & Human Research.

[415] Capili B, Anastasi JK (2024). Ethical research and the institutional review board: an introduction. Am J Nurs.

[416] Bouderhem R (2024). Shaping the future of AI in healthcare through ethics and governance. Humanit Soc Sci Commun.

[417] (2021). Ethics and governance of artificial intelligence for health: WHO guidance. World Health Organization.

[418] Secretary’s Advisory Committee on Human Research Protections (2022). IRB considerations on the use of artificial intelligence in human subjects research. US Department of Health and Human Services.

[419] Kiseleva A, Kotzinos D, De Hert P (2022). Transparency of AI in healthcare as a multilayered system of accountabilities: between legal requirements and technical limitations. Front Artif Intell.

[420] Holzinger A, Saranti A, Molnar C, Biecek P, Samek W, Holzinger A, Goebel R, Fong R, Moon T, Müller KR, Samek W (2022). xxAI - Beyond Explainable AI xxAI 2020 Lecture Notes in Computer Science.

[421] Amann J, Blasimme A, Vayena E, Frey D, Madai VI, Precise4Q Consortium (2020). Explainability for artificial intelligence in healthcare: a multidisciplinary perspective. BMC Med Inform Decis Mak.

[422] Sadeghi Z, Alizadehsani R, Cifci MA (2024). A review of explainable artificial intelligence in healthcare. Computers and Electrical Engineering.

[423] Jobin A, Ienca M, Vayena E (2019). The global landscape of AI ethics guidelines. Nat Mach Intell.

[424] Rauter CM, Wöhlke S, Schicktanz S (2021). My data, my choice? - German patient organizations’ attitudes towards big data-driven approaches in personalized medicine. an empirical-ethical study. J Med Syst.

[425] Shen N, Kassam I, Zhao H (2022). Foundations for meaningful consent in Canada’s digital health ecosystem: retrospective study. JMIR Med Inform.

[426] Li N, Zhou C, Gao Y (2025). Machine unlearning: taxonomy, metrics, applications, challenges, and prospects. IEEE Trans Neural Netw Learning Syst.

[427] Liu Z, Jiang Y, Shen J (2025). A survey on federated unlearning: challenges, methods, and future directions. ACM Comput Surv.

[428] Bhardwaj T, Sumangali K (2025). An explainable federated blockchain framework with privacy-preserving AI optimization for securing healthcare data. Sci Rep.

[429] Blackman J, Veerapen R (2025). On the practical, ethical, and legal necessity of clinical artificial intelligence explainability: an examination of key arguments. BMC Med Inform Decis Mak.

[430] (2021). Artificial intelligence/machine learning (AI/ML)-based software as a medical device (SaMD) action plan. US Food and Drug Administration.

[431] Kauttonen J, Rousi R, Alamäki A (2025). Trust and acceptance challenges in the adoption of AI applications in health care: quantitative survey analysis. J Med Internet Res.

[432] Dayan I, Roth HR, Zhong A (2021). Federated learning for predicting clinical outcomes in patients with COVID-19. Nat Med.

[433] Kumar R, Khan AA, Kumar J (2021). Blockchain-federated-learning and deep learning models for COVID-19 detection using CT imaging. IEEE Sensors J.

[434] Pati S, Baid U, Edwards B (2022). Federated learning enables big data for rare cancer boundary detection. Nat Commun.

[435] Zenk M, Baid U, Pati S (2025). Towards fair decentralized benchmarking of healthcare AI algorithms with the federated tumor segmentation (FeTS) challenge. Nat Commun.

[436] Mazid A, Kirmani S, Abid M, Pawar V (2025). A secure and efficient framework for internet of medical things through blockchain driven customized federated learning. Cluster Comput.

[437] Moulahi W, Jdey I, Moulahi T, Alawida M, Alabdulatif A (2023). A blockchain-based federated learning mechanism for privacy preservation of healthcare IoT data. Comput Biol Med.

[438] Rahman MA, Hossain MS, Islam MS, Alrajeh NA, Muhammad G (2020). Secure and provenance enhanced Internet of Health Things framework: a blockchain managed federated learning approach. IEEE Access.

[439] Ahmed AA, Alabi OO (2024). Secure and scalable blockchain-based federated learning for cryptocurrency fraud detection: a systematic review. IEEE Access.

[440] Petrosino L, Masi L, D’Antoni F, Merone M, Vollero L (2025). A zero-knowledge proof federated learning on DLT for healthcare data. J Parallel Distrib Comput.

[441] Jiang Y, Ma B, Wang X (2024). Blockchained federated learning for Internet of Things: a comprehensive survey. ACM Comput Surv.

[442] Chang Y, Fang C, Sun W (2021). A blockchain-based federated learning method for smart healthcare. Comput Intell Neurosci.

[443] Zhang H, Jiang S, Xuan S (2024). Decentralized federated learning based on blockchain: concepts, framework, and challenges. Comput Commun.

[444] Samantray BS, Reddy KHK (2025). A federated learning approach towards hybrid blockchain, quantum-key-encryption based distributed system: a futuristic healthcare architecture for smart cities. Blockchain: Research and Applications.

[445] Bhasker B, Rao PM, Saraswathi P (2025). Blockchain framework with IoT device using federated learning for sustainable healthcare systems. Sci Rep.

[446] Ali AA, Gunavathie MA, Srinivasan V, Aruna M, Chennappan R, Matheena M (2025). Securing electronic health records using blockchain-enabled federated learning for IoT-based smart healthcare. Clinical eHealth.

[447] Das P, Kumar N, Jain C, Singh M (2025). Intelligent IoT-enabled healthcare solutions implementing federated meta-learning with blockchain. J Ind Inf Integr.

[448] Kumar R, Bernard CM, Ullah A (2024). Privacy-preserving blockchain-based federated learning for brain tumor segmentation. Comput Biol Med.

[449] Liang X, Zhao J, Chen Y, Bandara E, Shetty S (2023). Architectural design of a blockchain-enabled, federated learning platform for algorithmic fairness in predictive health care: design science study. J Med Internet Res.

[450] Om Kumar CU, Gajendran S, Balaji V, Nhaveen A, Sai Balakrishnan S (2023). RETRACTED ARTICLE: Securing health care data through blockchain enabled collaborative machine learning. Soft comput.

[451] Ali A, Al-Rimy BAS, Tin TT, Altamimi SN, Qasem SN, Saeed F (2023). Empowering precision medicine: unlocking revolutionary insights through blockchain-enabled federated learning and electronic medical records. Sensors (Basel).

[452] Lian Z, Wang W, Han Z, Su C (2023). Blockchain-based personalized federated learning for Internet of Medical Things. IEEE Trans Sustain Comput.

[453] Farooq K, Syed HJ, Alqahtani SO, Nagmeldin W, Ibrahim AO, Gani A (2022). Blockchain federated learning for in-home health monitoring. Electronics (Basel).

[454] Zhang H, Li G, Zhang Y, Gai K, Qiu M, Qiu H, Zhang C, Fei Z, Qiu M, Kung SY (2021). Knowledge Science, Engineering and Management KSEM 2021 Lecture Notes in Computer Science.

[455] Lo SK, Liu Y, Lu Q (2022). Toward trustworthy AI: blockchain-based architecture design for accountability and fairness of federated learning systems. IEEE Internet Things J.

[456] Singh S, Rathore S, Alfarraj O, Tolba A, Yoon B (2022). A framework for privacy-preservation of IoT healthcare data using federated learning and blockchain technology. Future Generation Computer Systems.

[457] Nguyen DC, Ding M, Pham QV (2021). Federated learning meets blockchain in edge computing: opportunities and challenges. IEEE Internet Things J.

[458] Liu Y, Yu W, Ai Z, Xu G, Zhao L, Tian Z (2022). A blockchain-empowered federated learning in healthcare-based cyber physical systems. IEEE Trans Netw Sci Eng.

[459] Otoum S, Al Ridhawi I, Mouftah HT (2021). Preventing and controlling epidemics through blockchain-assisted AI-enabled networks. IEEE Netw.

[460] Durga R, Poovammal E Federated learning model for healthchain system.

[461] Lakhan A, Mohammed MA, Nedoma J (2022). Federated-learning based privacy preservation and fraud-enabled blockchain IoMT system for healthcare. IEEE J Biomed Health Inform.

[462] Samuel O, Omojo AB, Onuja AM (2022). IoMT: a COVID-19 healthcare system driven by federated learning and blockchain. IEEE J Biomed Health Inform.

[463] Yang X, Xing C (2024). Federated medical learning framework based on blockchain and homomorphic encryption. Wireless Communications and Mobile Computing.

[464] Nguyen DC, Ding M, Pathirana PN, Seneviratne A (2021). Blockchain and AI-based solutions to combat coronavirus (COVID-19)-like epidemics: a survey. IEEE Access.

[465] Hemdan EED, Sayed A (2025). Smart and secure healthcare with digital twins: a deep dive into blockchain, federated learning, and future innovations. Algorithms.

[466] Baseri Y, Hafid A, Shahsavari Y, Makrakis D, Khodaiemehr H (2025). Blockchain security risk assessment in quantum era, migration strategies, and proactive defense. IEEE Commun Surv Tutorials.

[467] Cheon JH, Kim A, Kim M, Song Y, Takagi T, Peyrin T (2017). Advances in Cryptology – ASIACRYPT 2017 ASIACRYPT 2017 Lecture Notes in Computer Science.

[468] Doga H, Bose A, Sahin ME (2024). How can quantum computing be applied in clinical trial design and optimization?. Trends Pharmacol Sci.

[469] Thibault LT, Sarry T, Hafid AS (2022). Blockchain scaling using rollups: a comprehensive survey. IEEE Access.

[470] Hafid A, Hafid AS, Samih M (2020). Scaling blockchains: a comprehensive survey. IEEE Access.

[471] You J, Yang R, Zhan Y, Song B, Zhang Y, Wang Z (2025). BR-MTFL: a novel Byzantine resilience-enhanced multitask federated learning framework for high-speed train fault diagnosis. IEEE Trans Instrum Meas.

[472] Yang N, Tang C, Deng Z, He D (2025). A Gaussian reputation-based hybrid BFT consensus with a formal security framework. IEEE Trans Dependable and Secure Comput.

[473] Wang Y, Peng H, Su Z, Luan TH, Benslimane A, Wu Y (2022). A platform-free proof of federated learning consensus mechanism for sustainable blockchains. IEEE J Select Areas Commun.

[474] Ajmal CS, Yerram S, Abishek V (2025). Innovative approaches in regulatory affairs: leveraging artificial intelligence and machine learning for efficient compliance and decision-making. AAPS J.

[475] Dubey P, Kumar M (2025). Integrating explainable AI with federated learning for next-generation IoT: a comprehensive review and prospective insights. Computer Science Review.

[476] Smith V, Chiang CK, Sanjabi M, Talwalkar A Federated multi-task learning.

[477] Li K, Xiao C CBFL: a communication-efficient federated learning framework from data redundancy perspective. IEEE Systems Journal.

